# Design, Synthesis, and Biological Investigation of Novel Classes of 3-Carene-Derived Potent Inhibitors of TDP1

**DOI:** 10.3390/molecules25153496

**Published:** 2020-07-31

**Authors:** Irina V. Il’ina, Nadezhda S. Dyrkheeva, Alexandra L. Zakharenko, Alexander Yu. Sidorenko, Nikolay S. Li-Zhulanov, Dina V. Korchagina, Raina Chand, Daniel M. Ayine-Tora, Arina A. Chepanova, Olga D. Zakharova, Ekaterina S. Ilina, Jóhannes Reynisson, Anastasia A. Malakhova, Sergey P. Medvedev, Suren M. Zakian, Konstantin P. Volcho, Nariman F. Salakhutdinov, Olga I. Lavrik

**Affiliations:** 1 N.N. Vorozhtsov Novosibirsk Institute of Organic Chemistry, Siberian Branch of the Russian Academy of Sciences, 9, Lavrentiev Ave., 630090 Novosibirsk, Russia; ilyina@nioch.nsc.ru (I.V.I.); lizhulan@nioch.nsc.ru (N.S.L.-Z.); korchaga@nioch.nsc.ru (D.V.K.); anvar@nioch.nsc.ru (N.F.S.); 2 Institute of Chemical Biology and Fundamental Medicine, Siberian Branch of the Russian Academy of Sciences, 8, Lavrentiev Ave., 630090 Novosibirsk, Russia; elpida80@mail.ru (N.S.D.); sashaz@niboch.nsc.ru (A.L.Z.); arinachepanova@mail.ru (A.A.C.); isar@niboch.nsc.ru (O.D.Z.); katya.plekhanova@gmail.com (E.S.I.); amal@bionet.nsc.ru (A.A.M.); lavrik@niboch.nsc.ru (O.I.L.); 3 Institute of Chemistry of New Materials of National Academy of Sciences of Belarus, Skaryna Str, 36, 220141 Minsk, Belarus; camphene@gmail.com; 4 Nstitute of Cytology and Genetics, The Siberian Division of the Russian Academy of Sciences, Novosibirsk State University, 2, Pirogova Str., 630090 Novosibirsk, Russia; zakian@bionet.nsc.ru; 5 School of Chemical Sciences, The University of Auckland, Private Bag 92019, Victoria Street West, Auckland 1142, New Zealand; rcha387@aucklanduni.ac.nz (R.C.); dayi479@aucklanduni.ac.nz (D.M.A.-T.); 6 School of Pharmacy and Bioengineering, Keele University, Hornbeam Building, Staffordshire ST5 5BG, UK; j.reynisson@keele.ac.uk; 7 Federal Research Centre Institute of Cytology and Genetics of the Siberian Branch of Russian Academy of Sciences, 10, Lavrentiev Ave, 630090 Novosibirsk, Russia; medvedev@bionet.nsc.ru; 8 E.Meshalkin National medical research center of the Ministry of Health of the Russian Federation, 15 Rechkunovskaya Str., 630055 Novosibirsk, Russia

**Keywords:** monoterpene, carene, topotecan, tyrosyl-DNA phosphodiesterase 1, synergy, *TDP1* gene knockout cells, inhibitor

## Abstract

Two novel structural types of tyrosyl-DNA phosphodiesterase 1 (TDP1) inhibitors with hexahydroisobenzofuran **11** and 3-oxabicyclo [3.3.1]nonane **12** scaffolds were discovered. These monoterpene-derived compounds were synthesized through preliminary isomerization of (+)-3-carene to (+)-2-carene followed by reaction with heteroaromatic aldehydes. All the compounds inhibit the TDP1 enzyme at micro- and submicromolar levels, with the most potent compound having an IC_50_ value of 0.65 μM. TDP1 is an important DNA repair enzyme and a promising target for the development of new chemosensitizing agents. A panel of isogenic clones of the HEK293FT cell line knockout for the *TDP1* gene was created using the CRISPR-Cas9 system. Cytotoxic effects of topotecan (Tpc) and non-cytotoxic compounds of the new structures were investigated separately and jointly in the *TDP1* gene knockout cells. For two TDP1 inhibitors, **11h** and **12k**, a synergistic effect was observed with Tpc in the HEK293FT cells but was not found in TDP1 −/− cells. Thus, it is likely that the synergistic effect is caused by inhibition of TDP1. Synergy was also found for **11h** in other cancer cell lines. Thus, sensitizing cancer cells using a non-cytotoxic drug can enhance the efficacy of currently used pharmaceuticals and, concomitantly, reduce toxic side effects.

## 1. Introduction

There are a number of unresolved issues regarding chemotherapeutic treatment for oncological diseases, for example, low efficiency, resistance of malignant tumors, side effects, and high toxic load. The cytotoxic effect of chemotherapy is caused by DNA damage, and the ability of cancer cells to repair DNA lesions results in resistance. Therefore, the development of DNA repair enzyme inhibitors is a promising strategy to improve the efficacy of DNA damaging agents in clinical use. 

Tyrosyl-DNA-phosphodiesterase 1 (TDP1) is a DNA repair enzyme and a promising therapeutic target to enhance established cancer treatment [1,2,3,4]. TDP1 plays a key role in the removal of DNA lesions, in particular those caused by DNA topoisomerase I (TOP1) inhibitors. TOP1 is an essential enzyme that regulates DNA topology by reducing DNA supercoiling. TOP1 introduces a transient single-strand break in DNA, enabling the broken strand to rotate around the TOP1-bound DNA during fundamental cellular events, such as replication, transcription, and repair [5]. TOP1 inhibitors, for example, topotecan (Tpc) and irinotecan, both important chemotherapeutic agents, stabilize the cleavage complex TOP1–DNA, leading to cell death [6]. However, TDP1 removes the stalled TOP1-DNA complex, reducing the efficacy of the drugs and leading to resistance [7,8,9]. As has been confirmed in gene knockout studies in mice and human cell lines, TDP1 contributes to the development of drug resistance in a number of cancers (see [3] and references therein). Indeed, human cell lines with mutant or missing TDP1 as well as TDP1-knockout mice are both hypersensitive to the well-established TOP1 inhibitor camptothecin and its derivatives. Conversely, at high TDP1 expression levels, camptothecin and etoposide cause limited DNA damage [3]. Thus, a promising approach to increase the selectivity and potency of TOP1 poisons to cancer cells is to combine them with TDP1 inhibitors, which can significantly increase the chemotherapeutic efficacy. 

**Figure 1:**
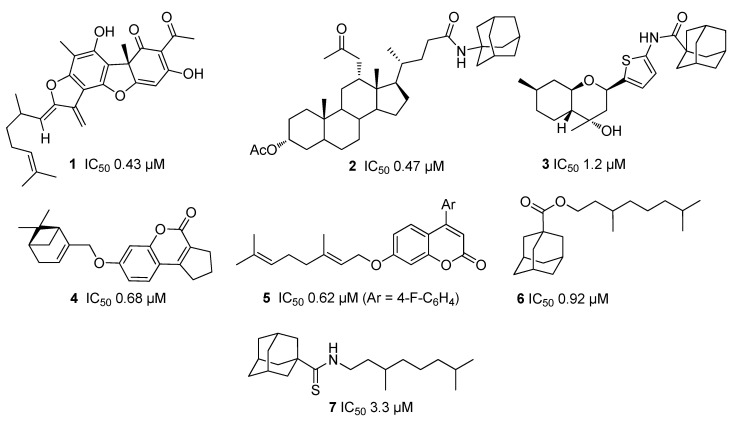
Structures of known TDP1 inhibitors and their half maximal inhibitory concentration (IC_50_) values.

In recent years, TDP1 inhibitors of various chemical types have been discovered [1,2,3,4], including natural products, such as usnic [10,11,12] and bile acids [13] (for example, compounds **1** and **2**, Figure 1). 

Monoterpenes, which have a unique diverse structure and are inexpensive, available and often enantiomerically pure, is an attractive renewable material for the development of physiologically active agents [14]. One of the approaches to utilize various monoterpenes, such as pinenes and *p*-menthenes, is their capability to react with carbonyl compounds, mainly aldehydes, resulting in heterocyclic compounds of different structural types [14,15]. Some of these derivatives exhibit analgesic, antiviral, neuroprotective, and antitumor properties [14]. Moreover, new classes of TDP1 inhibitors with submicromolar half maximal inhibitory concentration (IC_50_) values have been developed that contain monoterpene moieties; for example, coumarin [16], 4-arylcoumarin [17], and adamantane [18,19,20,21] derivatives as well as octahydro-2*H*-chromenes **3** (see Figure 1) synthesized from *p*-menthane monoterpenoid (-)-isopulegol [22]. Their ability to enhance the Tpc cytotoxic potential was demonstrated for several of these derivatives. For example, 7-hydroxycoumarin derivative **4** (Figure 1) with a pinene-type fragment reduced the CC_50_ (median cytotoxic concentration causing 50% cell death) of camptothecin eight-fold for the human breast adenocarcinoma cell line MCF-7 [16] while the hybrid molecule **5**, comprising of (4-fluorophenyl)-coumarin and geraniol moieties, showed a significant increase in the antitumor effect of Tpc on Krebs-2 ascites in an in vivo tumor model [17]. Among the adamantane derivatives, strong synergism was found for **6** and **7** (Figure 1), both containing the acyclic monoterpene substituents, when lung adenocarcinoma cells A549 were treated in combination with Tpc [18,20]. 

**Scheme 1:**
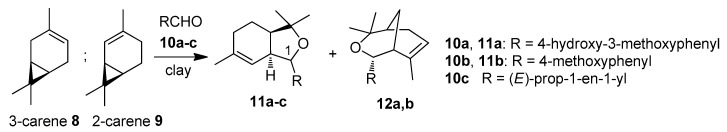
Reaction of carenes with aldehydes **10a**–**c** [23].

Monoterpene (+)-3-carene **8** (Scheme 1) is one of the main components of turpentine. Nevertheless, this compound is very rarely considered as a molecular platform for the development of new therapeutics and never considered for TDP1 inhibitors. In the current work, we studied the anti-TDP1 activity of (+)-3-carene derivatives for the first time. Two sets of (+)-3-carene derivatives with hexahydroisobenzofuran and 3-oxabicyclo [3.3.1]nonane skeletons were synthesized through preliminary isomerization of 3-carene to 2-carene followed by reaction with aldehydes. As a result, new structural types of potent TDP1 inhibitors were discovered. Furthermore, to elucidate the inhibitors’ mechanism of action on the cellular level, a panel of HEK293FT TDP1 knockout isogenic clones was created using the CRISPR-Cas9 approach and the cytotoxic potentials were measured.

## 2. Results and Discussion 

### 2.1. Chemistry

It has been shown that carene can be successfully reacted with carbonyl compounds [23,24]. Previously, we showed that condensation of 2-carene **9** with aldehydes **10a–c** in the presence of montmorillonite clays led to the formation of hexahydroisobenzofurans **11a–c** as a mixture of two isomers at the C(1) position and compounds with 3-oxabicyclo[3.3.1]nonane structure **12** as minor products (Scheme 1). It was found that hexahydroisobenzofuran **11a** obtained with condensation of 2-carene **9** with 4-hydroxy-3-methoxybenzaldehyde **10a** demonstrated potent neuroprotective activity in an in vivo Parkinson model combined with low acute toxicity [24]. The reactions of 3-carene **8** with aldehydes gave the same products **11** and **12** as it did for 2-carene **9** but in much lower yields (only 3% for the reaction with aldehyde **10a**), since 2-carene **9** is more reactive due to the conjugation of the double bond with the cyclopropane ring [23].

Interestingly, 2-carene **9** is widely found in nature albeit in small concentrations. The isolation of **9** from essential oils, or from reaction mixtures after 3-carene **8** isomerization, is difficult, making **9** expensive and therefore not attractive for use in organic synthesis [25,26,27,28,29]. Recently, we developed a method for the isomerization of readily available 3-carene **8** to 2-carene **9** using montmorillonite clays as a catalyst [30]. It was demonstrated that a mixture of carenes, limonene, and terpinenes containing 10% 2-carene can be effectively used as a starting material instead of pure 2-carene in a clay-catalyzed reaction with 4-hydroxy-3-methoxybenzaldehyde **10a**, resulting in chiral isobenzofurans **11a**. The largest yield of **11a** was obtained with the 2-carene-containing mixture (60%) [31], which is much higher than using pure 2-carene **9** (33%). At the same time, condensation of carenes with aldehydes has not yet been systematically studied. Here, we synthesized heterocyclic compounds using 2-carene from a mixture containing carenes, limonene, and terpinenes with 2-carene comprising 10%, by condensation with heteroaromatic furan and thiophene aldehydes. The choice of this aldehyde was based on the presence of furan and thiophene moieties in many bioactive compounds, including TDP1 inhibitors [32,33,34,35], as well as preliminary molecular modelling that indicated favourable binding to TDP1.

For the synthesis of heterocyclic compounds, we used a mixture of terpenes obtained by the isomerization of 3-carene **8** in the presence of montmorillonite K10 [31], which consists of 10% 2-carene, 30% 3-carene, 20% *m*- and *p*-cymols, 12% terpinenes, 5% limonene, and a number of dimeric compounds (*m/z* = 272) (according to gas chromatography-mass spectrometry (GC-MS) data). The reaction pathways for 3-carene isomerization are shown in Scheme S1, Supplementary Material.

**Scheme 2:**
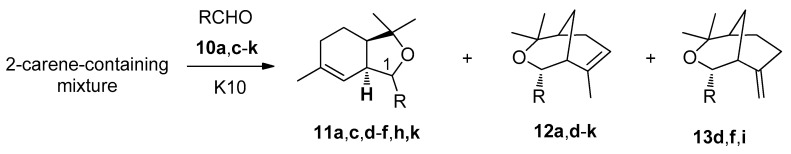
Reaction of 2-carene-containing mixture with aldehydes **10a**, **c**-**k**.

At first, we carried out the reactions with 4-hydroxy-3-methoxybenzaldehyde **10a** and crotonaldehyde **10c** previously studied using the 2-carene-containing mixture instead of neat 2-carene **9** [23]. In the present work, we used montmorillonite clay K10 as a catalyst, and the reaction was carried out without solvent at room temperature for 20 h. As a result of the reaction of 4-hydroxy-3-methoxybenzaldehyde **10a** and the 2-carene-containing mixture, isobenzofurans **11a** were obtained, which are diastereomers at position C(1) ((*S*)*-*/(*R*)- = 1:1, total yield 81%). In addition, compound **12a** (11%) was isolated (Scheme 2, Table 1).

**Scheme 3:**
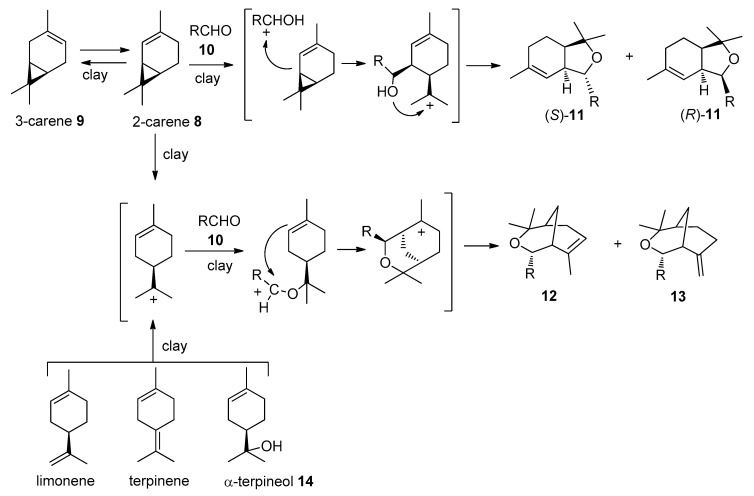
The proposed reaction mechanism for compounds **11**, **12**, and **13**.

The reactants for the products with the 3-oxabicyclo[3.3.1]nonane framework **12a** in reactions of the 2-carene-containing mixture with aldehydes can be both 2- and 3-carene as well as limonene and possibly terpinenes [15,23]; a proposed reaction mechanism for compounds **11**, **12**, and **13** is shown in Scheme 3. The yields for **11**, **12**, and **13** were calculated based on the 2-carene concentration in the mixture and on the total concentration of carenes, limonene, and terpinenes.

The reaction of crotonaldehyde **10c** with the 2-carene-containing mixture on K10 resulted in isobenzofurans **11c** ((*S*)/(*R*) = 1.3:1, total yield of 89%). The reaction yielded more **11c** than the 2-carene’s concentration in the mixture, with a higher product yield than for the reaction only using 2-carene **9**. It is therefore clear that the mixture can be successfully used without isolating the 2-carene **9**. We also studied the reactions of the 2-carene-containing mixture with the heteroaromatic aldehydes **10d**-**k** (thiophenecarboxaldehyde, furaldehyde, and their derivatives). Compound (*S*)-**11d** with a hexahydroisobenzofuran moiety was synthesized in a 78% yield, reacting the 2-carene-containing mixture with 2-thiophenecarboxaldehyde **10d** catalyzed by montmorillonite K10. In addition, a mixture of two compounds **12d** and **13d** with a 3-oxabicyclo[3.3.1]nonane skeletone was formed, which are isomers depending on the double bond position (**12d**/**13d** = 7:1, total yield 5%) (Scheme 2, Table 1). It should be noted that compound **11d**, in contrast to the previously studied reactions with aromatic aldehydes, the crotonaldehyde, formed exclusively as an (*S*)-isomer [23]. Moreover, although Prins cyclization in reactions of terpenoids with aldehydes, leading to the formation of heterocycles, with the 3-oxabicyclo[3.3.1]nonane framework is well studied [15,23], the only example of isolating a compound with an exocyclic double bond is the reaction of α-terpineol **14** with benzaldehyde in the presence of BF_3_∗Et_2_O, leading to **13d** [36].

The reaction of the 2-carene-containing mixture with methyl-substituted thiophene-2-carboxaldehydes **10e** and **10f** is similar to the previously mentioned reactions. Thus, starting from 3-methylthiophene-2-carboxaldehyde **10e**, compounds **11e** and **12e** were synthesized with yields of 86% and 2%, respectively; compound **13e** was formed only in trace amounts. Compound **11f** (85%) and a mixture of **12f** and **13f** (**12f**/**13f** = 3:1, 2%) were obtained in the reaction with 5-methylthiophene-2-carboxaldehyde **10f**. Compounds **11h** and **12h** were synthesized with yields of 73% and 35%, respectively, using 5-bromothiophen-2-carboxaldehyde **10h**. The reaction of the 2-carene-containing mixture with 4-bromothiophen-2-carboxaldehyde **10g** proceeded quite differently, as the only product of this reaction was compound **12g** with the 3-oxabicyclo[3.3.1]nonane framework with a 20% yield.

When 5-nitro-substituted heteroaromatic aldehydes were reacted with the 2-carene-containing mixture, compounds with the 3-oxabicyclo[3.3.1]nonane framework were only obtained. This can be explained by the low ability for protonation by these aldehydes, leading to high-energy barriers for the first stage of the isobenzofuran formation path (Scheme 3). Thus, by the reaction with 5-nitrothiophene-2-carboxaldehyde **13i** and 5-nitro-2-furaldehyde **13j**, compounds **12i**, **13i** (18%, **12i**/**13i** = 5:1), and **12j** (18%) were synthesized.

Interestingly, reaction of the 2-caren-containing mixture with 3-thiophenecarboxaldehyde **11k**, the isomer of 2-thiophenecarboxaldehyde **10d**, proceeded in a non-stereoseletive way with the formation of **11k** as a pair of diastereomers at the substituent position at C(1) in the ratio (*S*)-**11k**/(*R*)-**11k** = 1.5:1, with a total yield of 91%. Additionally, compound **12k** with 3-oxabicyclo[3.3.1]nonane skeletone (21%) was isolated from the reaction mixture.

It should be noted that in the case of the 2-carene-containing mixture reactions with aldehydes **10**, the main or even the only way of isobenzofuran synthesis was the formation of (*S*)-isomers (excluding the interaction with 4-hydroxy-3-methoxybenzaldehyde **10a**). Predominant formation of the (*S*)-isomer of isobenzofuran was clearly observed for the condensation of 2-carene and anisaldehyde [37]. According to denstity functional theory (DFT) calculations, the formation of (*S*)-isomer is energetically more favorable (by 6.8 kJ/mol) compared to formation of the (*R*)-isomer [37]. Moreover, the lower stability of the (*R*)-isomer in comparison with the (*S*)-isomer due to secondary transformations, leading to resinofication under the reaction conditions compared with (*S*)-isomer, was also shown [37].

Reactions of 2- and 3-furaldehyde as well as pyrrole-2-carboxaldehyde with the 2-carene-containing mixture did not lead to the formation of the target heterocyclic compounds. According to GC-MS data, after the reactions, 2-carene remained unconverted, but limonene was completely consumed and traces of compounds with *m/z* values corresponding to the intermolecular products, most likely with the 3-oxabicyclo[3.3.1]nonane skeleton, were observed. Apparently, the intermolecular products formed during the reaction are unstable and undergo degradation in the presence of an acid catalyst.

It should be noted that unreacted monoterpenes and other substances from the 2-carene-containing mixture are easily separated from the more polar condensation products by column chromatography using hexane as an elute. Moreover, compounds with the 3-oxabicyclo[3.3.1]nonane sceletone **12** can also be synthesized based on more available limonene [15]. For example, condensation of limonene with 2-thiophenecarboxaldehyde **10d** catalyzed by montmorillonite K10 without solvent at room temperature after 2 h led to the formation of compounds **12d** and **13d** (total yield 64%, **12d**/**13d** = 14:1).

Thus, we obtained a wide range of hydrogenated isobenzofuran and 3-oxabicyclo[3.3.1]nonane heterocycles, including compounds with an exocyclic double bond.

### 2.2. Biology

#### 2.2.1. Structure–Activity Relationship Analysis

The compounds synthesized starting from (+)-3-carene **8** were tested for their TDP1 inhibitory properties by measuring their IC_50_ values using a real-time fluorescent oligonucleotide biosensor [38]. This biosensor demonstrated high sensitivity and specificity for the detection of TDP1 activity. The commercially available TDP1 inhibitor furamidine, a bisbenzamidine derivative belonging to the diamidines family [34], was used as a positive control (IC_50_ 1.2 ± 0.3 μM). The results for all the derivatives are summarized in Table 2.

Compounds **11a**, **12a**, and **11c** with aromatic and alkyl substituents at position C(1) do not exhibit activity against TDP1, while the IC_50_ values for substances with heterocyclic substituents varied from 0.65 (**12g**) to 28 μM (**12j**) (Table 2).

Both **11** and **12,** with the thiophen-2-yl moiety, give good inhibition (IC_50_ values 4.85 μM (**11d**), 3.35 μM (**12d**)). The addition of a methyl group at the 3-position (compounds **11e**, **12e**) as well as at the 5-position (compound **11f**) on the thiophene ring had no effect.

The addition of bromine onto the heterocycle significantly increased the inhibition potency of the ligands. Thus, compound **11h** with a bromine atom at the 5-position of the thiophene ring had the best activity in the submicromolar range of the hexahydroisobenzofurans (IC_50_ 0.75 μM). From the compounds with the 3-oxabicyclo[3.3.1]nonane skeleton, the most active was 4-bromine-substituted compound **12g** (IC_50_ 0.65 μM). A bromine shift from position 4 to 5 from **12g** to **12h** decreased the inhibition potency (IC_50_ 1.75 μM); nevertheless, **12h** is approximately two-fold more active than **12d**, which does not have substituents on the thiophene ring.

Higher activity of the 3-substituted thiophenes **11k** and **12k** in comparison with 2-substituted thiophenes **11d** and **12d** was also observed. Interestingly, the 5-nitro-substituted thiophen- (**12i**) and furan (**12j**) with a 3-oxabicyclo[3.3.1]nonane skeleton did not exhibit high activity against TDP1.

#### 2.2.2. Cell Growth and Viability

##### Topotecan Cytotoxicity on HEK293FT Wild Type and TDP1−/− Cells

TDP1 plays an essential role in the resistance of cancer cells to antitumour TOP1-inhibiting drugs. It was shown previously for HEK293 [39] and other cell lines that cells lacking TDP1 expression exhibit hypersensitivity to camptothecin and its derivatives, such as Tpc [40]. To examine the potential impact of TDP1 knockout on cell survival and dosing with the synthesized compounds, we generated HEK293FT TDP1-deficient (TDP1−/−) cell line. Using a paired gRNA CRISPR-Cas9 strategy and polymerase chain reaction (PCR) screening of cell clones, we obtained three clones containing deletions in the first protein coding the exon of the *TDP1* gene. PCR products obtained from cell clones containing deletions were further cloned in a T-vector and 10 independent plasmid clones were sequenced by the Sanger method. Alignments of these deletions with the wild-type TDP1 gene sequence showed that modifications in all three clones lead to a shift in the reading frame of the gene and should lead to impaired synthesis of TDP1 (Figure S1). Clone B2 was found to have a 197-bp deletion in one of the TDP1 alleles, as well as a 16-bp deletion and T nucleotide insertion in the other allele; clone B5 carried 198-bp and 196-bp deletions; and the E3 clone had deletions of 197bp and 200bp. We treated TDP1-proficient (wild-type, WT) and TDP1-deficient (TDP1−/−) cells with increasing concentrations of Tpc. The results for all three clones are presented in Figure S2A. Clone E3 was selected for further work as it has a homozigous deletion causing an open reading frame shift in both alleles of the TDP1 gene and demonstrated sensitivity to Tpc. Clone E3 TDP1 mRNA was confirmed to have a 197/200-bp deletion causing the open reading frame shift (Figures S1, S2B). There is a deletion at the level of genomic DNA (Figure S2B, lane 4) and only the mutant form was transcribed (Figure S2B, lane 7). The clone E3 was subsequently screened by the biochemical assay for 3′-phosphotyrosyl cleavage activity to identify detectable TDP1 activity in the cell extract (Figure S2C). None was found in contrast to the control WT cell extract and purified TDP1.

**Figure 2:**
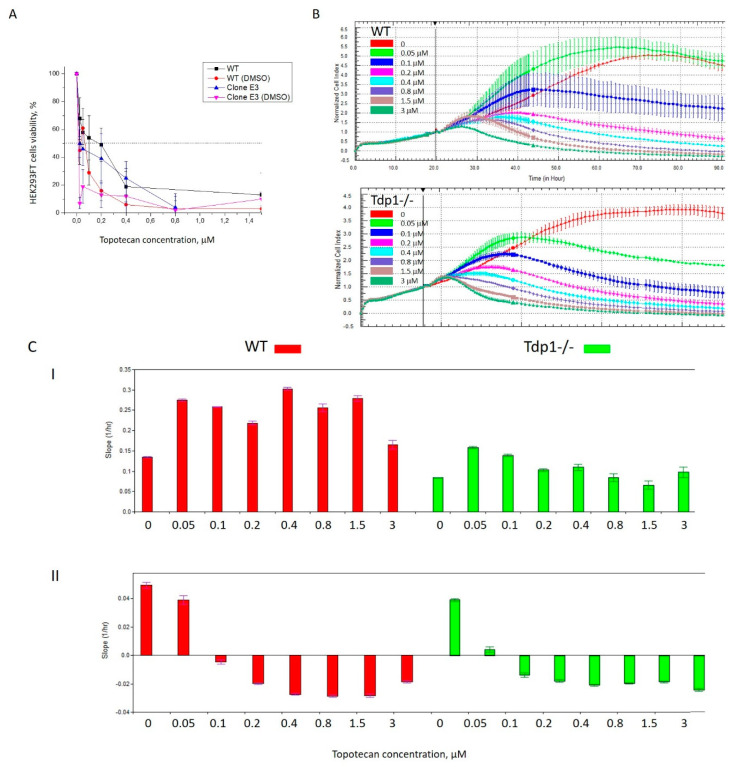
Topotecan (Tpc) cytotoxicity on HEK293FT WT and TDP1−/− knockout cells (**A**). Dose-dependent action of Tpc by the colorimetric test (**B**). Dose-dependent action of Tpc on cell viability by the impedance-based real-time assay on the xCELLigence System (**C**). The slope characterizing the velocity of cell growth with Tpc compared to the control for WT (red columns) and mutant cells (green columns): I) After the Tpc addition, II) 70 h after Tpc treatment. Real-time cell analysis demonstrates sensitivty to Tpc. Cell proliferation and viability are affected by different Tpc concentrations in the growth medium.

We analyzed, using a colorimetric test, the relative amount of viable cells after treating HEK293FT WT and TDP1-deficient (TDP1−/−) cells with increasing concentrations of Tpc for 72 h. TDP1 knockout reduced cell viability, where the IC_50_ values in H_2_O were 150 nM for TDP1−/− and 250 nM for WT cells (Figure 2A); and in DMSO, they were 30 nM for TDP1−/− and 200 nM for WT cells. Next, we studied WT and mutant cells’ live activity after Tpc treatment on the xCELLigence System (ACEA Biosciences, USA) by the impedance-based assay. HEK293FT TDP1−/− cells were more sensitive to Tpc (Figure 2B). After Tpc administration to the cells, an increase of the slope was seen, characterizing the velocity of the cell growth with Tpc as compared to the control both for WT (red columns) and mutant cells (green columns) (Figure S1, Supplementary Materials). The analysis of the averaged slope for the time period of ~70 h after Tpc treatment showed that the slope decreased depending on the Tpc concentration (Figure 2CII). The sharper decrease of the slope for the mutant cells than for the WT (compare the histogram for the 0 and 0.05 μM Tpc concentration, ~1.5 times for WT cells (red) and ~6 times for TDP1−/−cells (green)) also characterizes the higher sensitivity of the mutant to the TOP1 inhibitor. The Tpc IC_50_ values determined by the impedance-based assay were 60 nM for WT and 20 nM for TDP1−/− cells in H_2_O. There was a three-fold difference for these IC_50_ values, which is nearly the same as for the colorimetric assay. The absolute values slightly differed for these methods and solvents, but still, HEK293FT TDP1−/− cells showed a higher sensitivity to the TOP1 inhibitor Tpc, thus verifying the contribution of TDP1 on cell survival.

##### TDP1 Inhibitors’ Cytotoxicity on HEK293FT TDP1−/− Cells

**Figure 3:**
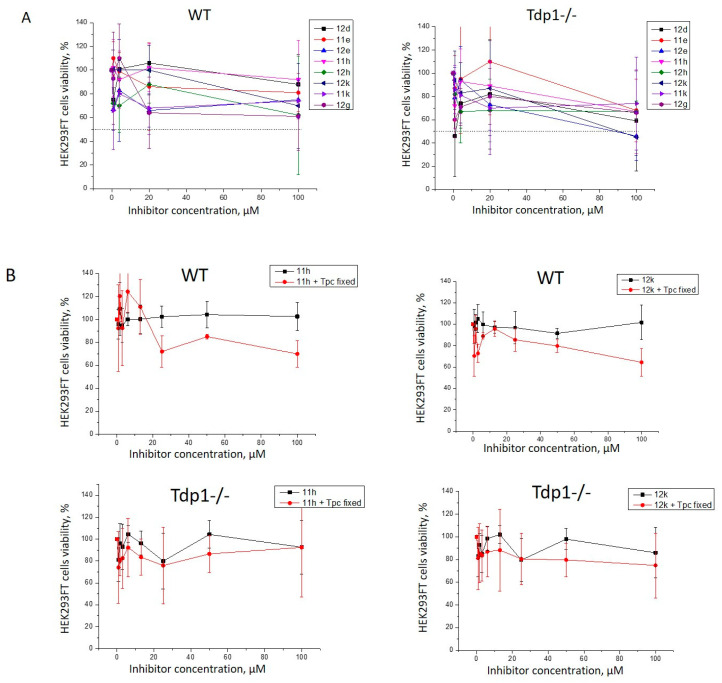
The TDP1 inhibitors’ intrinsic cytotoxicity on HEK293FT WT and TDP1−/− cells (**A**). Dose-dependent action of the derivatives (**B**). Activity with topotecan (Tpc) against HEK293FT cells in combination with **11h** and **12k**. The Tpc concentration was 30 nM for TDP1−/− and 200 nM for the WT cells.

An analysis of the intrinsic cytotoxicity of the synthesized compounds was performed on HEK293FT WT and TDP1-deficient (TDP1−/−) cell lines by a colorimetric test. We tested eight compounds with the highest TDP1 inhibitory activity: **11e** (3.6 μM), **11h** (0.75 μM), **11k** (1.6 μM), **12d** (IC_50_ 3.35 μM), **12e** (2.25 μM), **12g** (0.65 μM), **12h** (1.75 μM), and **12k** (1.2 μM). Interestingly, cytotoxicity was absent, or insignificant, in the range of concentrations (0.08–100 µM) for all the compounds in both cell lines (Figure 3A). The absence of toxicity is very important since it will not lead to undesirable side effects.

Next, we checked the influence of the eight most potent TDP1 inhibitors on the cytotoxic effect of Tpc on the WT and mutant cell lines. We chose 30 and 700 nM Tpc concentrations for the TDP1−/− and WT cells, respectively, which are close to the IC_50_ values for Tpc in DMSO in the colorimetric assay (Figure 2A). Then, we selected two candidates (**11h** and **12k**) for subsequent studies based on their ability to inhibit TDP1 activity and low cytotoxicity. We tested the HEK293FT cells’ viability after treatment with **11h** and **12k** and in conjunction with Tpc in a concentration close to its IC_50_ value (30 nM for TDP1−/− and 200 nM for WT cells). Suppressed cell growth was displayed with the TDP1 inhibitors and Tpc on the WT cells; however, no effect was seen for Tpc in TDP1-deficient cells (Figure 3B). Thus, the synergistic action of Tpc in conjunction with **11h** and **12k** on HEK293FT WT cells is most likely due to the TDP1 inhibition making it the main target of action. The initial increase in cell proliferation on the graphs up to the 20 µM concentration can be observed because we diluted the inhibitor with the medium before adding it to the well plate. Thus, a stimulation effect at low inhibitor concentrations could be observed due to the addition of fresh medium.

##### Activity of **11h** and **12k** with Topotecan against Tumor Cells

**Figure 4:**
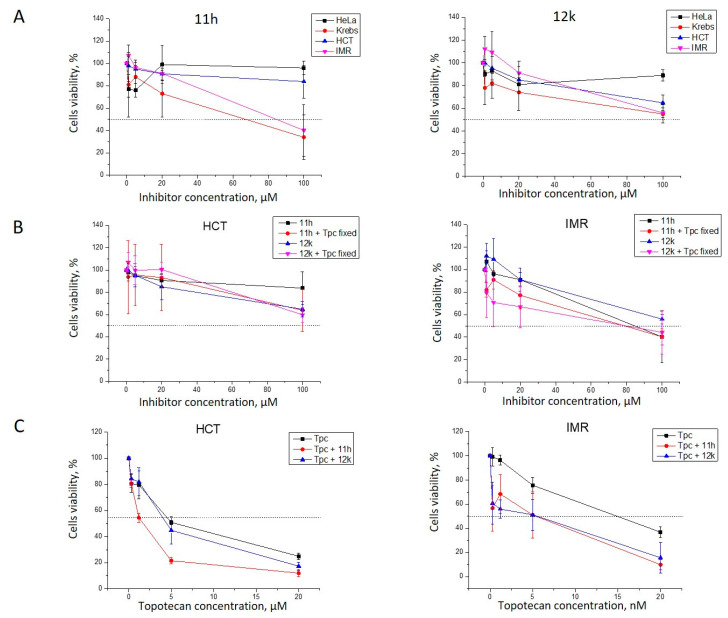
Activity of **11h** and **12k** with topotecan (Tpc) against tumor cells (**A**). The intrinsic cytotoxicity of **11h** and **12k** derivatives on the cancer cell lines (**B**). Dose-dependent action of **11h** and **12k** in combination with Tpc (**C**). Dose-dependent action of Tpc in conjunction with **11h** and **12k**.

Next, we checked the cytotoxicity of the combination of Tpc with **11h** and **12k** against several cancer cell lines and the results are shown in Figure 4.

The intrinsic cytotoxicity of both **11h** and **12k** on HeLa (cervical carcinoma), HCT116 (human colon cancer), IMR-32 (human neuroblastoma cell line), and Krebs-2 carcinoma cells was low (Figure 4A). Due to their low cytotoxicity, these derivatives were accepted as being suitable for further study on their cytotoxic effect with Tpc (Figure 4B,C). Both compounds showed no enhancement of the Tpc cytotoxicity for HeLa cells (data not shown). We compared HCT and IMR-32 cells’ viability after treatment with **11h** and **12k** alone and in the presence of Tpc in a concentration close to their IC_50_ values (900 nM, HCT; and 8 nM, IMR-32 cells). We observed suppressed cell growth in the joint presence of Tpc with **11h** or **12k** on the IMR-32 cells and no effect of Tpc on the HCT cell line (Figure 4B). We tested HCT and IMR-32 cells’ viability after Tpc treatment in the absence and presence of **11h**/**12k** (Figure 4C). Both **11h** and **12k** demonstrated synergistic action with Tpc on IMR-32 cell growth. **11h** also enhanced Tpc’s potency on HCT116 cells. These compounds show promise as candidates for anticancer therapy in conjunction with Tpc.

#### 2.2.3. Chemical Space

The calculated molecular descriptors MW (molecular weight), log *P* (water-octanol partition coefficient), HD (hydrogen bond donors), HA (hydrogen bond acceptors), PSA (polar surface area), and RB (rotatable bonds)) are given in Table S2. The molecular weight of the ligands lies between 206.3 and 327.3 g mol^−1^, falling into lead- and drug-like chemical spaces. The log *P* values range from 3.2 and 5.1, lying mostly in the drug-like but reaching into known drug space whilst the HD, HA, RB, and PSA values are within the lead-like space (for the definition of lead-like, drug-like, and known drug space (KDS) regions (see [41], Table S3).

The known drug index (KDI) of each ligand was calculated to gauge the balance of the molecular descriptors of the ligands. This method is based on the statistical analysis of drugs in clinical use (KDS) and a weighted index for each molecular descriptor. Both the summation (KDI_2a_) and multiplication (KDI_2b_) methods were used [42]; as shown for KDI_2a_ in Equation (1) and for KDI_2b_ in Equation (2). Finally, the numerical results are given in Table S4 in the Supplementary Material:

KDI_2a_ = I_MW_ + I_log P_ + I_HD_+ I_HA_ + I_RB_ + I_PSA,_ (1)

KDI_2b_ = I_MW_ × I_log P_ × I_HD_× I_HA_ × I_RB_ × I_PSA._ (2)

The KDI_2a_ values range from 4.15 to 5.15, with a theoretical maximum of 6 and an average of 4.08 for known drugs. KDI_2b_ ranges from 0.10 to 0.39, with a theoretical maximum of 1 and with a KDS average of 0.18. This indicates that the majority of the ligands are well balanced. **11h** has KDI_2a_ of 4.25 and KDI_2b_ of 0.11 and **12g** has similar values, with KDI_2a_ of 4.16 and KDI_2b_ of 0.10. The KDI_2a_ of both compounds is above the average index value for known drugs; however, the KDI_2b_ is below the average mainly due to the low PSA values. Nevertheless, these ligands can be considered reasonably well balanced in terms of their molecular descriptors.

## 3. Materials and Methods

### 3.1. Chemistry

All commercially available compounds and solvents were reagent grade and used without further treatment unless otherwise noted. We used montmorillonite K10 clay (Sigma-Aldrich, St. Louis, MO, USA) as the catalyst. The clay was calcinated at 105 °C for 3 h immediately before use. CH_2_Cl_2_ was passed through calcined Al_2_O_3_. The 2-carene-containing mixture was synthesized according to Sidorenko et al. [31] by isomerization of 3-carene (Acros Organics, 98%). Column chromatography (CC): silica gel (SiO_2_; 60–200 μ; Macherey-Nagel, Dueren, Germany); hexane/Et_2_O 100:0 → 90:10, analysis of fractions composition after column chromatography: GC on an Agilent 7820A, HP-5 quartz column, 30,000 × 0.25 mm, flame-ionization detector, He (1 atm) as the carrier gas. GC/MS (purity control and products analysis): Agilent 7890A with a quadrupole mass spectrometer Agilent 5975C as a detector, HP-5MS quartz column, 30,000 × 0.25 mm, He (1 atm) as the carrier gas. Optical rotation: *polAAr 3005* spectrometer, CHCl_3_ soln. HR-MS: *DFS-Thermo-Scientific* spectrometer in the full scan mode (15–500 *m*/*z*, 70 eV electron-impact ionization, direct sample introduction). ^1^H and ^13^C NMR: *Bruker DRX-500* apparatus at 500.13 MHz (^1^H) and 125.76 MHz (^13^C) and *Bruker* Avance-III 600 apparatus at 600.30 MHz (^1^H) and 150.95 MHz (^13^C) in CDCl_3_; chemical shifts δ in ppm rel. to residual CHCl_3_ (δ (H) 7.24, δ (C) 76.90 ppm), *J* in Hz; structure determinations by analyzing the ^1^H NMR spectra, including ^1^H – ^1^H double resonance spectra and ^1^H – ^1^H 2D homonuclear correlation (COSY, NOESY); J-modulated ^13^C NMR spectra (JMOD), and ^13^C – ^1^H 2D heteronuclear correlation with one-bond and long-range spin-spin coupling constants (C – H COSY, ^1^*J*(C,H) = 135 Hz; HSQC, ^1^*J*(C,H) = 145 Hz; COLOC, ^2,3^*J*(C,H) = 10 Hz; HMBC, ^2,3^*J*(C,H) = 7 Hz). All the target compounds reported have a purity of ≥95%. Numeration of atoms in the compounds (see Supplementary Material) is given for assigning the signals in the NMR spectra and does not coincide with that for the names according to the nomenclature of compounds. Spectral and analytical studies were carried out at the Collective Chemical Service Center of the Siberian Branch of the Russian Academy of Sciences.

**Figure 5:**
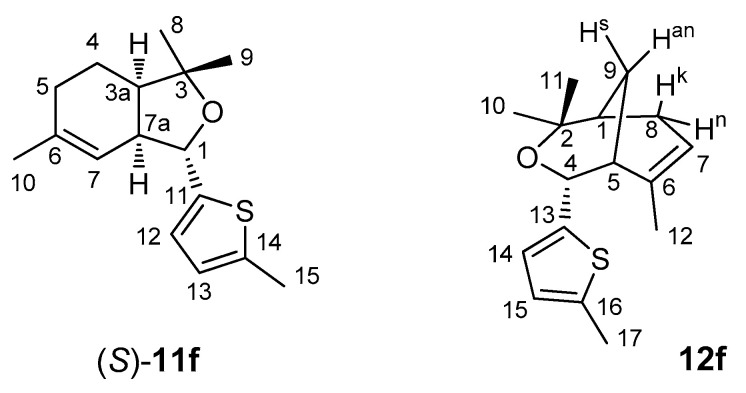
Numeration of carbon atoms for NMR spectra.

Numeration for the carbon atoms used for assignment in NMR spectra is presented in Figure 5 with compound (*S*)-**11f** and **12f** as an example.

#### 3.1.1. General Procedure (GP)

To the suspension of the K10 clay (3.0 g) in methylene chloride (15 mL) solution of 0.40 g aldehyde in methylene chloride (10 mL) and 1.0 g of a 2-carene containing mixture (10 wt.% of 2-carene., 57 wt.% of the sum of 2- and 3-carenes, limonene, and menthadienes., obtained by isomerization of 3-carene according to [30,31], were added. The solvent was distilled off, and the reaction mixture was kept at rt for 20 h. Thereafter, ethyl acetate was added, the catalyst was filtered off, and the filtrate was evaporated. Isolation of the reaction products was carried out by column chromatography (silica gel (17 g), hexane/Et_2_O 100:0 → 90:10). Compounds **11** were eluted with a 4–6% solution of Et_2_O in hexane, and compounds **12** and **13** with a 0.5–2% solution of Et_2_O in hexane. The yields of compounds **11**, **12**, and **13** were calculated based on the 2-carene content and on the sum of 2- and 3-carenes, limonene, and menthadienes, respectively.

#### 3.1.2. Reaction of 2-Carene-Containing Mixture and 4-hydroxy-3-methoxybenzaldehyde **10a**

According to the GP, the reaction of the 2-carene containing mixture and 4-hydroxy-3-methoxybenzaldehyde **10a** gave isobenzofurans **11a** (0.172 g, total yield 81%, (*S*)*-*/(*R*)- = 1:1), and compound **12a** (0.135 g, 11%) was isolated. The spectrum of the substance (*S*)-11a, (*R*)-11a, and **12a** corresponds to the literature [23].

#### 3.1.3. Reaction of 2-Carene-Containing Mixture and Crotonaldehyde **10c**

According to the GP, the reaction of the 2-carene containing mixture and crotonaldehyde **10c** gave isobenzofurans **11c** (0.135 g, total yield of 89%, (*S*)/(*R*) = 1.3: 1). The spectrum of the substance (*S*)-11c and (*R*)-10c corresponds to the literature [23].

#### 3.1.4. Reaction of 2-Carene-Containing Mixture and 2-Thiophenecarbaldehyde **10d**

According to the GP, the reaction of the 2-carene containing mixture and 2-thiophenecarbaldehyde **10d** gave (1*S*,3a*R*,7a*S*)-3,3,6-trimethyl-1-(thiophen-2-yl)-1,3,3a,4,5,7a-hexahydroisobenzofuran **11d** (0.143 g, 78%) and the mixture of (1*R*,4*R*,5*R*)-2,2,6-trimethyl-4-(thiophen-2-yl)-3-oxabicyclo[3.3.1]non-6-ene **12d** and (1*R*,4*R*,5*R*)-2,2-dimethyl-6-methylene-4-(thiophen-2-yl)-3-oxabicyclo[3.3.1]nonane **13d** (0.050 g, 5%, 7:1).

**11d**: NMR ^1^H (600 MHz, CDCl_3_, δ, ppm, *J*/Hz): 1.28 (s, 3H, Me-9), 1.39 (s, 3H, Me-8), 1.51–1.60 (m, 1H, H_a_-4), 1.68 (br.s, 3H, Me-10), 1.74 (dm, 1H, ^2^*J* = 13.0, H_e_-4), 1.95–2.01 (m, 3H, 2H-5, H-3a), 2.83–2.88 (m, 1H, H-7a), 4.85 (d, 1H, *J_1,7a_* = 10.0, H-1), 5.27–5.29 (m, 1H, H-7), 6.93 (dd, 1H, *J_13,14_* = 5.0, *J_13,12_* = 3.5, H-13), 6.96 (dm, 1H, *J_12,13_* = 3.5, H-12), 7.22 (dd, 1H, *J_14,13_* = 5.0, *J_14,12_* = 1.2, H-14). NMR ^13^C (150 MHz, CDCl_3_, δ, ppm): 81.33 (d, C-1), 83.05 (c, C-3), 46.38 (d, C-3a), 21.83 (t, C-4), 29.98 (t, C-5), 136.55 (c, C-6), 118.41 (d, C-7), 48.68 (d, C-7a), 30.92 (q, C-8), 24.00 (q, C-9), 23.49 (q, C-10), 146.76 (s, C-11), 123.89 (d, C-12), 126.42 (d, C-13), 124.26 (d, C-14). HR-MS: m/z calcd. for C_15_H_20_OS: 248.1229. Found: 248.1227. ${\left[\alpha \right]}_{D}^{30}$ = 120 (c = 0.3, CHCl_3_).

The NMR spectra of **12d** and **13d** were recorded for their mixture (≈ 7:1).

**12d**: NMR ^1^H (600 MHz, CDCl_3_, δ, ppm, *J*/Hz): 1.00–1.03 (m, 3H, Me-12), 1.30 (s, 3H, Me-10), 1.37 (s, 3H, Me-11), 1.51–1.56 (m, 1H, H-1), 1.71 (ddd, 1H, ^2^*J* = 12.5, *J_9an,1_* = *J_9an,5_* = 3.2, H-9an), 2.08 (dm, 1H, *J_8k,8n_* = 18.8, H-8k), 2.21 (ddd, 1H, *J_5,9s_* = *J_5,9an_* = 3.2, *J_5,4_* = 2.3, H-5), 2.30 (dddd, 1H, ^2^*J* = 12.5, *J_9s,1_* = *J_9s,5_* = 3.2, *J_9s,8n_* = 1.2, H-9s), 2.38 (br.d, 1H, ^2^*J* = 18.8, H-8n), 5.14 (d, 1H, *J_4,5_* = 2.3, H-4), 5.46–5.49 (m, 1H, H-7), 6.89 (dm, 1H, *J_14,15_* = 3.5, H-14), 6.91 (dd, 1H, *J_15,16_* = 5.0, *J_15,14_* = 3.5, H-15), 7.12 (dd, 1H, *J_16,15_* = 5.0, *J_16,14_* = 1.3, H-16). NMR ^13^C (150 MHz, CDCl_3_, δ, ppm): 33.86 (d, C-1), 75.89 (c, C-2), 71.94 (d, C-4), 42.31 (d, C-5), 133.04 (c, C-6), 123.46 (d, C-7), 27.55 (t, C-8), 27.74 (t, C-9), 28.44 (q, C-10), 23.87 (q, C-11), 23.97 (q, C-12), 146.97 (c, C-13), 121.54 (d, C-14), 126.04 (d, C-15), 123.05 (d, C-16). HR-MS: *m*/*z* calcd. for C_15_H_20_OS: 248.1229. Found: 248.1230.

**13d**: NMR ^1^H (600 MHz, CDCl_3_, δ, ppm, *J*/Hz): 1.36 (s, 3H, Me-10), 1.40 (s, 3H, Me-11), 1.50–1.53 (m, 1H, H-1), 1.72–1.77 (m, 1H, H-9s), 2.86–2.93 (m, 1H, H_a_-7), 4.31 (dd, 1H, ^2^*J* = 2.3, *J_12,5_* = 2.3, H-12), 4.56 (dd, 1H, ^2^*J* = 2.3, *J_12′,5_* = 2.3, H-12′), 5.22 (br.d, 1H, *J_4,5_* = 1.9, H-4), 6.87 (dm, 1H, *J_14,15_* = 3.5, H-14), 7.07–7.10 (m, 1H, H-15), 7.11–7.13 (m, 1H, H-16). The remaining proton signals are overlapped by the signals of the main isomer **12a**. NMR ^13^C (150 MHz, CDCl_3_, δ, ppm): 35.10 (d, C-1), 75.99 (c, C-2), 73.46 (d, C-4), 46.00 (d, C-5), 148.29 (c, C-6), 31.08 (t, C-7), 28.92 (t, C-8), 32.05 (t, C-9), 27.85 (q, C-10), 24.29 (q, C-11), 109.69 (t, C-12), 148.29 (c, C-13), 122.40 (d, C-14), 125.66 (d, C-15), 129.45 (d, C-16). HR-MS: *m*/*z* calcd. for C_15_H_20_OS: 248.1229. Found: 248.1230.

#### 3.1.5. Reaction of 2-Carene-Containing Mixture and 3-methylthiophene-2-carbaldehyde **10e**

According to the GP, the reaction of the 2-carene-containing mixture and 3-methylthiophene-2-carbaldehyde **10e** gave (1*S*,3a*R*,7a*S*)-3,3,6-trimethyl-1-(3-methylthiophen-2-yl)-1,3,3a,4,5,7a-hexahydroisobenzofuran **11e** (0.167 g, 86%) and (1*R*,4*R*,5*R*)-2,2,6-trimethyl-4-(3-methylthiophen-2-yl)-3-oxabicyclo[3.3.1]non-6-ene **12e** (0.025 g, 2%).

**11e**: NMR ^1^H (600 MHz, CDCl_3_, δ, ppm, *J*/Hz): 1.28 (s, 3H, Me-9), 1.41 (s, 3H, Me-8), 1.57–1.65 (m, 1H, H_a_-4), 1.67 (br.s, 3H, Me-10), 1.74 (dm, 1H, ^2^*J* = 12.9, H_e_-4), 1.96–2.02 (m, 3H, 2H-5, H-3a), 2.17 (s, 3H, Me-15), 2.84–2.90 (m, 1H, H-7a), 4.92 (d, 1H, *J_1,7a_* = 10.1, H-1), 5.21–5.24 (m, 1H, H-7), 6.74 (d, 1H, *J_13,14_* = 5.0, H-13), 7.13 (d, 1H, *J_14,13_* = 5.0, H-14).

NMR ^13^C (150 MHz, CDCl_3_, δ, ppm): 79.14 (d, C-1), 82.64 (c, C-3), 46.40 (d, C-3a), 21.92 (t, C-4), 30.02 (t, C-5), 136.38 (c, C-6), 118.47 (d, C-7), 49.18 (d, C-7a), 31.14 (q, C-8), 24.03 (q, C-9), 23.45 (q, C-10), 139.46 (s, C-11), 134.13 (s, C-12), 129.79 (d, C-13), 123.04 (d, C-14), 13.88 (q, C-15). HR-MS: m/z calcd. for C_16_H_22_OS: 262.1386. Found: 262.1388. ${\left[\alpha \right]}_{D}^{30}$ = 100 (c = 0.6, CHCl_3_).

**12e**: NMR ^1^H (600 MHz, CDCl_3_, δ, ppm, *J*/Hz): 0.97–1.00 (m, 3H, Me-12), 1.29 (s, 3H, Me-10), 1.37 (s, 3H, Me-11), 1.51–1.55 (m, 1H, H-1), 1.70 (ddd, 1H, ^2^*J* = 12.5, *J_9an,1_* = *J_9an,5_* = 3.1, H-9an), 2.04–2.09 (m, 1H, H-8k), 2.20 (s, 3H, Me-17), 2.20–2.22 (m, 1H, H-5), 2.28 (dddd, 1H, ^2^*J* = 12.5, *J_9s,1_* = *J_9s,5_* = 3.1, *J_9s,8n_* = 1.2, H-9s), 2.39 (br.d, 1H, ^2^*J* = 18.4, H-8n), 5.10 (d, 1H, *J_4,5_* = 2.5, H-4), 5.46–5.49 (m, 1H, H-7), 6.71 (d, 1H, *J_15,16_* = 5.0, H-15), 7.04 (d, 1H, *J_16,15_* = 5.0, H-16).

NMR ^13^C (150 MHz, CDCl_3_, δ, ppm): 33.79 (d, C-1), 75.79 (c, C-2), 71.15 (d, C-4), 39.12 (d, C-5), 133.11 (c, C-6), 123.51 (d, C-7), 27.44 and 27.49 (2t, C-8, C-9), 28.47 (q, C-10), 23.89 (q, C-11), 23.61 (q, C-12), 140.20 (c, C-13), 129.93 (s, C-14), 129.03 (d, C-15), 122.72 (d, C-16), 14.09 (q, C-17). HR-MS: *m*/*z* calcd. for C_16_H_22_OS: 262.1386. Found: 262.1384. ${\left[\alpha \right]}_{D}^{30}$ = 0 (c = 0.3, CHCl_3_).

#### 3.1.6. Reaction of 2-Carene-Containing Mixture and 5-methylthiophene-2-carbaldehyde **10f**

According to the GP, the reaction of the 2-carene-containing mixture and 5-methylthiophene-2-carbaldehyde **10f** gave (1*S*,3a*R*,7a*S*)-3,3,6-trimethyl-1-(5-methylthiophen-2-yl)-1,3,3a,4,5,7a-hexahydroisobenzofuran **11f** (0.163 g, 85%) and mixture of (1*R*,4*R*,5*R*)-2,2,6-trimethyl-4-(5-methylthiophen-2-yl)-3-oxabicyclo[3.3.1]non-6-ene **12f** and (1*R*,4*R*,5*R*)-2,2-dimethyl-6-methylene-4-(5-methylthiophen-2-yl)-3-oxabicyclo[3.3.1]nonane **13f** (0.025 g, 2%, 3:1).

**11f**: NMR ^1^H (600 MHz, CDCl_3_, δ, ppm, *J*/Hz): 1.27 (s, 3H, Me-9), 1.38 (s, 3H, Me-8), 1.51–1.59 (m, 1H, H_a_-4), 1.68 (br.s, 3H, Me-10), 1.73 (dm, 1H, ^2^*J* = 12.9, H_e_-4), 1.94–2.01 (m, 3H, 2H-5, H-3a), 2.44 (d, 3H, *J_15,13_* = 0.8, Me-15), 2.82–2.87 (m, 1H, H-7a), 4.75 (d, 1H, *J_1,7a_* = 10.0, H-1), 5.27–5.30 (m, 1H, H-7), 6.56 (dq, 1H, *J_13,12_* = 3.5, *J_13,15_* = 0.8, H-13), 6.74 (d, 1H, *J_12,13_* = 3.5, H-12). NMR ^13^C (150 MHz, CDCl_3_, δ, ppm): 81.45 (d, C-1), 82.78 (c, C-3), 46.31 (d, C-3a), 21.81 (t, C-4), 29.97 (t, C-5), 136.34 (c, C-6), 118.54 (d, C-7), 48.41 (d, C-7a), 30.92 (q, C-8), 23.98 (q, C-9), 23.45 (q, C-10), 144.02 (s, C-11), 124.15 (d, C-12), 124.38 (d, C-13), 138.92 (s, C-14), 15.29 (q, C-15). HR-MS: *m*/*z* calcd. for C_16_H_22_OS: 262.1386. Found: 262.1382. ${\left[\alpha \right]}_{D}^{30}$ = 119 (c = 0.4, CHCl_3_).

The NMR spectra of **12f** and **13f** were recorded for their mixture (≈ 3:1).

**12f**: NMR ^1^H (600 MHz, CDCl_3_, δ, ppm, *J*/Hz): 1.10–1.12 (m, 3H, Me-12), 1.28 (s, 3H, Me-10), 1.35 (s, 3H, Me-11), 1.50–1.53 (m, 1H, H-1), 1.69 (ddd, 1H, ^2^*J* = 12.5, *J_9an,1_* = *J_9an,5_* = 3.2, H-9an), 2.06 (dm, 1H, *J_8k,8n_* = 18.8, H-8k), 2.17 (ddd, 1H, *J_5,9s_* = *J_5,9an_* = 3.2, *J_5,4_* = 2.0, H-5), 2.27 (dddd, 1H, ^2^*J* = 12.5, *J_9s,1_* = *J_9s,5_* = 3.2, *J_9s,8n_* = 1.2, H-9s), 2.36 (br.d, 1H, ^2^*J* = 18.8, H-8n), 2.39 (br.s, 3H, Me-17), 5.04 (br.d, 1H, *J_4,5_* = 2.0, H-4), 5.45–5.48 (m, 1H, H-7), 6.53–6.55 (m, 1H, H-15), 6.65 (dd, 1H, *J_14,15_* = 3.3, *J_14,4_* = 1.0, H-14). NMR ^13^C (150 MHz, CDCl_3_, δ, ppm): 33.87 (d, C-1), 75.78 (c, C-2), 71.93 (d, C-4), 42.22 (d, C-5), 133.29 (c, C-6), 123.28 (d, C-7), 27.56 (t, C-8), 27.74 (t, C-9), 28.46 (q, C-10), 23.87 (q, C-11), 24.20 (q, C-12), 144.32 (c, C-13), 121.31 (d, C-14), 124.03 (d, C-15), 137.35 (s, C-16), 15.06 (q, C-17). HR-MS: *m*/*z* calcd. for C_16_H_22_OS: 262.1386. Found: 262.1387. ${\left[\alpha \right]}_{D}^{30}$ = 0 (c = 0.2, CHCl_3_).

**13f**: NMR ^1^H (600 MHz, CDCl_3_, δ, ppm, *J*/Hz): 1.33 (s, 3H, Me-10), 1.38 (s, 3H, Me-11), 1.47–1.50 (m, 1H, H-1), 2.09–2.16 (m, 2H, H-7e, H-8e), 2.36–2.46 (m, 2H, H-9an, H-5), 2.39 (br.s, 3H, Me-17), 2.86–2.93 (m, 1H, H_a_-7), 4.37 (dd, 1H, ^2^*J* = 2.4, *J_12,5_* = 2.4, H-12), 4.61 (dd, 1H, ^2^*J* = 2.4, *J_12_*_′*,5*_ = 2.4, H-12′), 5.11 (br.d, 1H, *J_4,5_* = 1.9, H-4), 6.53–6.55 (m, 1H, H-15), 6.65 (dd, 1H, *J_14,15_* = 3.3, *J_14,4_* = 1.0, H-14). The remaining proton signals are overlapped by the signals of the main isomer **12f**. NMR ^13^C (150 MHz, CDCl_3_, δ, ppm): 35.12 (d, C-1), 75.88 (c, C-2), 73.52 (d, C-4), 45.79 (d, C-5), 148.56 (c, C-6), 31.17 (t, C-7), 29.02 (t, C-8), 32.21 (t, C-9), 27.88 (q, C-10), 24.28 (q, C-11), 109.72 (t, C-12), 143.30 (c, C-13), 122.50 (d, C-14), 123.98 (d, C-15), 137.68 (s, C-16), 15.12 (q, C-17). HR-MS: *m*/*z* calcd. for C_16_H_22_OS: 262.1386. Found: 262.1387.

#### 3.1.7. Reaction of 2-Carene-Containing Mixture and 4-bromothiophene-2-carbaldehyde **10g**

According to the GP, the reaction of the 2-carene-containing mixture and 4-bromothiophene-2-carbaldehyde **10g** gave (1*R*,4*R*,5*R*)-2,2,6-trimethyl-4-(4-bromothiophen-2-yl)-3-oxabicyclo[3.3.1]non-6-ene **12g** (0.273 g, 20%).

**12g**: NMR ^1^H (600 MHz, CDCl_3_, δ, ppm, *J*/Hz): 1.06–1.08 (m, 3H, Me-12), 1.28 (s, 3H, Me-10), 1.34 (s, 3H, Me-11), 1.50–1.54 (m, 1H, H-1), 1.70 (ddd, 1H, ^2^*J* = 12.5, *J_9an,1_* = *J_9an,5_* = 3.2, H-9an), 2.06 (dm, 1H, ^2^*J* = 18.8, H-8k), 2.16–2.19 (m, 1H, H-5), 2.27 (dm, 1H, ^2^*J* = 12.5, H-9s), 2.35 (br.d, 1H, ^2^*J* = 18.8, H-8n), 5.05 (dd, 1H, *J_4,5_* = 2.5, *J_4,14_* = 0.7, H-4), 5.46–5.49 (m, 1H, H-7), 6.81 (dd, 1H, *J_14,16_* = 1.5, *J_14,4_* = 0.7, H-14), 7.03 (d, 1H, *J_16,14_* = 1.5, H-16). NMR ^13^C (150 MHz, CDCl_3_, δ, ppm): 33.73 (d, C-1), 76.18 (c, C-2), 71.49 (d, C-4), 42.10 (d, C-5), 132.46 (c, C-6), 123.93 (d, C-7), 27.49 and 27.62 (2t, C-8, C-9), 28.33 (q, C-10), 23.85 (q, C-11), 24.12 (q, C-12), 148.37 (c, C-13), 124.37 (d, C-14), 108.51 (s, C-15), 120.68 (d, C-16). HR-MS: *m*/*z* calcd. for C_15_H_19_OBrS: 326.0335. Found: 326.0334. ${\left[\alpha \right]}_{D}^{30}$ = 0 (c = 1.3, CHCl_3_).

#### 3.1.8. Reaction of 2-Carene-Containing Mixture and 5-bromothiophene-2-carbaldehyde **10h**

According to the GP, the reaction of the 2-carene-containing mixture and 5-bromothiophene-2-carbaldehyde **10h** gave (1*S*,3a*R*,7a*S*)-3,3,6-trimethyl-1-(5-bromothiophen-2-yl)-1,3,3a,4,5,7a-hexahydroisobenzofuran **11h** (0.176 g, 73%) and (1*R*,4*R*,5*R*)-2,2,6-trimethyl-4-(5-bromothiophen-2-yl)-3-oxabicyclo[3.3.1]non-6-ene **12h** (0.199 g, 15%).

**11h**: NMR ^1^H (600 MHz, CDCl_3_, δ, ppm, *J*/Hz): 1.26 (s, 3H, Me-9), 1.36 (s, 3H, Me-8), 1.47–1.56 (m, 1H, H_a_-4), 1.68 (br.s, 3H, Me-10), 1.72 (dm, 1H, ^2^*J* = 12.9, H_e_-4), 1.93–2.00 (m, 3H, 2H-5, H-3a), 2.75–2.81 (m, 1H, H-7a), 4.74 (d, 1H, *J_1,7a_* = 9.9, H-1), 5.24–5.27 (m, 1H, H-7), 6.69 (dd, 1H, *J_12,13_* = 3.7, *J_12,3a_* = 0.8, H-12), 6.86 (d, 1H, *J_13,12_* = 3.7, H-13). NMR ^13^C (150 MHz, CDCl_3_, δ, ppm): 81.56 (d, C-1), 83.42 (c, C-3), 46.27 (d, C-3a), 21.73 (t, C-4), 29.92 (t, C-5), 136.95 (c, C-6), 117.95 (d, C-7), 48.56 (d, C-7a), 30.83 (q, C-8), 23.92 (q, C-9), 23.49 (q, C-10), 148.65 (s, C-11), 124.00 (d, C-12), 129.22 (d, C-13), 110.99 (s, C-14). HR-MS: *m*/*z* calcd. for C_15_H_19_OBrS: 326.0335. Found: 326.0330. ${\left[\alpha \right]}_{D}^{30}$ = 34 (c = 1.2, CHCl_3_).

**12h**: NMR ^1^H (600 MHz, CDCl_3_, δ, ppm, *J*/Hz): 1.10–1.12 (m, 3H, Me-12), 1.27 (s, 3H, Me-10), 1.34 (s, 3H, Me-11), 1.50–1.54 (m, 1H, H-1), 1.69 (ddd, 1H, ^2^*J* = 12.5, *J_9an,1_* = *J_9an,5_* = 3.2, H-9an), 2.06 (dm, 1H, *J_8k,8n_* = 18.8, H-8k), 2.15–2.18 (m, 1H, H-5), 2.26 (dddd, 1H, ^2^*J* = 12.5, *J_9s,1_* = *J_9s,5_* = 3.2, *J_9s,8n_* = 1.1, H-9s), 2.35 (br.d, 1H, ^2^*J* = 18.8, H-8n), 5.03 (br.d, 1H, *J_4,5_* = 2.1, H-4), 5.47–5.50 (m, 1H, H-7), 6.63 (dd, 1H, *J_14,15_* = 3.7, *J_14,4_* = 0.8, H-14), 6.85 (d, 1H, *J_15,14_* = 3.7, H-15). NMR ^13^C (150 MHz, CDCl_3_, δ, ppm): 33.74 (d, C-1), 76.09 (c, C-2), 72.07 (d, C-4), 42.00 (d, C-5), 132.61 (c, C-6), 123.87 (d, C-7), 27.49 (t, C-8), 27.55 (t, C-9), 28.35 (q, C-10), 23.84 (q, C-11), 24.24 (q, C-12), 148.84 (c, C-13), 121.61 (d, C-14), 128.76 (d, C-15), 109.96 (s, C-16). HR-MS: *m*/*z* calcd. for C_15_H_19_OBrS: 326.0335. Found: 326.0331. ${\left[\alpha \right]}_{D}^{30}$ = 0 (c = 0.9, CHCl_3_).

#### 3.1.9. Reaction of 2-Carene-Containing Mixture and 5-nitrothiophene-2-carbaldehyde **10i**

According to the GP, the reaction of the 2-carene-containing mixture and 5-nitrothiophene-2-carbaldehyde **10i** gave a mixture of (1*R*,4*R*,5*R*)-2,2,6-trimethyl-4-(5-methylthiophen-2-yl)-3-oxabicyclo[3.3.1]non-6-ene **12i** and (1*R*,4*R*,5*R*)-2,2-dimethyl-6-methylene-4-(5-methylthiophen-2-yl)-3-oxabicyclo[3.3.1]nonane **13i** (0.221 g, 18%, 5.4:1).

The NMR spectra of **12i** and **13i** were recorded for their mixture (≈ 5.4:1).

**12i**: NMR ^1^H (600 MHz, CDCl_3_, δ, ppm, *J*/Hz): 1.03–1.05 (m, 3H, Me-12), 1.27 (s, 3H, Me-10), 1.338 (s, 3H, Me-11), 1.53–1.56 (m, 1H, H-1), 1.73 (ddd, 1H, ^2^*J* = 12.5, *J_9an,1_* = *J_9an,5_* = 3.2, H-9an), 2.07 (dm, 1H, ^2^*J* = 19.0, H-8k), 2.23–2.26 (m, 1H, H-5), 2.28 (dddd, 1H, ^2^*J* = 12.5, *J_9s,1_* = *J_9s,5_* = 3.2, *J_9s,8n_* = 1.2, H-9s), 2.35 (br.d, 1H, ^2^*J* = 19.0, H-8n), 5.06 (br.d, 1H, *J_4,5_* = 2.4, H-4), 5.48–5.51 (m, 1H, H-7), 6.84 (dd, 1H, *J_14,15_* = 4.2, *J_14,4_* = 1.0, H-14), 7.75 (d, 1H, *J_15,14_* = 4.2, H-15). NMR ^13^C (150 MHz, CDCl_3_, δ, ppm): 33.49 (d, C-1), 76.60 (c, C-2), 71.74 (d, C-4), 41.75 (d, C-5), 131.38 (c, C-6), 124.82 (d, C-7), 27.35 (t, C-8), 27.45 (t, C-9), 28.14 (q, C-10), 23.77 (q, C-11), 24.12 (q, C-12), 157.31 (c, C-13), 120.69 (d, C-14), 128.02 (d, C-15), 150.16 (br.s, C-16). HR-MS: *m*/*z* calcd. for C_15_H_19_O_3_NS: 293.1080. Found: 293.1076.

**13i**: NMR ^1^H (600 MHz, CDCl_3_, δ, ppm, *J*/Hz): 1.342 (s, 3H, Me-10), 1.37 (s, 3H, Me-11), 1.47–1.53 (m, 1H, H-8a), 1.76 (dm, 1H, ^2^*J* = 13.0, H-9s), 2.07–2.12 (m, 2H, H-7e, H-8e), 2.36–2.41 (m, 1H, H-9a), 2.43–2.46 (m, 1H, H-5), 2.67–2.75 (m, 1H, H-7a), 4.30 (dd, 1H, ^2^*J* = 2.4, *J_12,5_* = 2.4, H-12), 4.54 (dd, 1H, ^2^*J* = 2.4, *J_12__′,5_* = 2.4, H-12′), 5.16 (br.d, 1H, *J_4,5_* = 2.5, H-4), 6.73 (dd, 1H, *J_14,15_* = 4.2, *J_14,4_* = 1.0, H-14), 7.75 (d, 1H, *J_15,14_* = 4.2, H-15). The H-1 signal is overlapped by the H-1 signal of the main isomer **12i**. NMR ^13^C (150 MHz, CDCl_3_, δ, ppm): 34.76 (d, C-1), 76.71 (c, C-2), 73.30 (d, C-4), 45.30 (d, C-5), 146.62 (c, C-6), 30.73 (t, C-7), 28.46 (t, C-8), 31.39 (t, C-9), 27.53 (q, C-10), 24.23 (q, C-11), 110.58 (q, C-12), 156.23 (c, C-13), 121.10 (d, C-14), 128.22 (d, C-15), ≈150 (br.s, C-16). Broad singlet signal of C-16 could not be determined accurately due to low concentrations of **13i**. HR-MS: *m*/*z* calcd. for C_15_H_19_O_3_NS: 293.1080. Found: 293.1076.

#### 3.1.10. Reaction of 2-Carene-Containing Mixture and 5-nitrofuran-2-carbaldehyde **10j**

According to the GP, the reaction of the 2-carene-containing mixture and 5-nitrofuran-2-carbaldehyde **10j** gave (1*R*,4*R*,5*R*)-2,2,6-trimethyl-4-(5-nitrofuran-2-yl)-3-oxabicyclo[3.3.1]non-6-ene **12j** (0.215 g, 18%).

**12j**: NMR ^1^H (600 MHz, CDCl_3_, δ, ppm, *J*/Hz): 1.08–1.09 (m, 3H, all *J* < 2.5 Hz, Me-12), 1.28 (s, 3H, Me-10), 1.35 (s, 3H, Me-11), 1.54–1.58 (m, 1H, H-1), 1.75 (ddd, 1H, ^2^*J* = 12.6, *J_9an,1_* = *J_9an,5_* = 3.2, H-9an), 2.09 (dm, 1H, *J_8k,8n_* = 18.9, H-8k), 2.25 (dm, 1H, ^2^*J* = 12.6, H-9s), 2.34 (dm, 1H, ^2^*J* = 18.9, H-8n), 2.46–2.49 (m, 1H, H-5), 4.90 (br.d, 1H, *J_4,5_* = 2.2, H-4), 5.48–5.51 (m, 1H, H-7), 6.39 (dd, 1H, *J_14,15_* = 3.7, *J_14,4_* = 1.2, H-14), 7.24 (d, 1H, *J_15,14_* = 3.7, H-15). NMR ^13^C (150 MHz, CDCl_3_, δ, ppm): 33.75 (d, C-1), 76.38 (c, C-2), 69.63 (d, C-4), 38.69 (d, C-5), 131.49 (c, C-6), 124.56 (d, C-7), 27.47 (t, C-8), 27.00 (t, C-9), 28.23 (q, C-10), 23.65 (q, C-11), 23.07 (q, C-12), 160.14 (c, C-13), 108.81 (d, C-14), 112.97 (d, C-15), 150.96 (s, C-16). HR-MS: *m*/*z* calcd. for C_15_H_19_O_4_N: 277.1309. Found: 277.1306.

#### 3.1.11. Reaction of 2-Carene-Containing Mixture and thiophene-3-carbaldehyde **10k**

According to the GP, the reaction of the 2-carene-containing mixture and thiophene-3-carbaldehyde **10k** gave (1*S(R)*,3a*R*,7a*S*)-3,3,6-trimethyl-1-(thiophen-3-yl)-1,3,3a,4,5,7a-hexahydroisobenzofuran **11k** (0.165 g, 91%, *S*/*R* = 1.5:1) and (1*R*,4*R*,5*R*)-2,2,6-trimethyl-4-(thiophen-3-yl)-3-oxabicyclo[3.3.1]non-6-ene **12k** (0.213 g, 21%).

The NMR spectra of compound **11k** were recorded for the mixture (*S*)-**11k** and (*R*)-**11k** isomers (1.3:1).

(*S*)-**11k**: NMR ^1^H (500 MHz, CDCl_3_, δ, ppm, *J*/Hz): 1.28 (s, 3H, Me-9), 1.37 (s, 3H, Me-8), 1.53–1.61 (m, 1H, H_a_-4), 1.68 (br.s, 3H, Me-10), 1.69–1.76 (m, 1H, H_e_-4), 1.88–2.02 (m, 3H, 2H-5, H-3a), 2.73–2.80 (m, 1H, H-7a), 4.68 (d, 1H, *J_1,7a_* = 10.1, H-1), 5.22–5.25 (m, 1H, H-7), 7.09 (dd, 1H, *J_12,13_* = 5.0, *J_12,14_* = 1.3, H-12), 7.18 (ddd, 1H, *J_14,13_* = 3.0, *J_14,13_* = 1.3, *J_14,1_* = 0.7, H-14), 7.22 (dd, 1H, *J_13,12_* = 5.0, *J_13,14_* = 3.0, H-13). NMR ^13^C (125 MHz, CDCl_3_, δ, ppm): 81.53 (d, C-1), 82.61 (c, C-3), 46.20 (d, C-3a), 21.82 (t, C-4), 29.99 (t, C-5), 136.40 (c, C-6), 118.65 (d, C-7), 47.37 (d, C-7a), 30.95 (q, C-8), 24.02 (q, C-9), 23.53 (q, C-10), 143.91 (s, C-11), 125.64 (d, C-12), 125.68 (d, C-13), 120.75 (d, C-14). HR-MS: *m*/*z* calcd. for C_15_H_20_OS: 248.1229. Found: 248.1230.

(*R*)-**11k**: NMR ^1^H (500 MHz, CDCl_3_, δ, ppm, *J*/Hz): 1.30 (s, 3H, Me-8), 1.33 (s, 3H, Me-9), 1.50 (br.s, 3H, Me-10), 1.69–1.76 (m, 1H, H_e_-4), 1.81–1.85 (m, 2H, 2H-5), 1.89–1.97 (m, 1H, H-3a), 3.17–3.24 (m, 1H, H-7a), 4.89–4.93 (m, 1H, H-7), 5.22 (d, 1H, *J_1,7a_* = 9.5, H-1), 6.89 (dd, 1H, *J_12,13_* = 5.0, *J_12,14_* = 1.2, H-12), 7.08 (dm, 1H, *J_14,13_* = 3.0, H-14), 7.17 (dd, 1H, *J_13,12_* = 5.0, *J_13,14_* = 3.0, H-13). NMR ^13^C (125 MHz, CDCl_3_, δ, ppm): 77.91 (d, C-1), 81.72 (c, C-3), 44.94 (d, C-3a), 22.45 (t, C-4), 28.70 (t, C-5), 134.35 (c, C-6), 119.72 (d, C-7), 42.83 (d, C-7a), 26.98 (q, C-8), 23.76 (q, C-9), 23.49 (q, C-10), 142.92 (s, C-11), 126.92 (d, C-12), 124.49 (d, C-13), 121.07 (d, C-14). HR-MS: *m*/*z* calcd. for C_15_H_20_OS: 248.1229. Found: 248.1230.

**12k**: NMR ^1^H (500 MHz, CDCl_3_, δ, ppm, *J*/Hz): 0.95–0.98 (ddd, 3H, *J_12,7_* = 2.4, *J_12,8k_* = 2.1, *J_12,8n_* = 1.8, Me-12), 1.29 (s, 3H, Me-10), 1.36 (s, 3H, Me-11), 1.52–1.55 (m, 1H, H-1), 1.71 (ddd, 1H, ^2^*J* = 12.5, *J_9an,1_* = *J_9an,5_* = 3.2, H-9an), 2.07 (dm, 1H, *J_8k,8n_* = 18.8, H-8k), 2.17–2.19 (m, 1H, H-5), 2.30 (dddd, 1H, ^2^*J* = 12.5, *J_9s,1_* = *J_9s,5_* = 3.2, *J_9s,8n_* = 1.2, H-9s), 2.37 (br.d, 1H, ^2^*J* = 18.8, H-8n), 4.95 (d, 1H, *J_4,5_* = 2.1, H-4), 5.42–5.45 (m, 1H, H-7), 6.98 (dd, 1H, *J_14,15_* = 5.0, *J_14,16_* = 1.2, H-14), 7.09 (ddd, 1H, *J_16,15_* = 3.0, *J_16,14_* = 1.2, *J_16,4_* = 0.8, H-16), 7.19 (dd, 1H, *J_15,14_* = 5.0, *J_15,16_* = 3.0, H-15). NMR ^13^C (125 MHz, CDCl_3_, δ, ppm): 33.97 (d, C-1), 75.31 (c, C-2), 72.06 (d, C-4), 40.97 (d, C-5), 133.23 (c, C-6), 123.14 (d, C-7), 27.61 (t, C-8), 27.92 (t, C-9), 28.58 (q, C-10), 23.87 (q, C-11), 23.68 (q, C-12), 144.27 (c, C-13), 125.62 (d, C-14), 119.47 (d, C-15), 124.69 (d, C-16). HR-MS: *m*/*z* calcd. for C_15_H_20_OS: 248.1229. Found: 248.1230. ${\left[\alpha \right]}_{D}^{30}$ = 0 (c = 1.8, CHCl_3_).

#### 3.1.12. Reaction of Limonene and 2-thiophenecarbaldehyde **10d**

To the suspension of the K10 (1.5 g) in methylene chloride (15 mL), a solution of 0.40 g aldehyde in methylene chloride (5 mL) and 0.50 g of limonene was added. The solvent was distilled off, and the reaction mixture was kept at room temperature for 2 h. Thereafter, ethyl acetate was added, the catalyst was filtered off, and the filtrate was evaporated. Isolation of the reaction products was carried out by column chromatography to give **11d** and **12d** (0.582 g, 64%, 14:1).

### 3.2. Real-Time Detection of TDP1 Activity

A fluorophore quencher-coupled oligonucleotide biosensor was used for TDP1 enzyme activity real-time fluorescence detection [38]. The biosensor 5′-FAM-AAC GTC AGG GTC TTC C- BHQ1-3′ is a 16-mer single-stranded oligonucleotide with a 5′- fluorophore (FAM), and a 3′-quencher (BHQ1). Recombinant protein TDP1 was expressed in *Escherichia coli* (pET 16B plasmid containing TDP1 cDNA was provided by Dr. K.W. Caldecott, University of Sussex, United Kingdom) and isolated as described by Lebedeva et al. [43]. The reaction mixture contained TDP1 reaction buffer (50 mM Tris-HCl, 50 mM NaCl, 7 mM β-mercaptoethanol), 50 nM oligonucleotide substrate, varied concentrations of the tested compounds, and 1.5 nM purified TDP1 in a final volume of 200 μL.

The TDP1 reaction mixture was incubated at rt in a POLARstar OPTIMA fluorimeter (BMG LABTECH, GmbH). Fluorescence intensity was measured every 1 min (Ex485/Em520 nm). The efficiency of TDP1 inhibition was evaluated by comparing the rate of increase in the fluorescence of the biosensor in the presence of the compound to that of DMSO (1.5%) control wells. IC_50_ values were determined using an 11-point concentration–response curve. The data were imported into MARS Data Analysis 2.0 program (BMG LABTECH) and the slope during the linear phase was calculated. The IC_50_ measurements were carried out in at least three independent experiments.

### 3.3. TDP1 Activity by Gel-Based Assay

A fluorophore-labeled oligonucleotide 5′- FAM-AAC GTC AGG GTC TTC C-tyrosine-3′ was used as a standard gel-based method for the indication of TDP1 enzyme activity. The oligonucleotide containing a natural phosphotyrosine adduct at the 3′-end is a substrate for TDP1 [33]. The reaction mixture contained reaction buffer (50 mM Tris-HCl, 50 mM NaCl, 7 mM β-mercaptoethanol), 50 nM oligonucleotide substrate, 5 nM purified TDP1, or 1 μg of cell extract in a final volume of 20 μL. The reaction was conducted at 37 °C for 20 min. The reaction products were separated by electrophoresis in a 20% denaturing polyacrylamide gel with 7 M urea. A Typhoon FLA 9500 phosphorimager (GE Healthcare) was used for gel scanning and imaging, and the data were analyzed with QuantityOne 4.6.7 software.

### 3.4. Obtainment of TDP1 Knockout HEK293FT Clones

#### 3.4.1. Plasmid Construction for Human TDP1 Gene Knockout

The sgRNAs design was performed using the Benchling CRISPR tool (https://www.benchling.com/). Two protospacers (PAM sequences in brackets) were selected for the DNA sequence deletion in the first protein-coding exon of the human *TDP1* gene exon 3 (third exon in mRNA, NM_001008744.2): TDP1-gRNA1 AGACGAGTATGAGACATCAG(GGG) and TDP1-gRNA2 GCAGAAAAGCGGTTCCCAGG(AGG). Corresponding oligonucleotides were cloned in plasmid pSpCas9(BB)-2A-GFP (PX458) (the plasmid was a gift from Feng Zhang (Addgene plasmid #48138; http://n2t.net/addgene:48138; RRID:Addgene_48138)) as previously described [44]. Transfection-grade plasmid DNA was isolated using the PureLink HiPure Plasmid Miniprep Kit (ThermoFisher Scientific, Waltham, MA, USA).

#### 3.4.2. Knockout HEK293FT Clone Generation

First, 5 × 10^5^ HEK293FT cells were plated into a well of a 12-well plate and cotransfected with the constructed plasmids TDP1-gRNA1 and TDP1-gRNA2 (0.25 µg of each) using Lipofectamine 3000 Reagent (Thermofisher Scientific). Growth medium contained DMEM/F12 (Gibco, Co Dublin, Ireland) 1:1, 10% fetal bovine serum (FBS) (Gibco), 100 U/mL penicillin-streptomycin (Gibco), and 1x GlutaMAX (Gibco). Then, 48 h after transfection, cells were detached using TrypLE Express (TrypLE, Gibco), and the GFP-positive cell population was enriched by cell sorting using a BD FACSAria III Cell Sorter (BD Biosciences, East Rutherford, NJ, USA). Transfected cells were plated onto a 96-well plate, one cell per well. Single-cell clones grew for two weeks before they were replicated to another 96-well plate, so we obtained two equal 96-well plates with cell clones: One plate was used for PCR analysis of the deletion in the *TDP1* gene while the other plate was used for the mutant cell clone multiplicatioin.

#### 3.4.3. Analysis of CRISPR/Cas9-Mediated Deletions in the TDP1 Gene

Genome DNA was extracted from cells on one of two 96-well plates using 50 μL of QuickExtract™ DNA Extraction Solution (Epicentre) per well. The DNA extracts were diluted with 200 μL of mQ water. Two microliters of diluted DNA extract were used for PCR amplification of the target region with the primers: TDP1-scF 5′-TCAGGAAGGCGATTATGGGAG-3′ and TDP1-scR 5′-TTGATGTGGAGGGCTCCAG-3′. Reactions were run on a S1000 Thermal Cycler (Bio-Rad, Hercules, CA ,USA) using BioMaster HS-Taq PCR-Color (2×) (Biolabmix, Novosibirsk Oblast, Russia) with the following program: 95 °C 3 min; 35 cycles: 95 °C 30 s, 68 °C 30 s, 72 °C 10 s; 72 °C 3 min. Products of the reactions were resolved in 1.5% agarose gel stained with ethidium bromide. PCR products with a lower molecular weight than the wild type were selected for subsequent analysis. PCR products were gel purified and cloned in the pGEM-T Easy vector (Promega, Madison, WI, USA). Ten independent plasmid clones were sequenced using M13 universal primers. Products of Sanger sequencing reactions were analyzed using the ABI 3130xl Genetic Analyzer (SB RAS Genomics Core Facility, http://www.niboch.nsc.ru/doku.php/corefacility). Three clones containing deletions in the *TDP1* gene were found: Clone B2 Δ197bp/Δ16bp+InT; clone B5 Δ198bp/Δ196bp; and clone E3 Δ197bp/Δ200bp. To study the expression of the mutant form of TDP1 in clone E3, total RNA was isolated from cells using TRIZOL reagent as per the manufacturer’s protocols. The synthesis of the first cDNA strand was performed using SuperScript ™ III Reverse Transcriptase (ThermoFisher Scientific) according to the manufacturer’s recommendations. To control DNA contamination of the RNA sample, a reaction was performed without the addition of reverse transcriptase (RT-).

### 3.5. Cell Culture Assay

The cytotoxicity of the compounds was examined against human cell lines HEK293FT (human embryonic kidney), WT and TDP1-deficient (TDP1-/-), HCT116 (human colon cancer), IMR-32 (human neuroblastoma cell line), HeLa (cervical cancer), and Krebs-2 carcinoma ascites using MTT and EZ4U colorimetric tests or an xCELLigence DP Real-Time Cell Analyzer (ACEA Biosciences, San Diego, CA, USA) for HEK293FT in cells exposed to the compounds. The cells were grown in DMEM/F12 or IMDM (for IMR-32 and Krebs-2) medium (Gibco), with 1x GlutaMAX (Gibco), 50 IU/mL penicillin, 50 μg/mL streptomycin (MP Biomedicals, Irvine, CA, USA) and in the presence of 10% fetal bovine serum (Biolot). The cells (~20,000 cells per well) were incubated for 24 h at 37 °C in 5% CO_2_, and then treated with the tested compounds (the volume of the added reagents was 1/100 of the total volume of the culture medium, the amount of DMSO was 1% of the final volume as indicated). Control cells were grown in the presence of 1% DMSO where indicated. After 72 h of cell incubation, the relative amount of living cells was determined using a colorimetric test of the amount of formazan converted from 3-(4,5-dimethylthiazol-2-yl)-2,5-diphenyl-2H-tetrazolium bromide (MTT test) or EZ4U Cell Proliferation and Cytotoxicity Assay (Biomedica, Austria), as per the manufacturer’s protocols. The real-time detection of the living cells was done by an xCELLigence DP Real-Time Cell Analyzer (ACEA Biosciences). The measurements were carried out in three parallel experiments.

### 3.6. Calculation of Molecular Descriptors

The QikProp 3.2 [45] software was used to calculate the molecular descriptors of the ligands. The reliability of QikProp is established for the calculated descriptors [46]. The known drug indexes (KDI) were calculated from the molecular descriptors as described by Eurtivong and Reynisson [42]. For application in Excel, columns for each property were created and the following equations used do derive the KDI numbers for each descriptor: KDI MW: =EXP(-((*MW*-371.76)^2^)/((2 × 112.76)^2^)), KDI Log P: =EXP(-((*LogP*-2.82)^2^)/((2 × 2.21)^2^)), KDI HD: =EXP(-((*HD*-1.88)^2^)/((2 × 1.7)^2^)), KDI HA: =EXP(-((*HA*-5.72)^2^)/((2 × 2.86)^2^)), KDI RB- =EXP(-((*RB*-4.44)^2^)/((2 × 3.55)^2^)), and KDI PSA: =EXP(-((*PSA*-79.4)^2^)/((2 × 54.16)^2^)). These equations could simply be copied into Excel and the descriptor name (e.g., *MW*) substituted with the value in the relevant column. In order to derive KDI_2A_, this equation was used: = (KDI MW + KDI LogP + KDI HD + KDI HA + KDI RB + KDI PSA) and for KDI_2B_: = (KDI MW × KDI LogP × KDI HD × KDI HA × KDI RB × KDI PSA).

## 4. Conclusions

A new class of effective TDP1 inhibitors was discovered with activity in the low micromolar to nanomolar concentrations. All the tested derivatives exhibited low intrinsic cytotoxicity when tested in a panel of cancer cell lines. The most promising compounds with the best combination of TDP1 inhibition, low toxicity, and enhancement of Tpc efficacy were compounds **11h** and **12k**, with IC_50_ values of 0.75 and 1.20 μM, respectively. Thus, these new inhibitors are promising candidates for adjuvant therapy, mainly due to the absence of an additional toxic load.

Furthermore, a panel of isogenic clones of the HEK293FT cell line knockout for TDP1 was created using the CRISPR-Cas9 system. Clone E3 with the Δ197bp/Δ200bp deletion in the TDP1 gene was selected for the experiments. We investigated the cytotoxic effect of the Tpc and the TDP1 inhibitors **11h** and **12k** separately and jointly. HEK293FT TDP1-/- cells were more sensitive to Tpc compared to WT cells. For both **11h** and **12k**, we observed cell growth suppression in the presence of Tpc only for WT cells but not for the TDP1 knockout cells. It can therefore be stated that the synergistic effect with Tpc on HEK293FT cell growth is only caused by TDP1 activity inhibition by **11h** and **12k**, with minimal off-target effects. These results make a significant contribution to our understanding of the mechanism of action for the non-cytotoxic 3-carene-derived TDP1 inhibitors. In conclusion, we demonstrated the existence of TDP1 inhibitors, which do not modulate other biomolecular targets, which in turn makes the prospect of developing viable drug candidates more realistic since off-target toxicity often leads to undesirable side effects [47].

## Figures and Tables

**Table 1:** Results of reaction of 2-carene-containing mixture with aldehides **10a**, **c**–**k**.

R	RCHO	Product 11 (Yield, (*S*)-/(*R*) Ratio)	Products 12 and 13 (Total Yield, %, 12/13)
4-Hydroxy-3-methoxyphenyl	**10a**	**11a** (81%, (*S*)-/(*R*) = 1:1)	**12a** (11%)
(*E*)-prop-1-en-1-yl	**10c**	**11c** (89%, (*S*)-/(*R*) = 1.3:1)	
Thiophen-2-yl	**10d**	**11d** (78%, (*S*)-isomer)	**12d**, **13d** (5%, 7:1)
3-Methylthiophen-2-yl	**10e**	**11e** (86%, (*S*)-isomer)	**12e** (2%)
5-Methylthiophen-2-yl	**10f**	**11f** (85%, (*S*)-isomer)	**12f**, **13f** (2%, 3:1)
4-Bromothiophen-2-yl	**10g**	**-**	**12g** (20%)
5-Bromothiophen-2-yl	**10h**	**11h** (73%, (*S*)-isomer)	**12h** (15%)
5-Nitrothiophen-2-yl	**10i**	**-**	**12i**, **13i** (18%, 5:1)
5-Nitrofuran-2-yl	**10j**	**-**	**12j** (18%)
Thiophen-3-yl	**10k**	**11k** (91%, (*S*)-/(*R*) = 1.5:1)	**12k** (21%)

**Table 2:** TDP1 inhibition potency of the 3-carene derivatives.

R	Compound 11	IC_50_^1^, μM	Compound 12	IC_50_^1^, μM
4-Hydroxy-3-methoxyphenyl	**11a**	>20	**12a**	>20
(*E*)-prop-1-en-1-yl	**11c**	>20	-	-
Thiophen-2-yl	**11d**	4.85 ± 1.06	**12d**	3.35 ± 1.06
3-Methylthiophen-2-yl	**11e**	3.6 ± 1.7	**12e**	2.25 ± 0.63
5-Methylthiophen-2-yl	**11f**	4.7 ± 2.0	**-**	-
4-Bromothiophen-2-yl	**-**	-	**12g**	0.65 ± 0.22
5-Bromothiophen-2-yl	**11h**	0.75 ± 0.07	**12h**	1.75 ± 0.78
5-Nitrothiophen-2-yl	**-**	-	**12i**	14 ± 1
5-Nitrofuran-2-yl	-	-	**12j**	28 ± 10
Thiophen-3-yl	**11k**	1.60 ± 0.56	**12k**	1.20 ± 0.14
**Furamidine**		1.2 ± 0.3		

^1^ IC_50_—half maximal inhibitory concentration.

## Supplementary Materials

Supplementary Materials can be found online.
