# Supplementary material for: Design, Synthesis, and Biological Investigation of Novel Classes of 3-Carene-Derived Potent Inhibitors of TDP1
Source: Molecules. 2020 Jul 31;25(15):3496. doi: 10.3390/molecules25153496 (PMC7436013; doi:10.3390/molecules25153496)
Supplement: Supplementary file 1 [file molecules-25-03496-s001.pdf]

Supplementary Information for

# Design, synthesis and biological investigation of novel classes of 3-carene-derived potent inhibitors of TDP1

**Irina V. Il'ina[1], Nadezhda S. Dyrkheeva[2], Alexandra L. Zakharenko[2], Alexander Yu. Sidorenko[3], Nikolay S. Li-Zhulanov[1,4], Dina V. Korchagina[1], Raina Chand[5], Daniel M. Ayine-Tora[5], Arina A. Chepanova[2], Olga D. Zakharova[2], Ekaterina S. Ilina[2], Jóhannes Reynisson[6], Anastasia A. Malakhova[2,7], Sergey P. Medvedev[7,8], Suren M. Zakian[4,7], Konstantin P. Volcho[1,4,*], Nariman F. Salakhutdinov[1,4] and Olga I. Lavrik[2,4]**

[1] N.N. Vorozhtsov Novosibirsk Institute of Organic Chemistry, Siberian Branch of Russian Academy of Sciences, 9, Lavrentiev Ave., Novosibirsk 630090, Russia
[2] Institute of Chemical Biology and Fundamental Medicine, Siberian Branch of Russian Academy of Sciences, 8, Lavrentiev Ave., Novosibirsk 630090, Russia
[3] Institute of Chemistry of New Materials of National Academy of Sciences of Belarus, 220141, Skaryna Str., 36, Minsk, Belarus
[4] Novosibirsk State University, 2, Pirogova Str., Novosibirsk 630090, Russia
[5] School of Chemical Sciences, The University of Auckland, Private Bag 92019, Victoria Street West, Auckland 1142, New Zealand
[6] School of Pharmacy and Bioengineering, Keele University, Hornbeam Building, Staffordshire, ST5 5BG, United Kingdom
[7] Federal Research Centre Institute of Cytology and Genetics of the Siberian Branch of Russian Academy of Sciences, 10, Lavrentiev Ave, Novosibirsk 630090, Russia
[8] E.Meshalkin National medical research center of the Ministry of Health of the Russian Federation, 15 Rechkunovskaya Str., Novosibirsk 630055, Russia
* Correspondence: volcho@nioch.nsc.ru; Tel.: +44-(0)-178-2733-985

**Content:**


1. Scheme S1 2
2. Figure S1 3
3. Figure S2 4
4. Tables S1-S4 5
5. NMR spectra of the products 8
6. Molecular modelling 21
7. Optimized Cartesian coordinates of the ligands 23

References 43

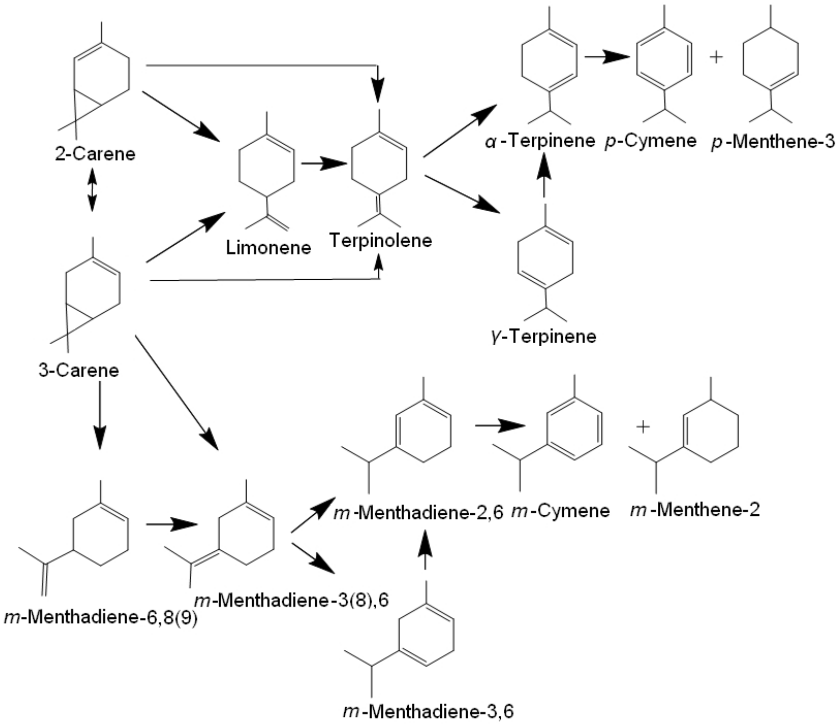


**Scheme S1.** A reaction pathways for 3-carene isomerization [1].

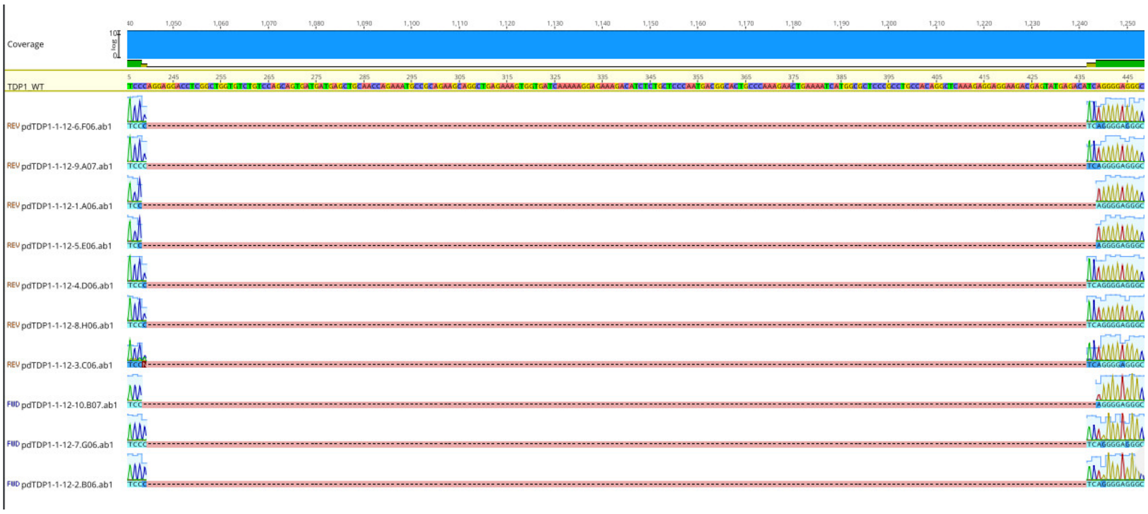

**a)** Clone E3 Δ197bp/Δ200bp.

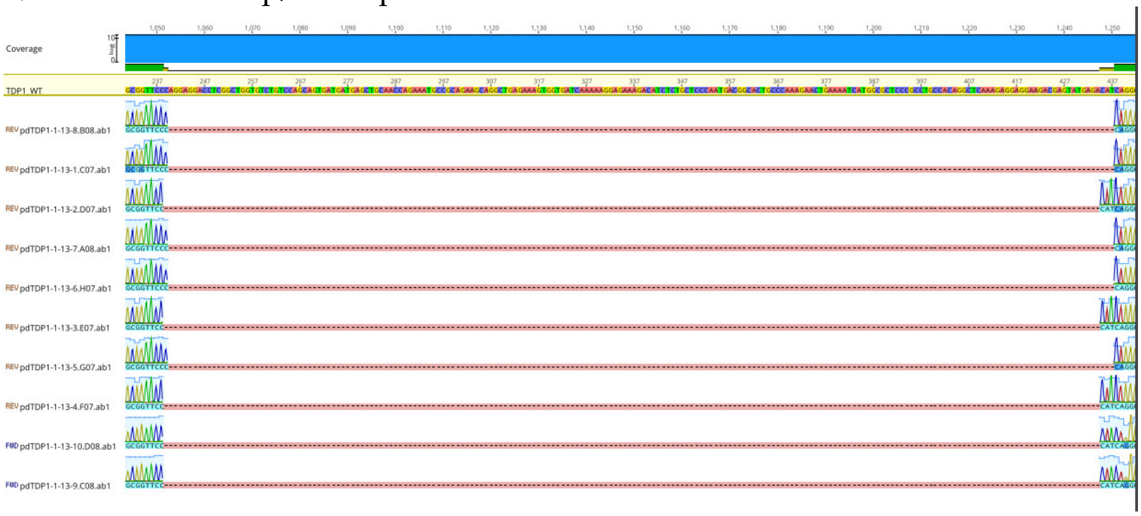

**b)** Clone B5 Δ 198bp/Δ196bp.

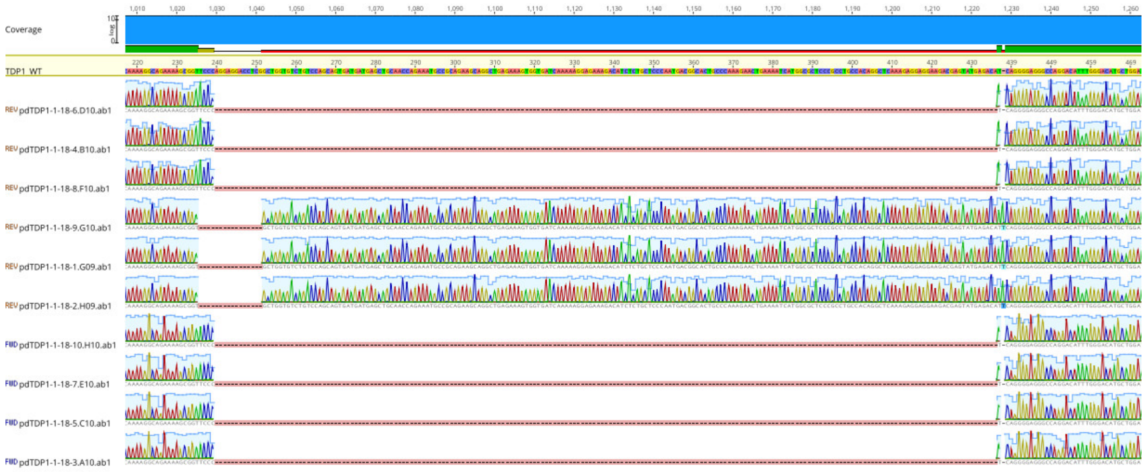

**c)** Clone B2 Δ197bp/Δ16bp+InT.

**Figure S1.** Verification of deletion in the TDP1 gene by genome DNA sequencing. Alignment of plasmid clones sequenograms (ten plasmid clones for each cell clone B2, B5 and E3) with the wild-type sequence of the TDP1 gene revealed the presence of deletions and insertions that shift the reading frame and potentially disrupt the synthesis of the corresponding protein.

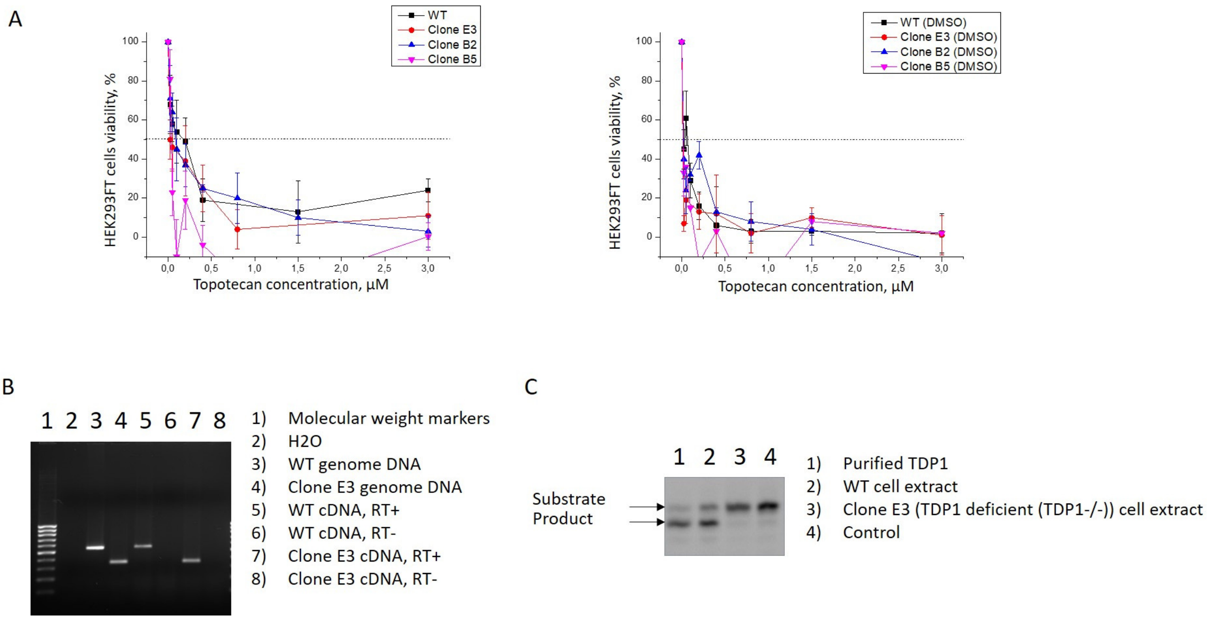


**Figure S2**. A. Topotecan cytotoxicity on HEK293FT WT and TDP1 -/- cells, dose-dependent action of topotecan by colorimetric test. B. PCR-analysis of the TDP1 allele length and expression. PCR analysis showed the presence of transcription of only alleles containing a deletion in the first protein encoding exon (third exon in mRNA, NM_001008744.2) of the TDP1 gene in clone E3 (line 7). (RT +) - cDNA synthesis reaction with the addition of reverse transcriptase; (RT-) - cDNA synthesis reaction without the addition of reverse transcriptase. C. Identification of TDP1 3′-phosphotyrosyl cleavage activity in the HEK293FT cell extracts: WT (lane 2) and clone E3 cells (lane 3). There was no established cleavage activity in the clone E3 cell extract in contrast to control WT cell extract and purified TDP1.

**Table S1.** The scoring results of the ligands for the four scoring functions used. GoldScore (GS), ChemScore(CS), ChemPLP (Piecewise Linear Potential) and ASP(Astex Statistical Potential).

| | COMPOUND ID | ASP | CS | GS | PLP |
|---|---|---|---|---|---|
| 1 | **11a** | 29.2 | 25.0 | 51.9 | 51.6 |
| 2 | **12a** | 27.2 | 27.5 | 47.2 | 53.0 |
| 3 | **11c** | 19.9 | 21.9 | 38.1 | 50.3 |
| 4 | **11d** | 22.1 | 22.7 | 45.7 | 51.1 |
| 5 | **12d** | 21.4 | 20.9 | 42.6 | 44.5 |
| 6 | **11e** | 23.0 | 23.2 | 43.8 | 52.7 |
| 7 | **12e** | 22.1 | 22.0 | 39.6 | 44.3 |
| 8 | **11f** | 22.4 | 23.7 | 44.5 | 55.8 |
| 9 | **12g** | 20.3 | 19.7 | 47.7 | 42.5 |
| 10 | **11h** | 22.1 | 22.9 | 46.9 | 50.5 |
| 11 | **12h** | 21.6 | 22.3 | 49.4 | 42.8 |
| 12 | **12i** | 24.7 | 23.6 | 41.8 | 47.2 |
| 13 | **12j** | 25.8 | 23.6 | 41.0 | 50.0 |
| 14 | **11k** | 22.0 | 22.7 | 47.4 | 54.5 |
| 15 | **12k** | 21.6 | 21.9 | 43.2 | 47.2 |

**Table S2.** Molecular Descriptor values of the ligands as calculated by QikProp.

| | **Compound** | **MW** | **HB DONOR** | **HB ACCEPTOR** | **Log P** | **PSA** | **Rot. Bonds** |
|---|---|---|---|---|---|---|---|
| 1. | **11a** | 288.4 | 1 | 2.25 | 4.2 | 36.9 | 2 |
| 2. | **12a** | 288.4 | 1 | 2.25 | 4.1 | 35.1 | 2 |
| 3. | **11c** | 206.3 | 0 | 0.75 | 3.7 | 8.0 | 1 |
| 4. | **11d** | 248.4 | 0 | 0.75 | 4.3 | 8.0 | 0 |
| 5. | **12d** | 248.3 | 0 | 0.75 | 4.2 | 6.1 | 0 |
| 6. | **11e** | 262.4 | 0 | 0.75 | 4.4 | 7.9 | 0 |
| 7. | **12e** | 262.4 | 0 | 0.75 | 4.3 | 6.1 | 0 |
| 8. | **11f** | 262.4 | 0 | 0.75 | 4.5 | 8.0 | 0 |
| 9. | **12g** | 248.4 | 0 | 0.75 | 4.2 | 5.8 | 0 |
| 10. | **11h** | 327.2 | 0 | 0.75 | 5.1 | 8.0 | 0 |
| 11. | **12h** | 327.3 | 0 | 0.75 | 5.0 | 6.1 | 0 |
| 12. | **12i** | 293.4 | 0 | 1.75 | 4.1 | 52.4 | 1 |
| 13. | **12j** | 277.3 | 0 | 2.25 | 3.3 | 60.0 | 1 |
| 14. | **11k** | 327.3 | 0 | 0.75 | 5.1 | 6.1 | 0 |
| 15. | **12k** | 248.4 | 0 | 0.75 | 4.4 | 7.5 | 0 |

**Table S3.** Definition of lead-like, drug-like and Known drug space (KDS) in terms of molecular descriptors. The values given are the maxima for each descriptor for the volumes of chemical space used.

| | **Lead-like Space** | **Drug-like Space** | **Known Drug Space** |
|---|---|---|---|
| Molecular weight (g $mol^{-1}$) | 300 | 500 | 800 |
| Lipophilicity (Log P) | 3 | 5 | 6.5 |
| Hydrogen bond donors (HD) | 3 | 5 | 7 |
| Hydrogen bond acceptors (HA) | 3 | 10 | 15 |
| Polar surface area ($Å^2$) (PSA) | 60 | 140 | 180 |
| Rotatable bonds (RB) | 3 | 10 | 17 |

**Table S4.** KDI values.

| | Compound | $KDI_{2A}$ | $KDI_{2B}$ |
|---|---|---|---|
| 1. | **11a** | 5.15 | 0.39 |
| 2. | **12a** | 4.19 | 0.10 |
| 3. | **11c** | 5.15 | 0.39 |
| 4. | **11d** | 4.17 | 0.10 |
| 5. | **12d** | 4.16 | 0.10 |
| 6. | **11e** | 4.20 | 0.11 |
| 7. | **12e** | 4.20 | 0.10 |
| 8. | **11f** | 4.19 | 0.10 |
| 9. | **12g** | 4.25 | 0.11 |
| 10. | **11h** | 4.26 | 0.11 |
| 11. | **12h** | 4.16 | 0.10 |
| 12. | **12i** | 4.89 | 0.28 |
| 13. | **12j** | 5.02 | 0.32 |
| 14. | **11k** | 4.25 | 0.11 |
| 15. | **12k** | 4.15 | 0.10 |

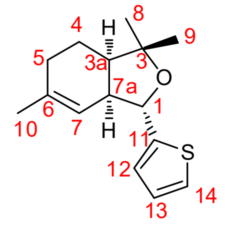

H
8
9
4
3a
3
5
7a
O
6
1
10
7
H
11
S
12
14
13


**11d**

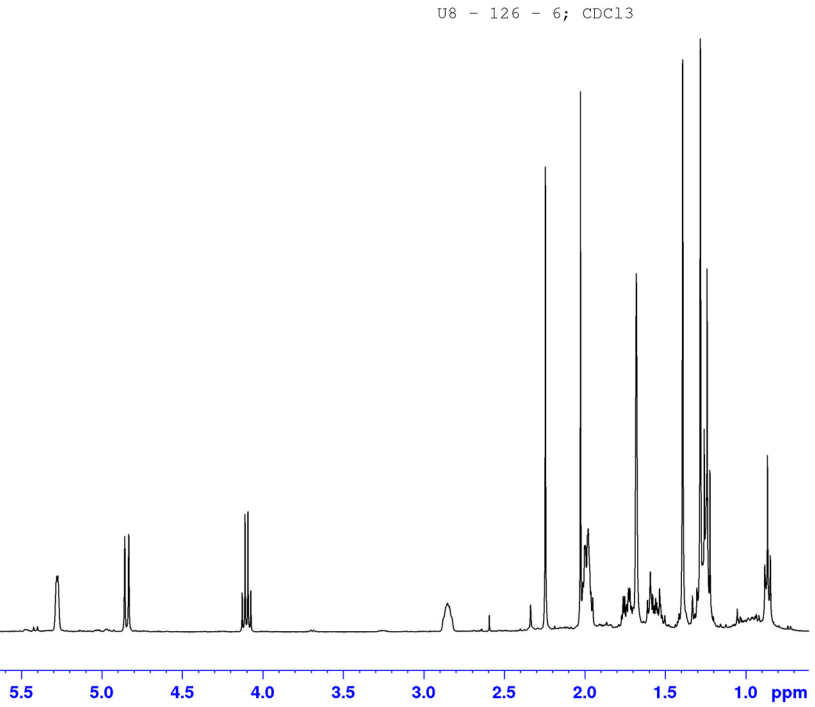

U8 - 126 - 6; CDCl3
7.0
6.5
6.0
5.5
5.0
4.5
4.0
3.5
3.0
2.5
2.0
1.5
1.0
ppm


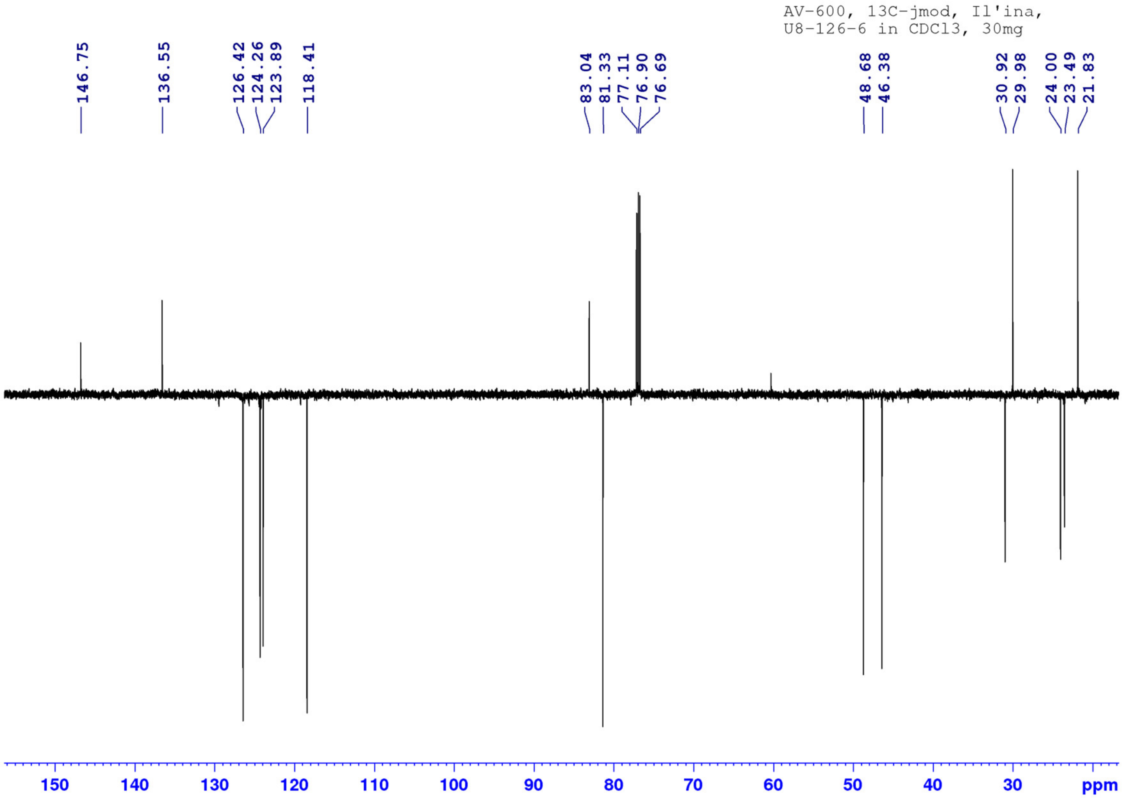

AV-600, 13C-jmod, Il'ina,
U8-126-6 in CDCl3, 30mg
146.75
136.55
126.42
124.26
123.89
118.41
83.04
81.33
77.11
76.90
76.69
48.68
46.38
30.92
29.98
24.00
23.49
21.83
150
140
130
120
110
100
90
80
70
60
50
40
30
ppm

The NMR spectra of **12d** and **13d** were recorded for their mixture (≈ 7:1).

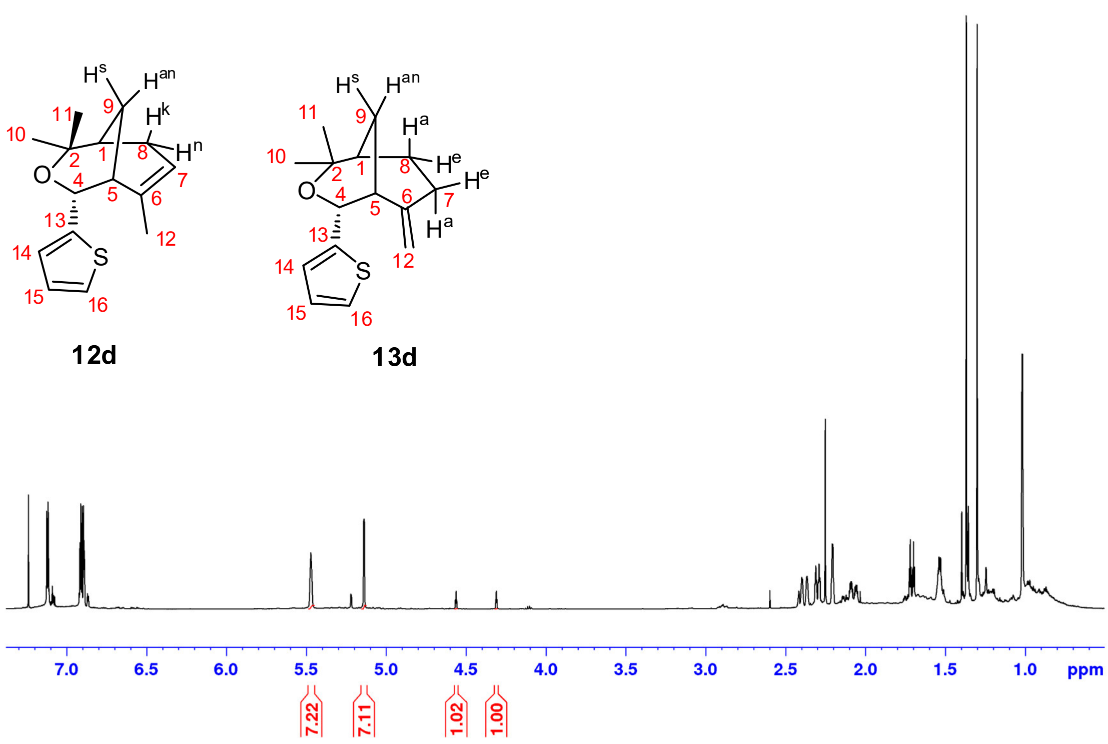


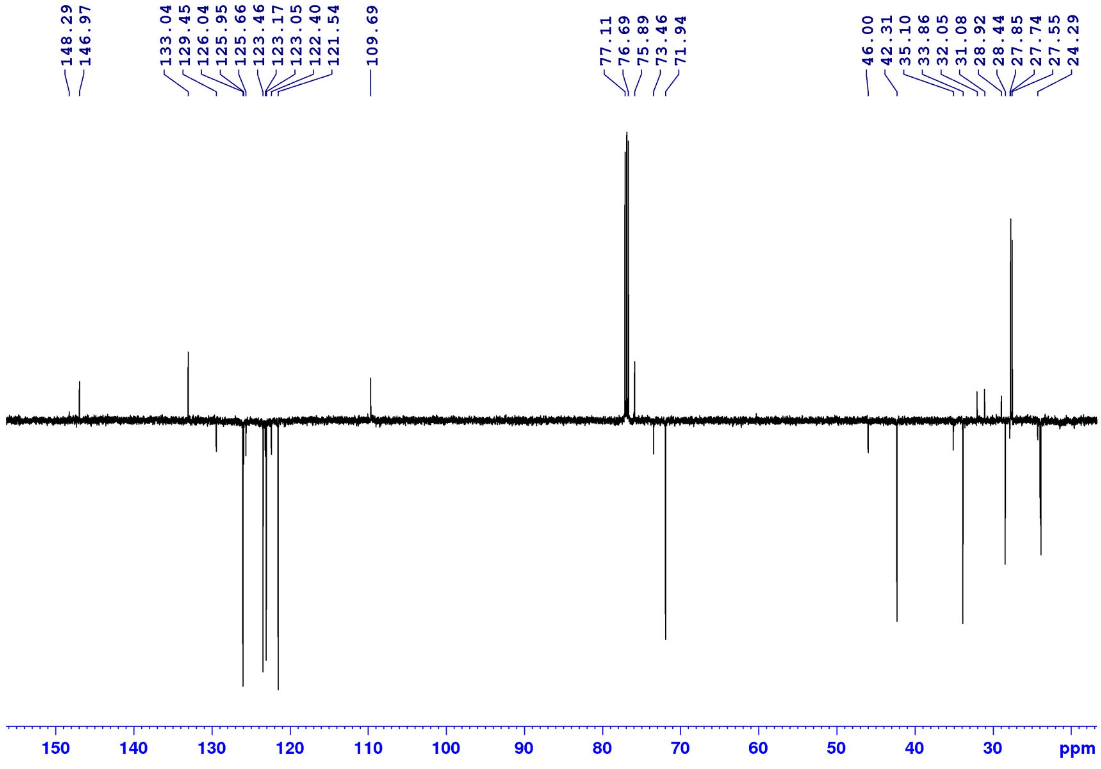

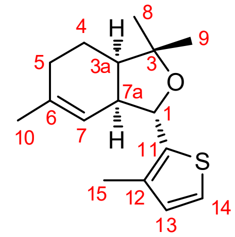

4
H
8
9
5
3a
3
O
7a
1
6
10
7
H
11
S
15
12
14
13


**11e**

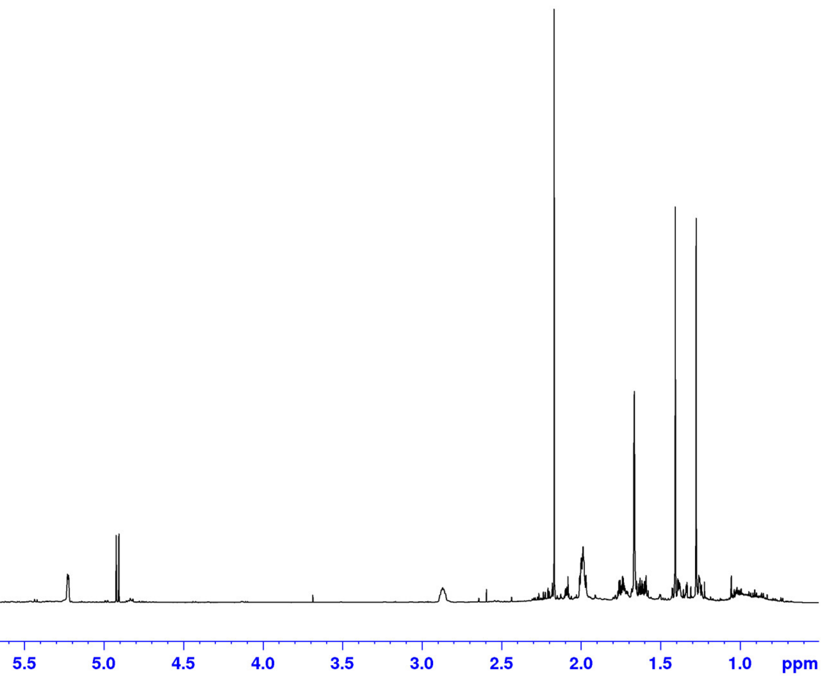

7.0
6.5
6.0
5.5
5.0
4.5
4.0
3.5
3.0
2.5
2.0
1.5
1.0
ppm


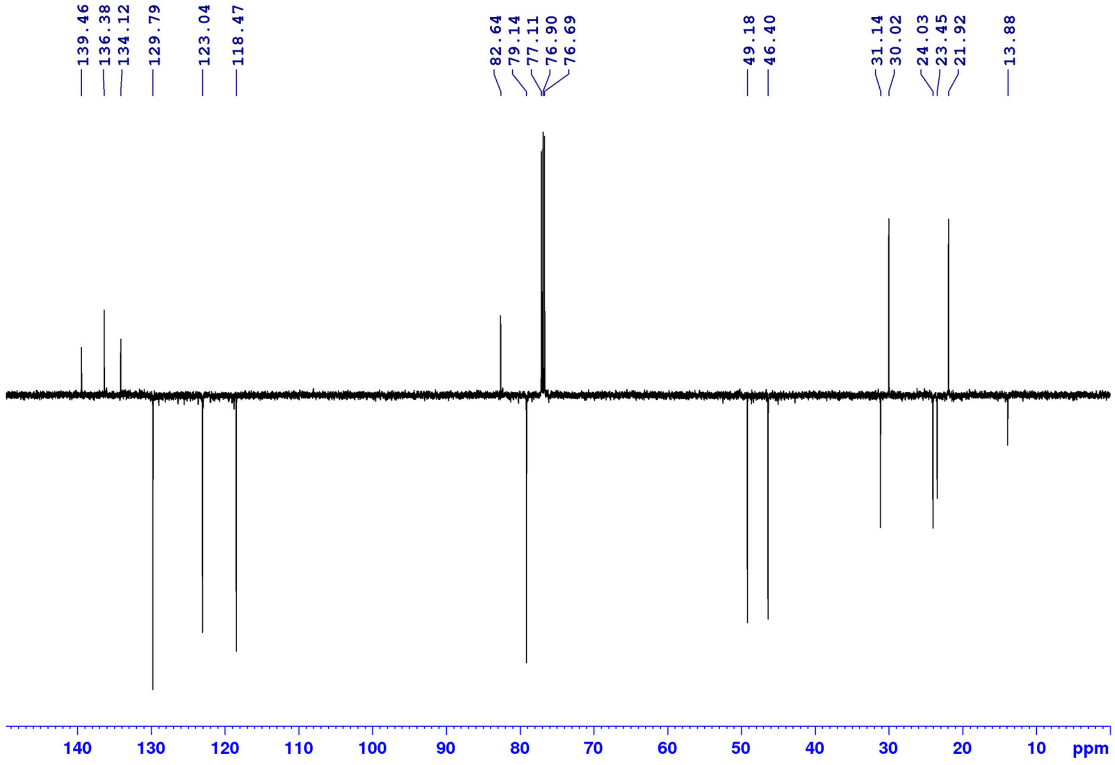

139.46
136.38
134.12
129.79
123.04
118.47
82.64
79.14
77.11
76.90
76.69
49.18
46.40
31.14
30.02
24.03
23.45
21.92
13.88
140
130
120
110
100
90
80
70
60
50
40
30
20
10
ppm

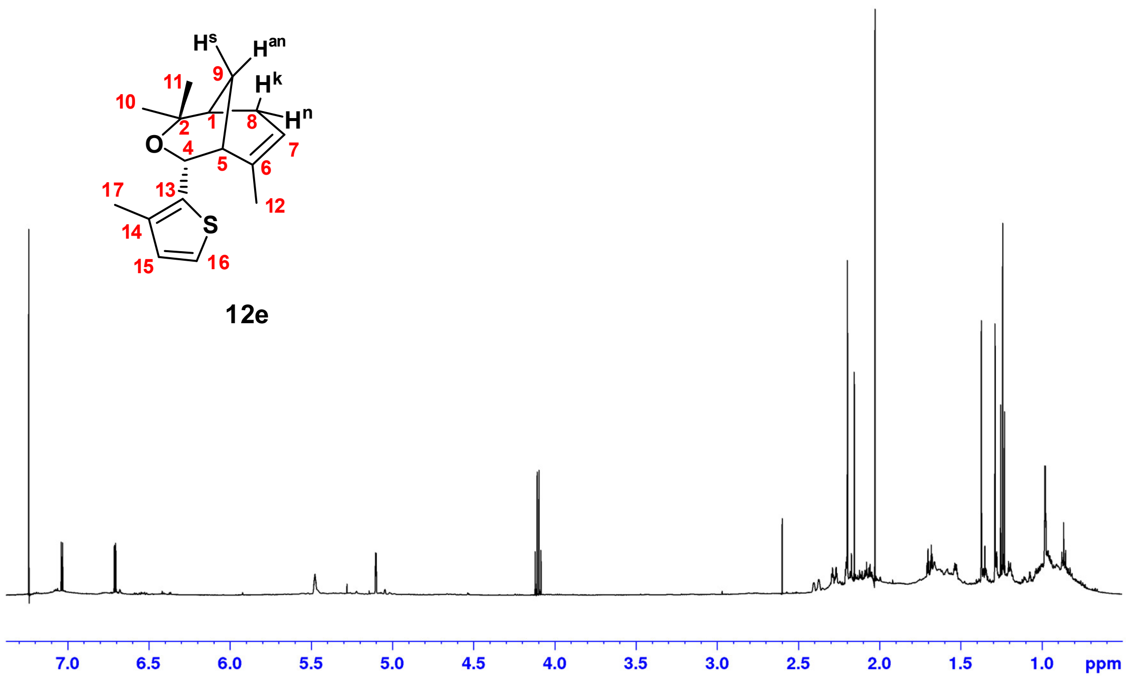

Hs
Han
H k
H n
9
11
10
2
1
8
7
O
4
5
6
13
17
12
14
S
15
16
12e
7.0
6.5
6.0
5.5
5.0
4.5
4.0
3.5
3.0
2.5
2.0
1.5
1.0
ppm


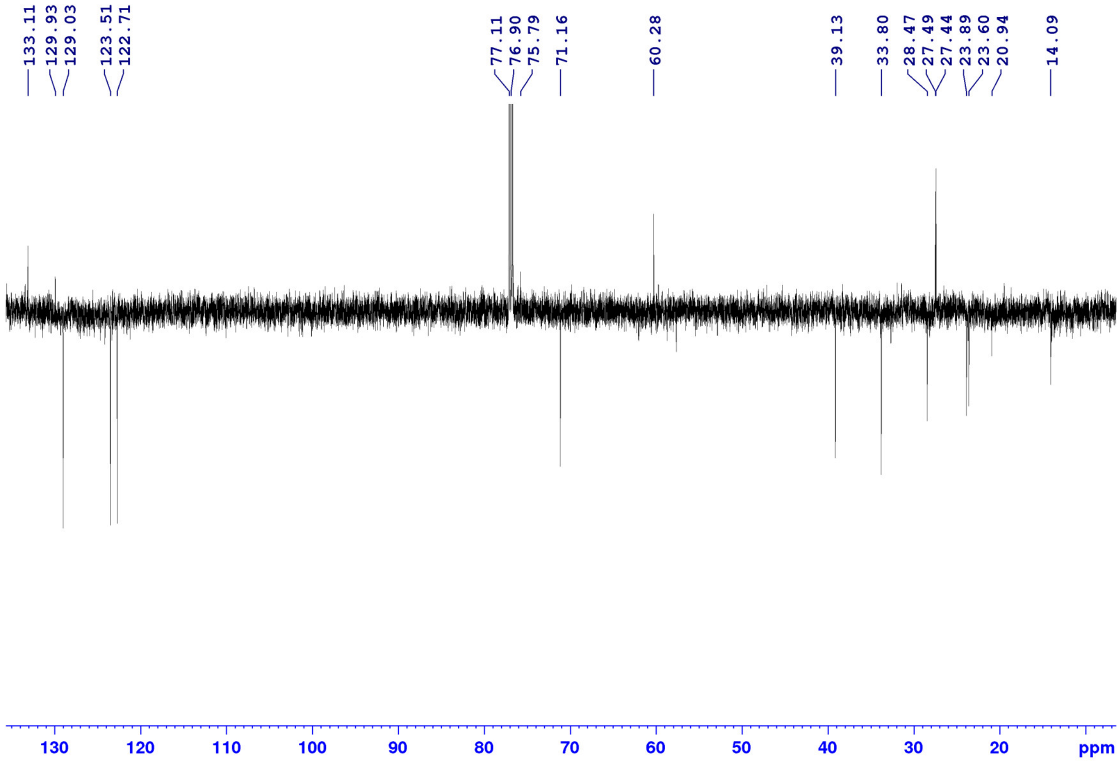

133.11
129.93
129.03
123.51
122.71
77.11
76.90
75.79
71.16
60.28
39.13
33.80
28.47
27.49
27.44
23.89
23.60
20.94
14.09
130
120
110
100
90
80
70
60
50
40
30
20
ppm

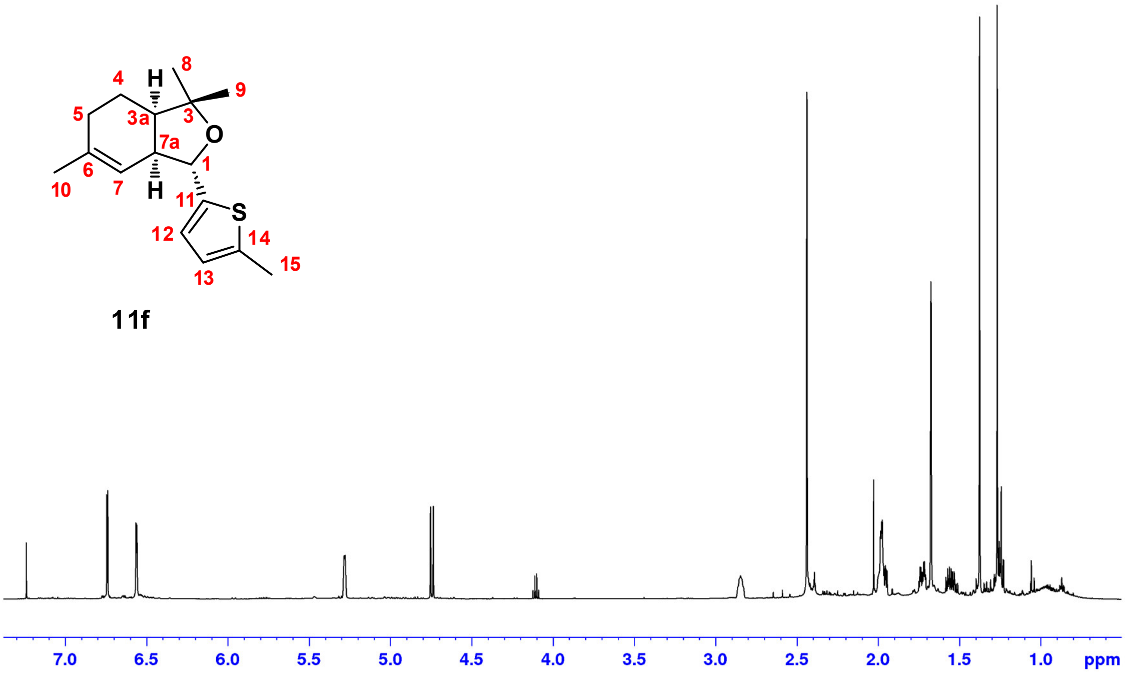
4
H
8
9
5
3a
3
O
7a
6
1
10
7
H
11
S
12
14
15
13
11f
7.0
6.5
6.0
5.5
5.0
4.5
4.0
3.5
3.0
2.5
2.0
1.5
1.0
ppm

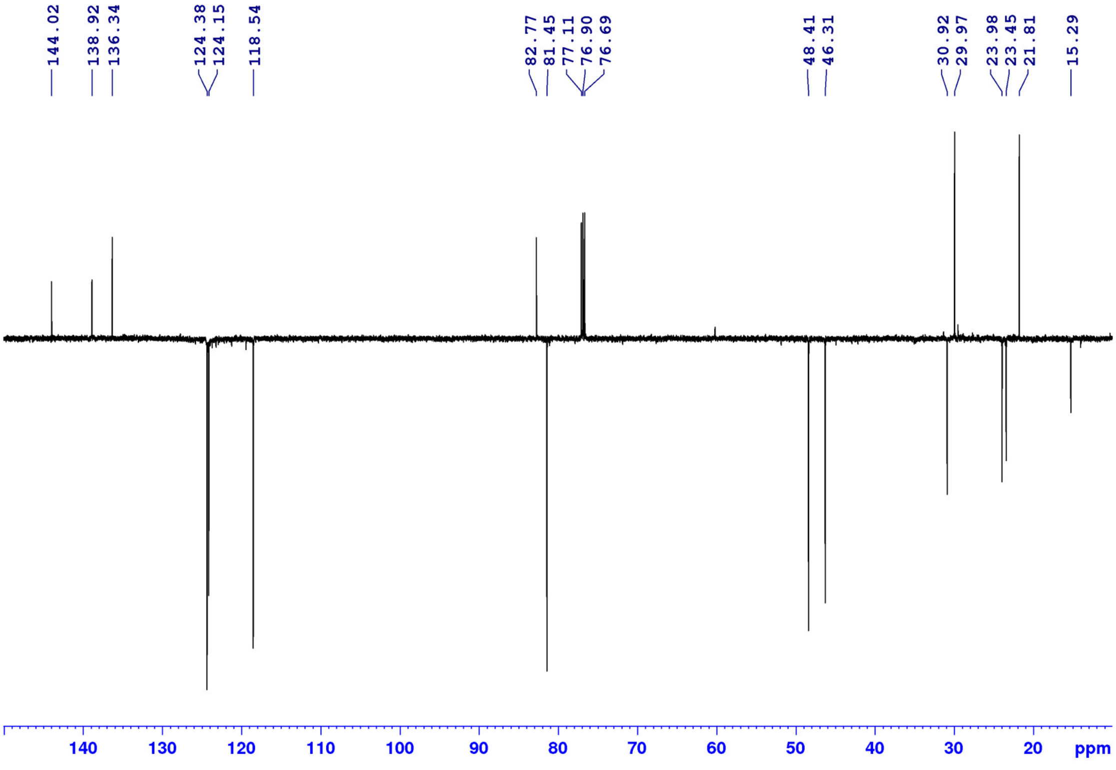
144.02
138.92
136.34
124.38
124.15
118.54
82.77
81.45
77.11
76.90
76.69
48.41
46.31
30.92
29.97
23.98
23.45
21.81
15.29
140
130
120
110
100
90
80
70
60
50
40
30
20
ppm

The NMR spectra of **12f** and **13f** were recorded for their mixture (≈ 3:1).

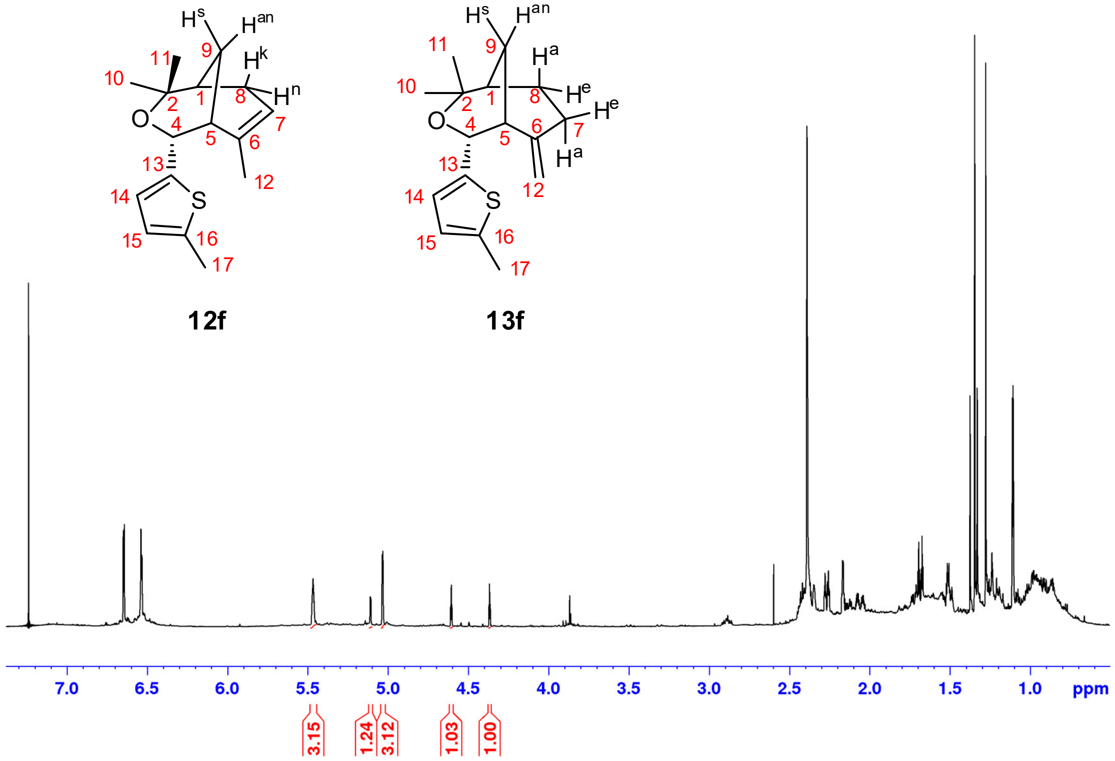


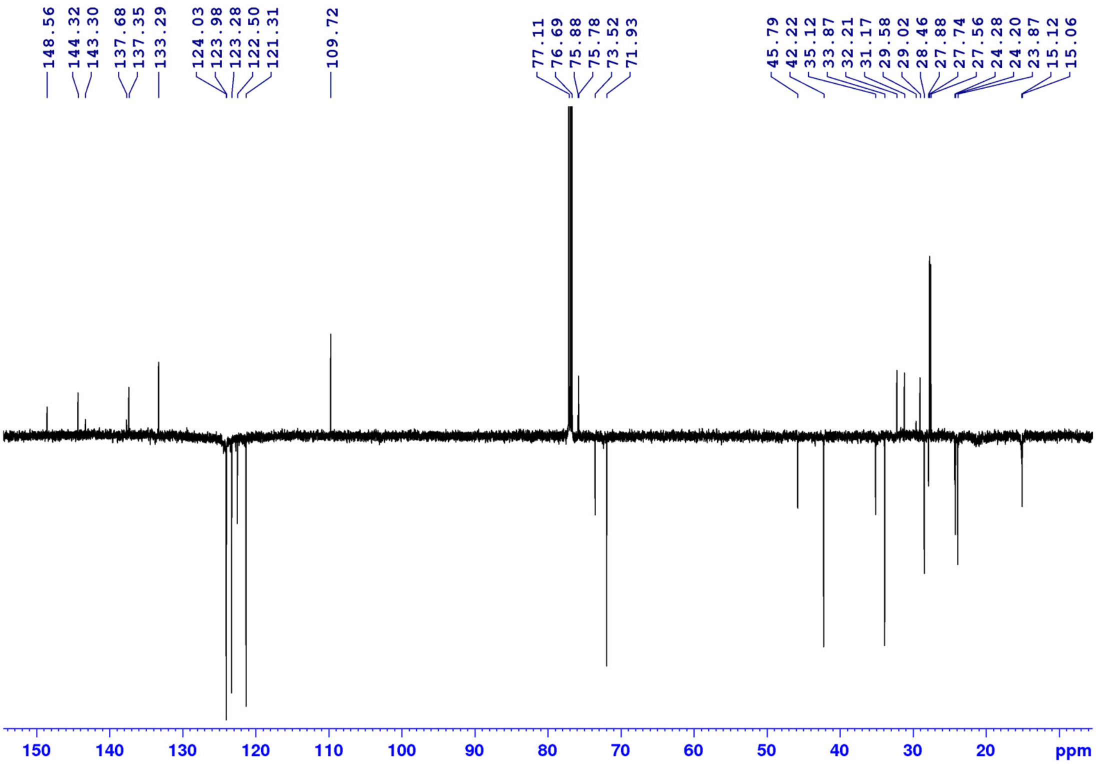

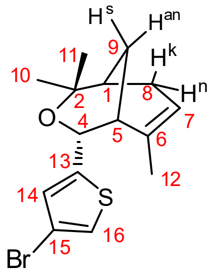

H^s
H^an
H^k
H^n
11
9
10
2
1
8
O
4
5
7
6
13
12
14
S
Br
15
16


**12g**

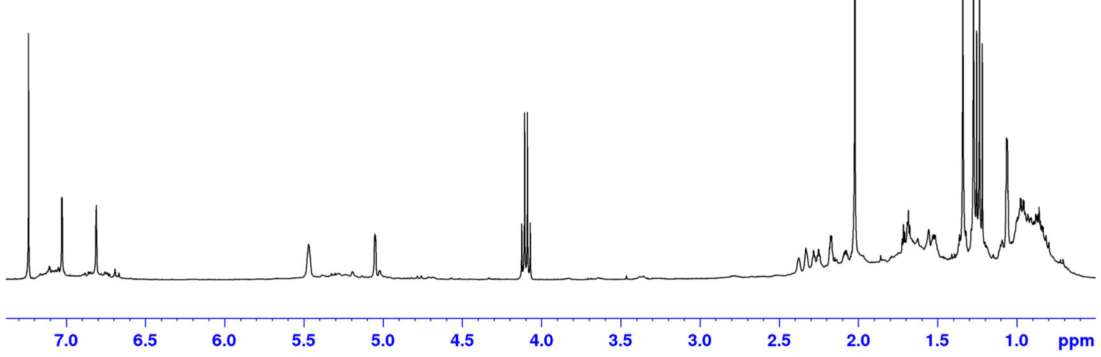

7.0
6.5
6.0
5.5
5.0
4.5
4.0
3.5
3.0
2.5
2.0
1.5
1.0
ppm


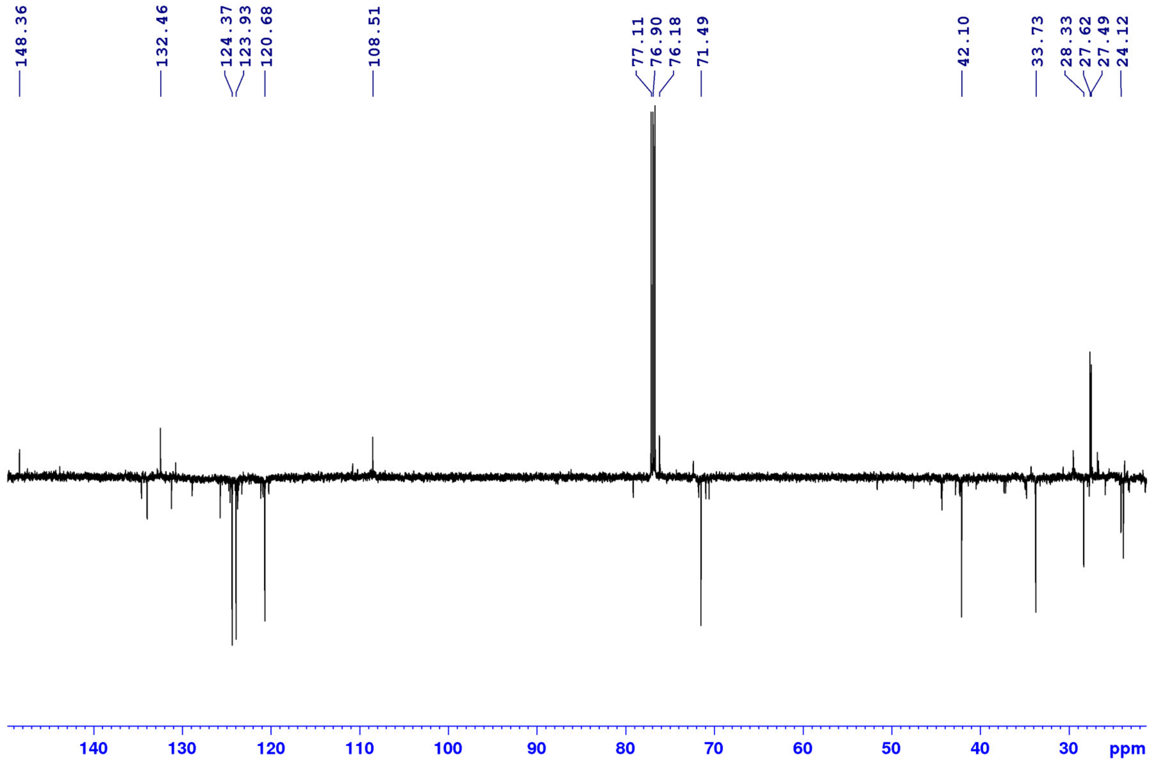

148.36
132.46
124.37
123.93
120.68
108.51
77.11
76.90
76.18
71.49
42.10
33.73
28.33
27.62
27.49
24.12
140
130
120
110
100
90
80
70
60
50
40
30
ppm

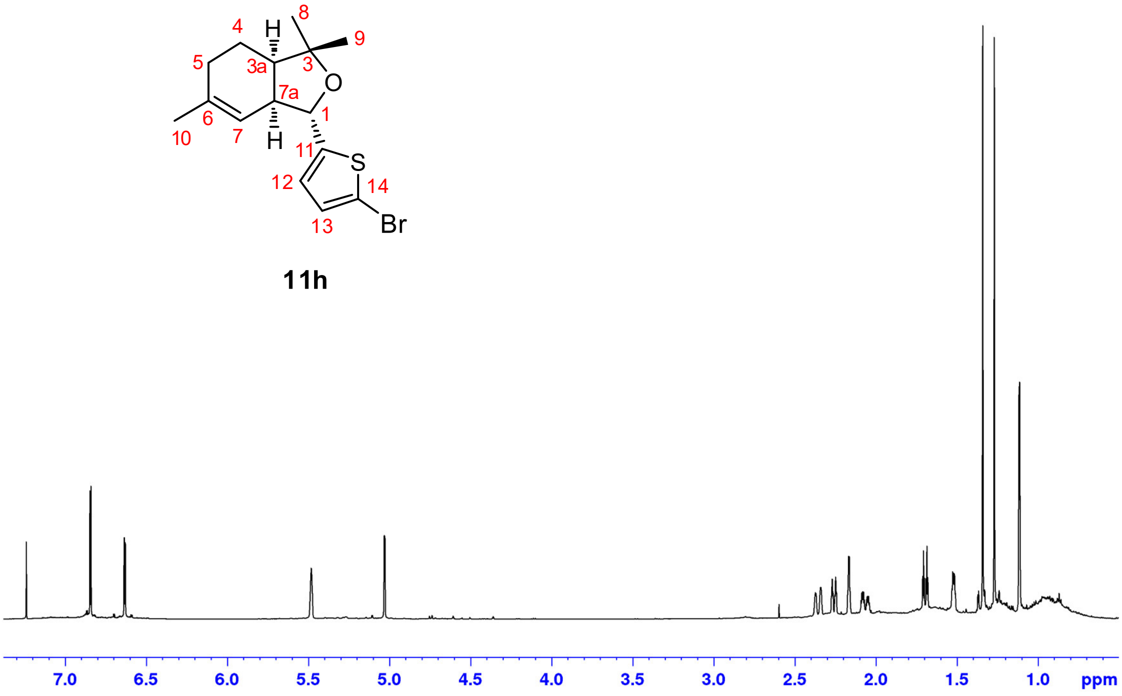
4
8
9
5
3a
3
O
7a
1
10
6
7
11
S
12
14
13
Br
7.0
6.5
6.0
5.5
5.0
4.5
4.0
3.5
3.0
2.5
2.0
1.5
1.0
ppm

**11h**

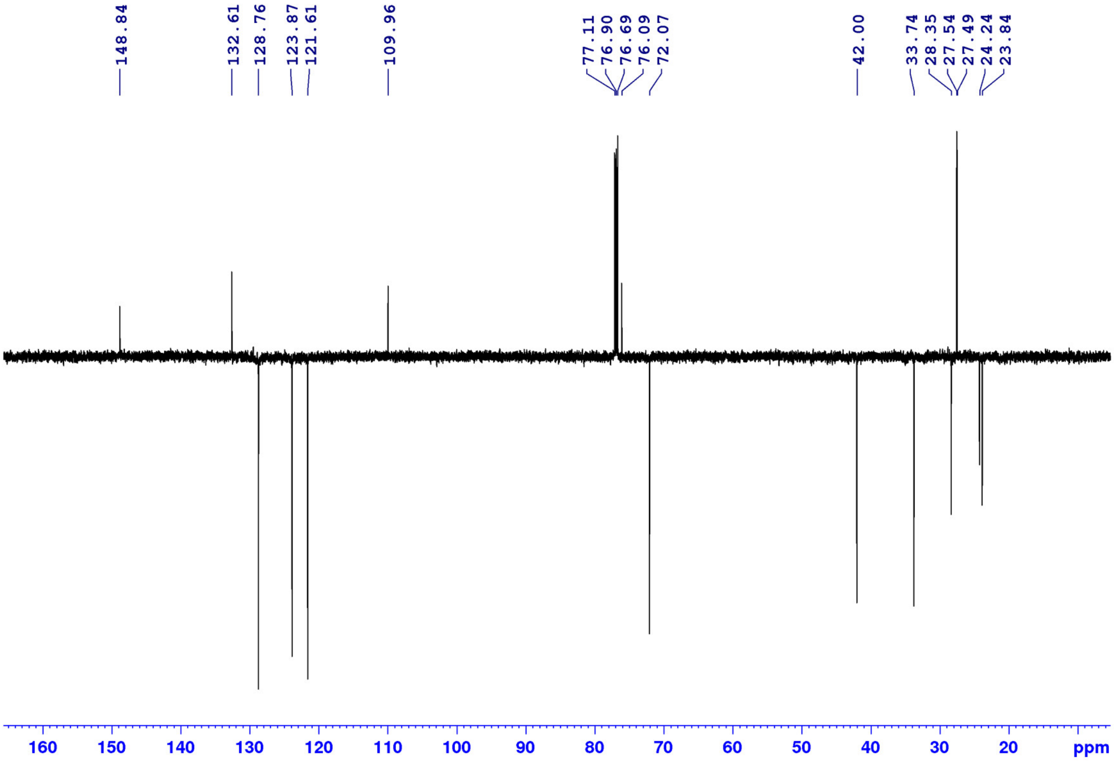
148.84
132.61
128.76
123.87
121.61
109.96
77.11
76.90
76.69
76.09
72.07
42.00
33.74
28.35
27.54
27.49
24.24
23.84
160
150
140
130
120
110
100
90
80
70
60
50
40
30
20
ppm

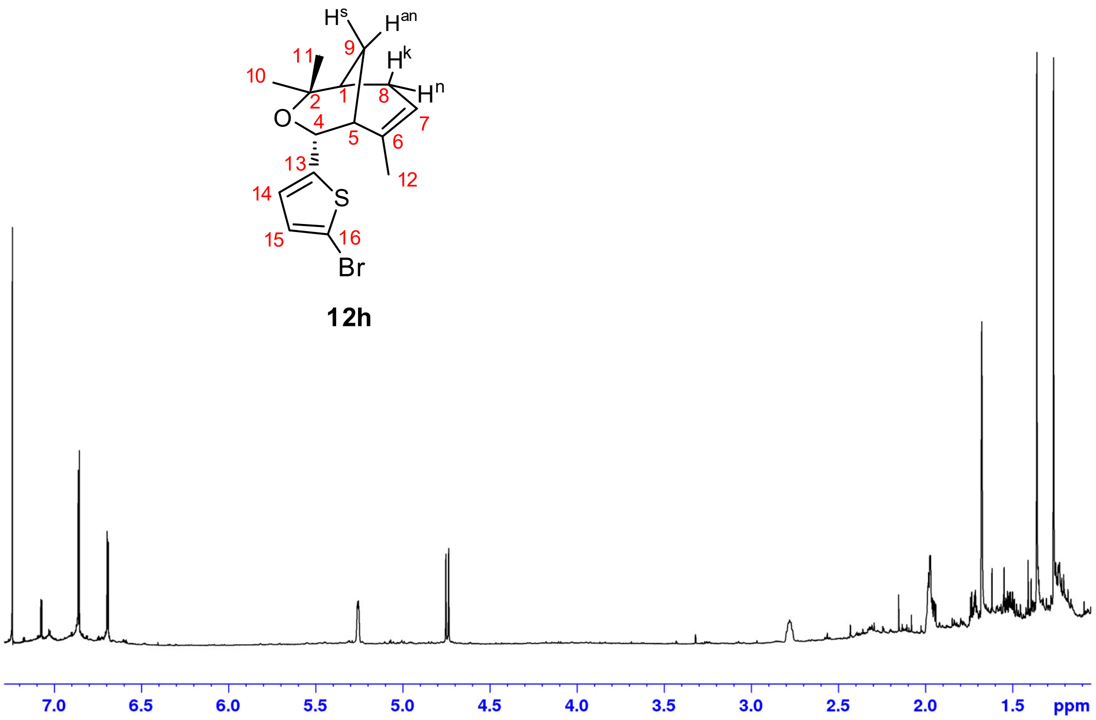
O
S
Br
12h
7.0 6.5 6.0 5.5 5.0 4.5 4.0 3.5 3.0 2.5 2.0 1.5 ppm

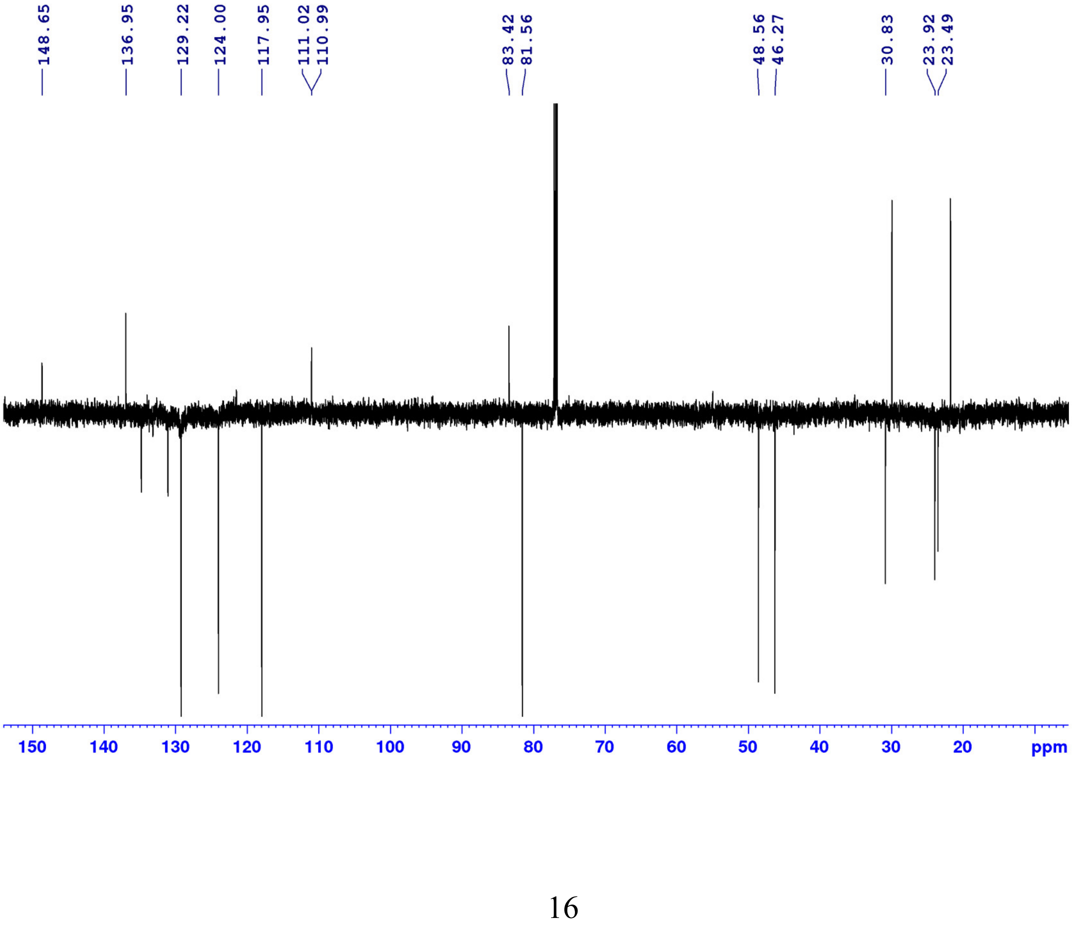
148.65
136.95
129.22
124.00
117.95
111.02
110.99
83.42
81.56
48.56
46.27
30.83
23.92
23.49
150 140 130 120 110 100 90 80 70 60 50 40 30 20 ppm

The NMR spectra of **12i** and **13i** were recorded for their mixture (≈ 5.4:1).

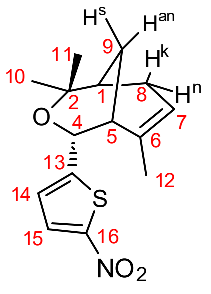


**12i**

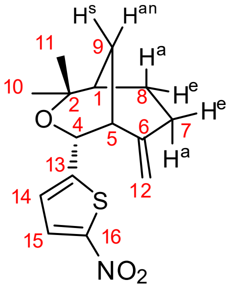


**13i**

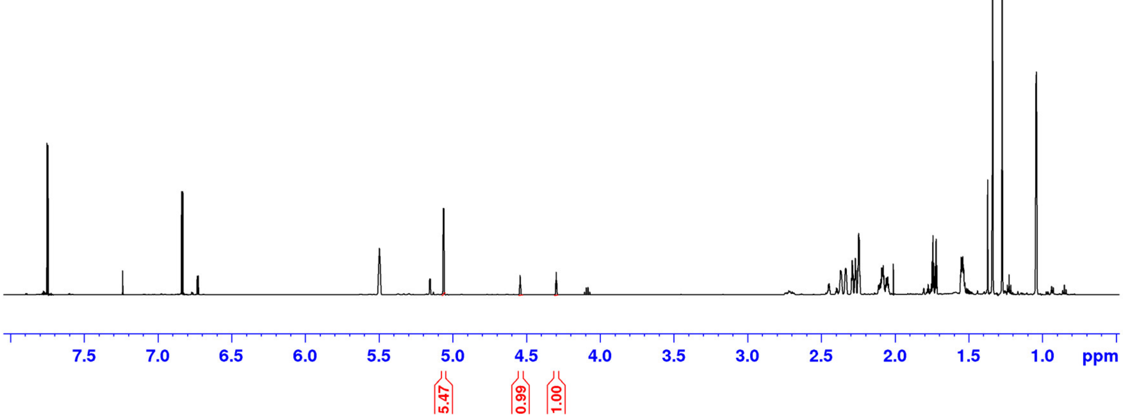


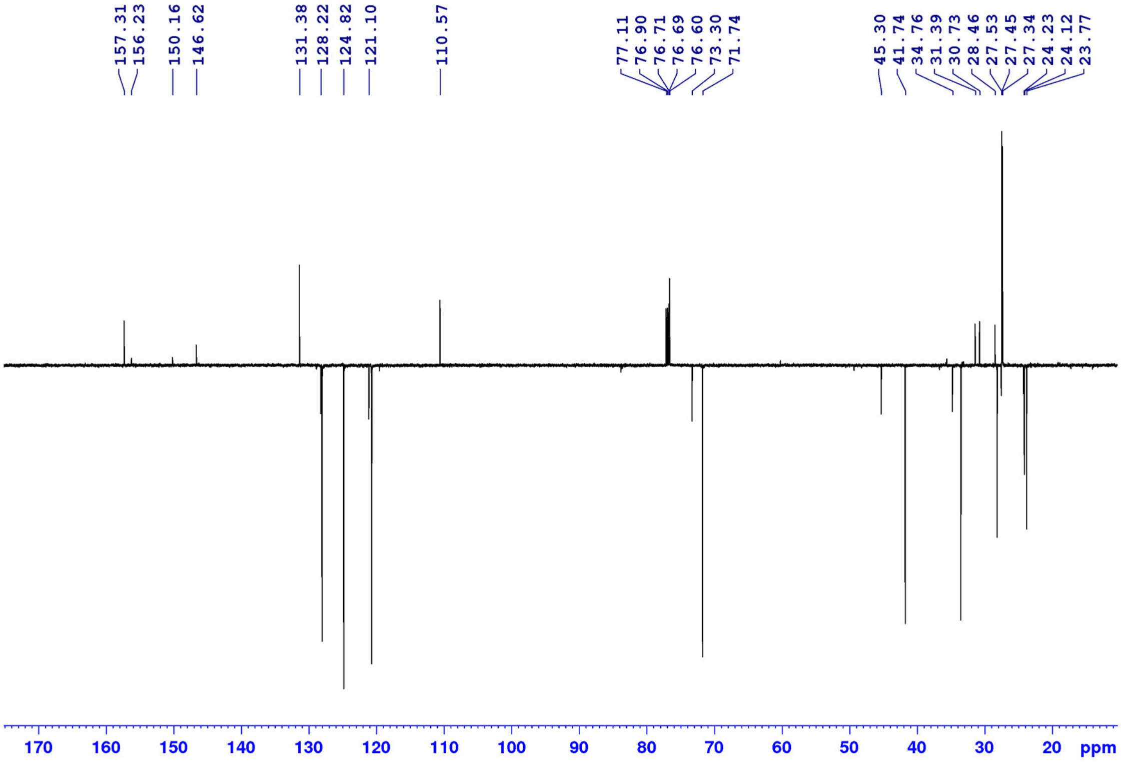

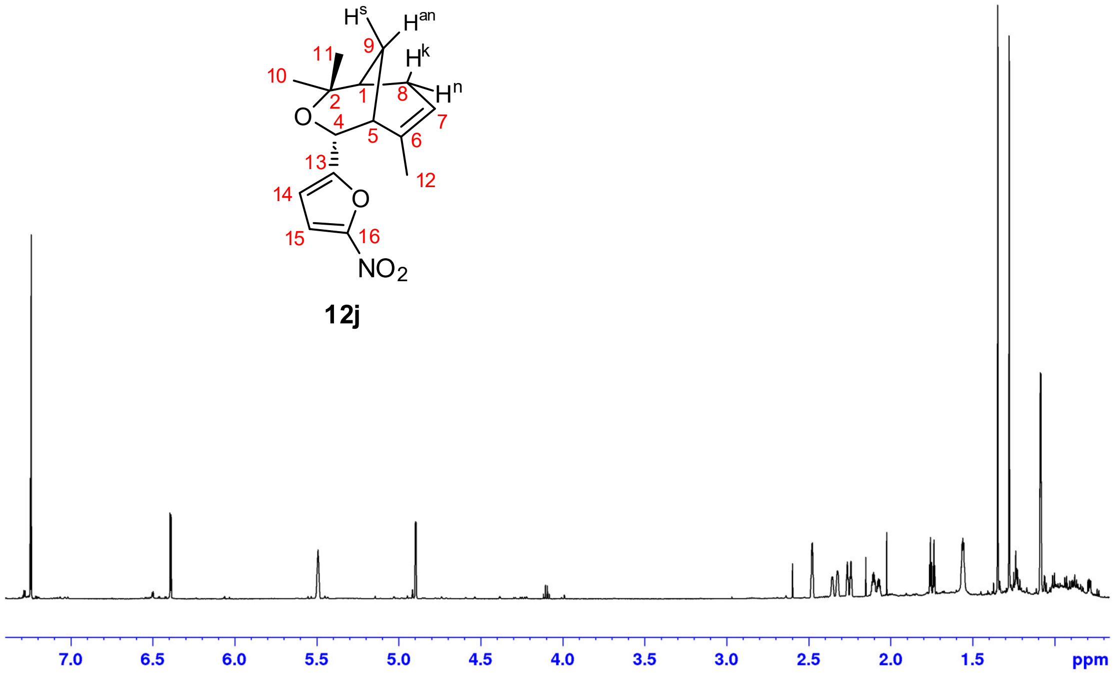

H^s
H^an
H^k
H^n
O
NO2
10
11
9
2
1
8
4
5
6
7
13
12
14
15
16
7.0
6.5
6.0
5.5
5.0
4.5
4.0
3.5
3.0
2.5
2.0
1.5
ppm


**12j**

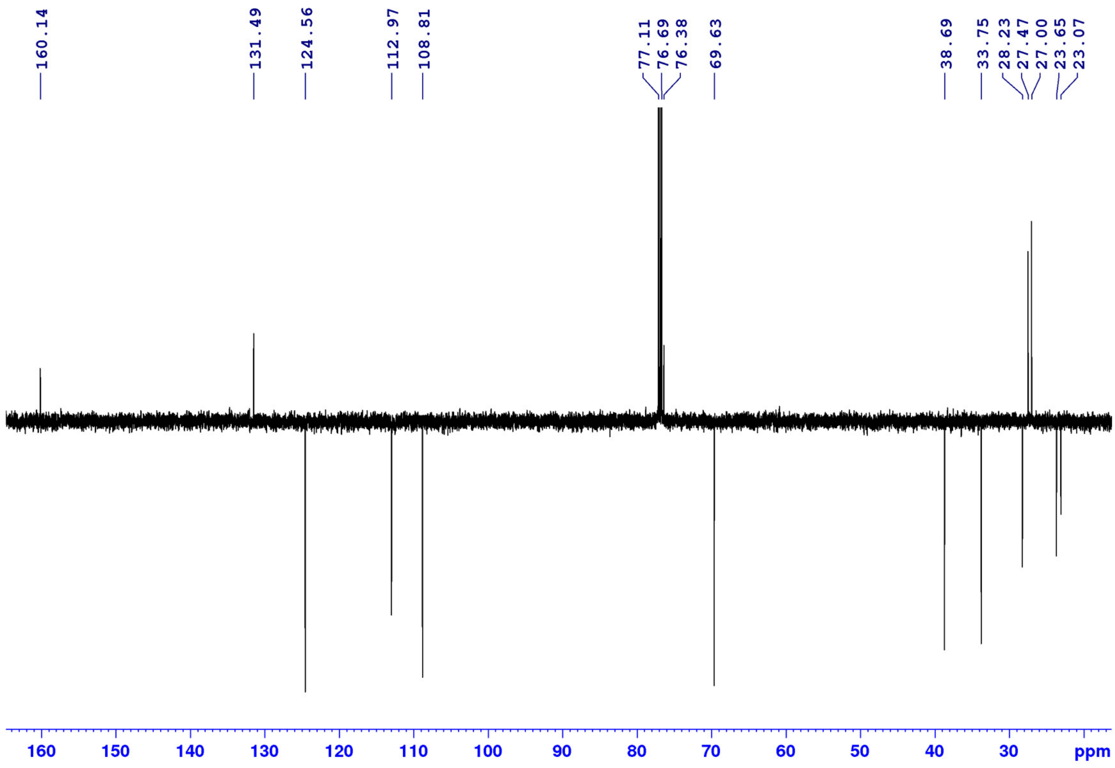

160.14
131.49
124.56
112.97
108.81
77.11
76.69
76.38
69.63
38.69
33.75
28.23
27.47
27.00
23.65
23.07
160
150
140
130
120
110
100
90
80
70
60
50
40
30
ppm

The NMR spectra of compound **11k** were recorded for the mixture (*S*)-**11k** and (*R*)-**11k** isomers (1.5:1).

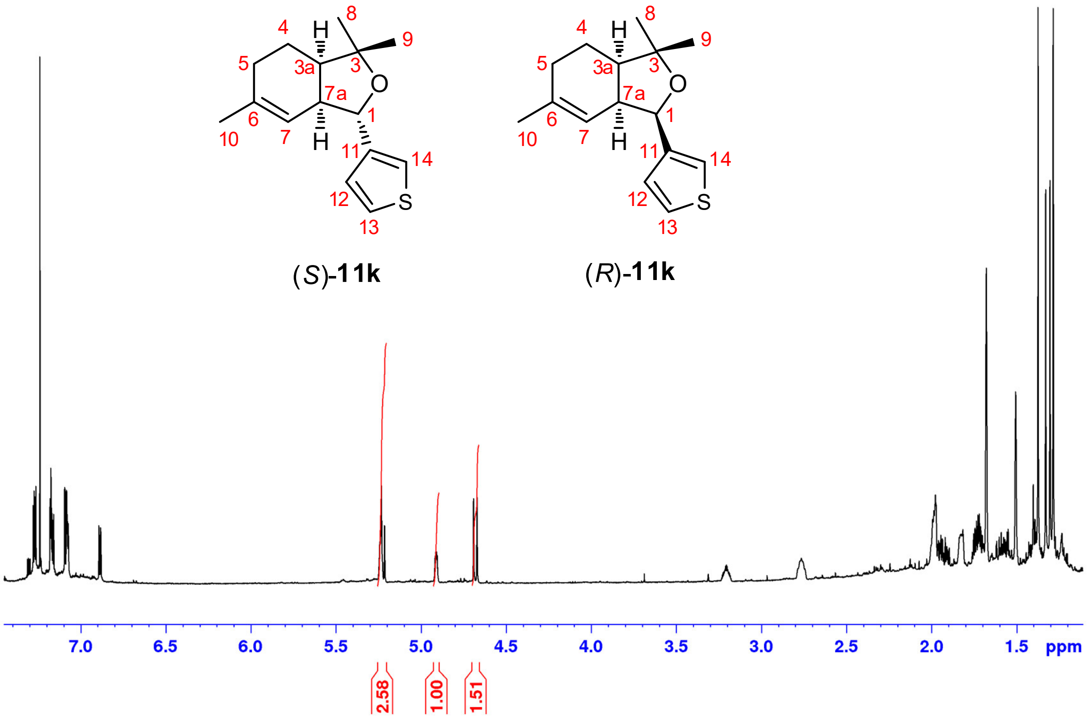


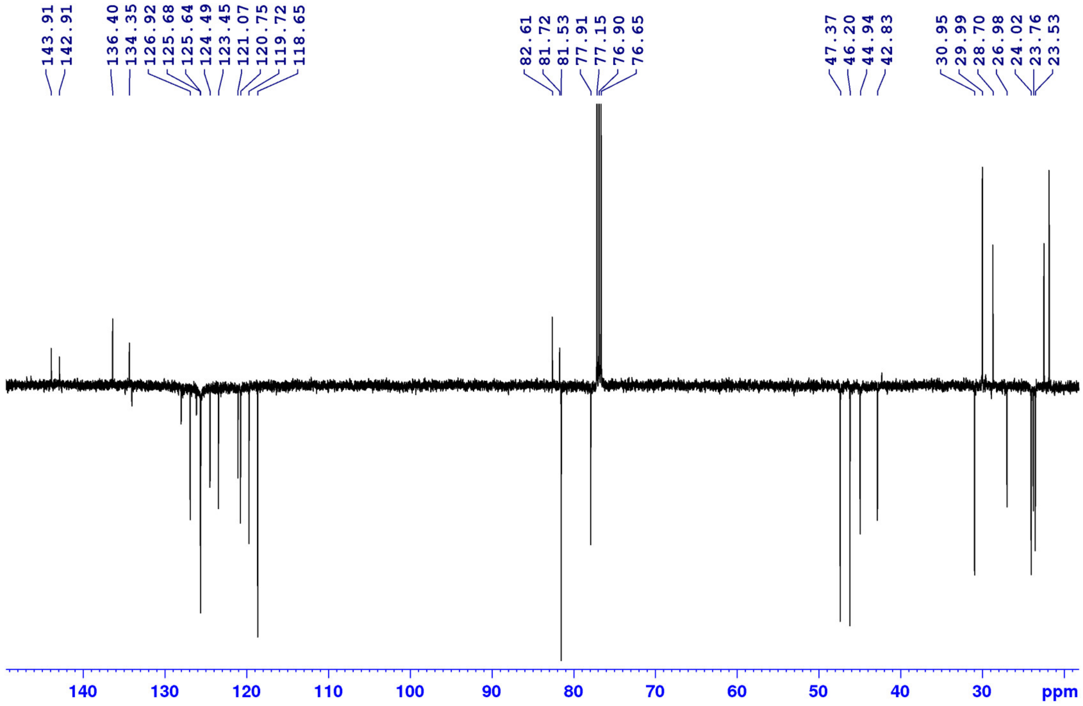

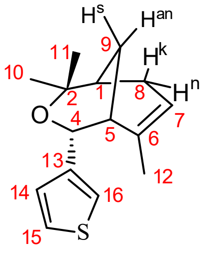
H^s
H^an
9
11
H^k
10
8
H^n
2
1
O
4
7
5
6
13
12
14
16
15
S

**12k**

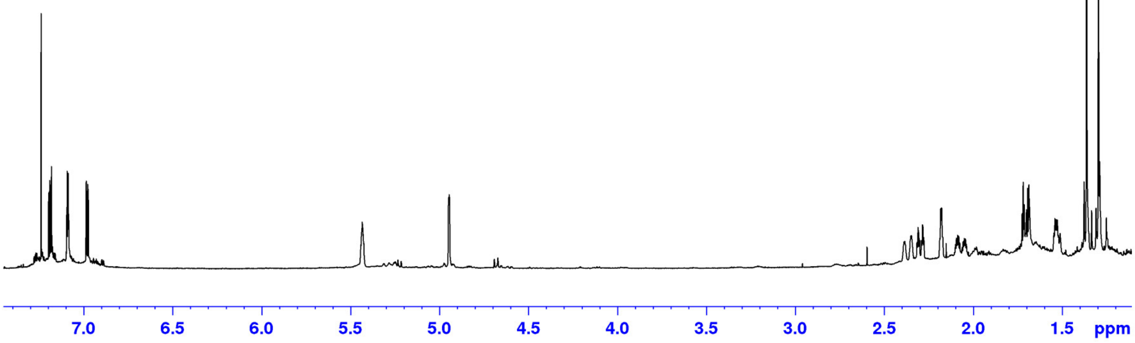
7.0 6.5 6.0 5.5 5.0 4.5 4.0 3.5 3.0 2.5 2.0 1.5 ppm

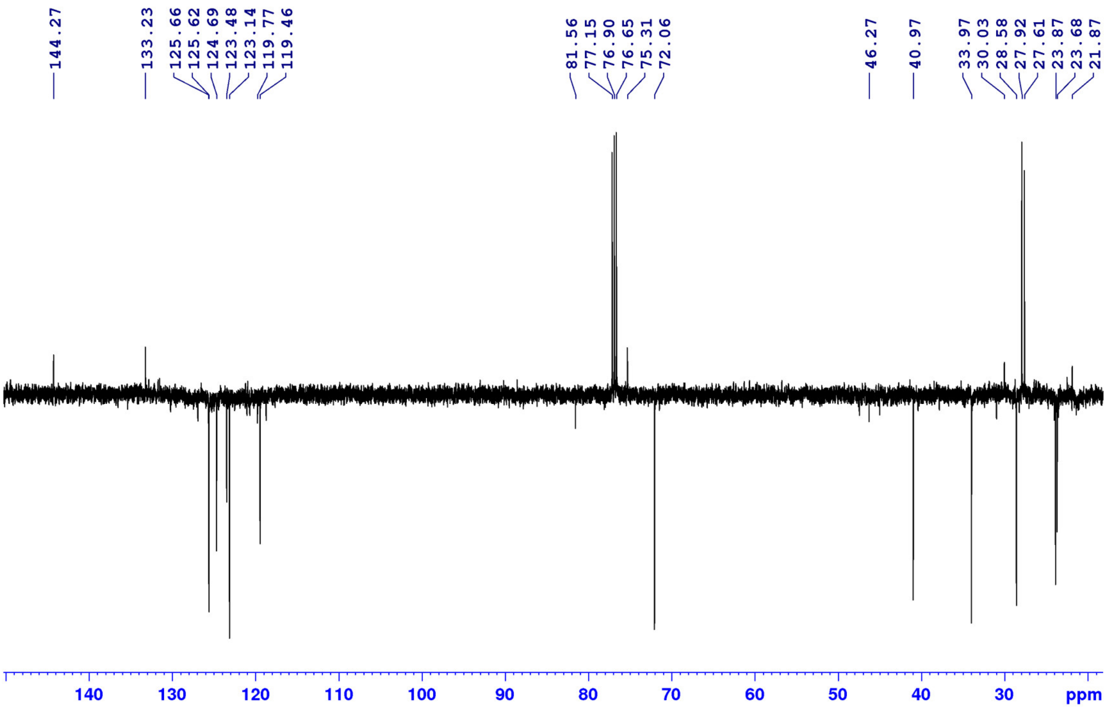
144.27
133.23
125.66
125.62
124.69
123.48
123.14
119.77
119.46
81.56
77.15
76.90
76.65
75.31
72.06
46.27
40.97
33.97
30.03
28.58
27.92
27.61
23.87
23.68
21.87
140 130 120 110 100 90 80 70 60 50 40 30 ppm

## Molecular modelling

The compounds were docked against the crystal structure of TDP1 (PDB ID: 6DIE, resolution 1.78 Å) [2] which was obtained from the Protein Data Bank (PDB) [3,4]. The Scigress version FJ 2.6 program [5] was used to prepare the crystal structure for docking, i.e., the hydrogen atoms were added, the co-crystallised ligand benzene-1,2,4-tricarboxylic acid was removed as well as crystallographic water molecules except HOH 814, 821 and 1078. The crystalline water molecules improve the prediction quality of the docking scaffold as tested by the re-docking of the co-crystallised ligand. The Scigress software suite was also used to build the inhibitors and the MM2 [6] force field was used to optimise the structures. The *S* enantiomeric form was used for the ligands as shown in Scheme 2. The docking centre was defined as the position of a carbon on the ring of benzene-1, 2, 4-tricarboxylic acid (x = -6.052, y = -14.428, z = 33.998) with 10 Å radius. Fifty docking runs were allowed for each ligand with default search efficiency (100%). The basic amino acids lysine and arginine were defined as protonated. Furthermore, aspartic and glutamic acids were assumed to be deprotonated. The GoldScore (GS) [7] and ChemScore (CS) [8,9]. ChemPLP(Piecewise Linear Potential) [10] and ASP (Astex Statistical Potential) [11] scoring functions were implemented to predict binding modes and relative energies of the ligands using the GOLD v5.4.1 software suite.

The 15 compounds were docked into the binding site of TDP1 (PDB ID: 6DIE, resolution 1.78 Å) [2] with three water molecules (HOH814, 821 and 1078). It has been shown that keeping these crystalline water molecules improves the prediction quality of the docking scaffold. The corresponding scores are shown in Table S1 in the Supplementary Information.

The modelling shows that all the ligands have a plausible binding mode and reasonable scores were predicted for all the ligands. Based on the scores the docking algorithm was not able to discern between active and inactive ligands due to their chemical similarity and because they are all relatively potent. Considering **11h**, the molecule occupies a hydrophilic binding region, which contains the amino acids threonine and histidine. The ether oxygen of the molecule forms hydrogen bond with the hydroxyl hydrogen of tyrosine (Tyr204) and it also forms π-π stacking with the same tyrosine as shown in Figure 5.

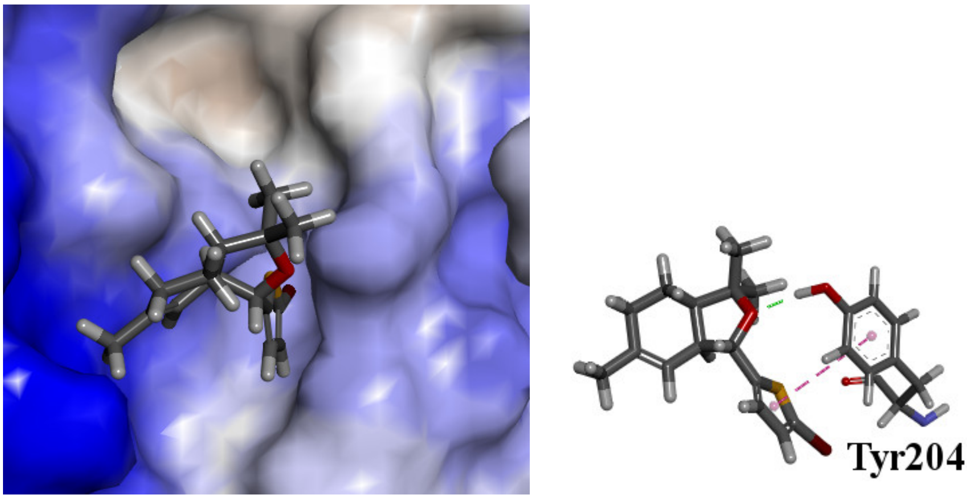


**A** **B**

**Figure 5.** The docked configuration of **11h** in the binding site of TDP1 as predicted using the ChemPLP scoring function. **A**) The protein surface is rendered. The ligand occupies the binding pocket. Blue depicts a hydrophilic region with a partial positive charge on the surface; brown depicts regions with a partial negative charge and grey shows hydrophobic neutral areas. **B**) The hydrogen bond is shown as a green line between **11h** and the amino acid Tyr204 and π-π stacking is shown as a dotted purple line.

The modelling of **12g** showed that it is predicted to occupy a hydrophilic binding region containing amino acids threonine and glutamic acid. The ether oxygen of the ligand forms a hydrogen bond with the hydroxyl group of threonine (Thr216). Also, the molecule forms π-π stacking with the phenyl group of Tyr204, i.e. **11h** and **12g** have the same main interactions within the binding pocket. The bromine group is

located in a small pocket explaining the sensitivity of its location on the thiophene ring. The predicted binding mode of **12g** is shown in Figure 6.

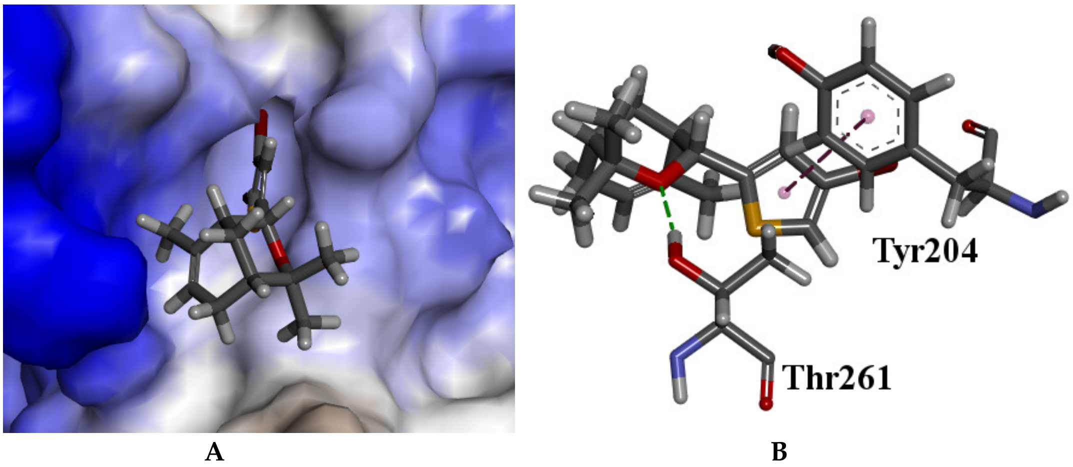


**Figure 6.** The docked configuration of **12g** in the binding site of TDP1 as predicted using the ChemPLP scoring function. **A**) The protein surface is rendered. The ligand occupies the binding pocket. Blue depicts a hydrophilic region with a partial positive charge on the surface; brown depicts region with a partial negative charge and grey shows hydrophobic neutral areas. **B**) Hydrogen bond is shown as a green line between **12g** and the amino acid Thr261. The $\pi$-$\pi$ stacking is shown as a dotted purple line with Tyr204.

## Cartesian coordinates of the ligands

**11a**

```
45 47  0     0            999 V2000
    2.6509   -1.1806    0.8630 C   0  0
    2.4884   -2.5904    1.3712 C   0  0
    1.0414   -2.8775    1.7930 C   0  0
    0.0594   -2.5075    0.6710 C   0  0
    0.2087   -1.0305    0.2893 C   0  0
    1.6068   -0.4855    0.3861 C   0  0
   -1.4335   -2.5739    1.0348 C   0  0
   -1.8211   -1.2985    1.4596 O   0  0
   -0.7637   -0.3848    1.2692 C   0  0
    4.0509   -0.6196    0.8724 C   0  0
   -1.7780   -3.5826    2.1360 C   0  0
   -2.2675   -2.9303   -0.2081 C   0  0
   -0.1457   -0.8932   -0.7608 H   0  0
   -1.2545    0.9731    0.8014 C   0  0
   -0.8063    2.1007    1.3753 C   0  0
   -1.2449    3.2915    0.9468 C   0  0
   -2.1309    3.3897   -0.0576 C   0  0
   -2.5947    2.2729   -0.6580 C   0  0
   -2.1372    1.0879   -0.2063 C   0  0
   -2.5635    4.6107   -0.4777 O   0  0
   -3.4975    2.4276   -1.6833 O   0  0
   -4.0150    1.2834   -2.3211 C   0  0
    3.1558   -2.7482    2.2514 H   0  0
    2.8148   -3.2918    0.5683 H   0  0
    0.8145   -2.2922    2.7145 H   0  0
    0.9482   -3.9580    2.0546 H   0  0
    0.2952   -3.1668   -0.1990 H   0  0
    1.7585    0.5418    0.0158 H   0  0
   -0.2642   -0.2808    2.2625 H   0  0
    4.7248   -1.2677    0.2676 H   0  0
    4.1032    0.4094    0.4522 H   0  0
    4.4414   -0.5780    1.9143 H   0  0
   -2.8752   -3.6130    2.3248 H   0  0
   -1.4576   -4.6107    1.8529 H   0  0
   -1.3007   -3.3244    3.1074 H   0  0
   -2.0353   -3.9589   -0.5661 H   0  0
   -3.3592   -2.8902    0.0089 H   0  0
   -2.0794   -2.2345   -1.0557 H   0  0
   -0.0746    2.0578    2.1986 H   0  0
   -0.8698    4.2111    1.4268 H   0  0
   -2.4853    0.1533   -0.6693 H   0  0
   -3.1966    4.4875   -1.2036 H   0  0
   -4.7248    1.6254   -3.1074 H   0  0
   -3.1978    0.7136   -2.8169 H   0  0
   -4.5782    0.6564   -1.5944 H   0  0
  2  1  1
  1  6  2
  1 10  1
  3  2  1
  2 23  1
  2 24  1
  3  4  1
  3 25  1
  3 26  1
  5  4  1
  7  4  1
```

```
 4 27  1
 6  5  1
 5  9  1
 5 13  1
 6 28  1
 8  7  1
 7 11  1
 7 12  1
 9  8  1
 9 14  1
 9 29  1
10 30  1
10 31  1
10 32  1
11 33  1
11 34  1
11 35  1
12 36  1
12 37  1
12 38  1
14 15  4
14 19  4
15 16  4
15 39  1
16 17  4
16 40  1
17 18  4
17 20  1
18 19  4
18 21  1
19 41  1
20 42  1
21 22  1
22 43  1
22 44  1
22 45  1
M  END
$$$$
```

**12a**

```
45 47  0     0              999 V2000
    1.2202   -0.0830   -1.9186 C   0  0
    2.3261   -0.8201   -1.7241 C   0  0
    2.3088   -2.3169   -1.5577 C   0  0
    0.8924   -2.8921   -1.3544 C   0  0
    0.3156   -2.7668    0.0734 C   0  0
    0.0502   -1.4337    0.3943 O   0  0
   -0.7737   -0.7401   -0.5148 C   0  0
   -0.1489   -0.7318   -1.9150 C   0  0
   -0.0156   -2.1909   -2.3772 C   0  0
   -0.9465   -3.6331    0.2502 C   0  0
    1.3138   -3.2590    1.1383 C   0  0
    1.2970    1.4026   -2.1762 C   0  0
   -1.0941    0.6630   -0.0193 C   0  0
   -2.1596    1.3191   -0.5066 C   0  0
   -2.4406    2.5579   -0.0831 C   0  0
   -1.6738    3.1726    0.8317 C   0  0
   -0.5996    2.5369    1.3461 C   0  0
   -0.3362    1.2932    0.8965 C   0  0
```

| | | | | | |
|---|---|---|---|---|---|
| -1.9729 | 4.4352 | 1.2449 | O | 0 | 0 |
| 0.1619 | 3.2169 | 2.2671 | O | 0 | 0 |
| 1.3740 | 2.6513 | 2.7079 | C | 0 | 0 |
| 3.3163 | -0.3357 | -1.7482 | H | 0 | 0 |
| 2.7421 | -2.7378 | -2.4974 | H | 0 | 0 |
| 3.0059 | -2.6206 | -0.7481 | H | 0 | 0 |
| 0.9410 | -3.9843 | -1.5945 | H | 0 | 0 |
| -1.7581 | -1.2633 | -0.5491 | H | 0 | 0 |
| -0.8094 | -0.1997 | -2.6413 | H | 0 | 0 |
| -1.0139 | -2.6781 | -2.4467 | H | 0 | 0 |
| 0.4198 | -2.2445 | -3.4026 | H | 0 | 0 |
| -1.7339 | -3.4534 | -0.5109 | H | 0 | 0 |
| -0.6964 | -4.7161 | 0.1802 | H | 0 | 0 |
| -1.4183 | -3.4657 | 1.2452 | H | 0 | 0 |
| 0.8394 | -3.3158 | 2.1444 | H | 0 | 0 |
| 1.7041 | -4.2730 | 0.8940 | H | 0 | 0 |
| 2.1802 | -2.5719 | 1.2601 | H | 0 | 0 |
| 2.3423 | 1.7834 | -2.1645 | H | 0 | 0 |
| 0.8699 | 1.6342 | -3.1780 | H | 0 | 0 |
| 0.7309 | 1.9840 | -1.4187 | H | 0 | 0 |
| -2.8066 | 0.8450 | -1.2630 | H | 0 | 0 |
| -3.3163 | 3.0861 | -0.4963 | H | 0 | 0 |
| 0.5428 | 0.7560 | 1.2802 | H | 0 | 0 |
| -1.3145 | 4.7161 | 1.9011 | H | 0 | 0 |
| 1.8538 | 3.3767 | 3.4026 | H | 0 | 0 |
| 1.1812 | 1.7112 | 3.2711 | H | 0 | 0 |
| 2.0600 | 2.4883 | 1.8467 | H | 0 | 0 |

| | | |
|---|---|---|
| 2 | 1 | 2 |
| 1 | 8 | 1 |
| 1 | 12 | 1 |
| 3 | 2 | 1 |
| 2 | 22 | 1 |
| 3 | 4 | 1 |
| 3 | 23 | 1 |
| 3 | 24 | 1 |
| 4 | 5 | 1 |
| 9 | 4 | 1 |
| 4 | 25 | 1 |
| 5 | 6 | 1 |
| 5 | 10 | 1 |
| 5 | 11 | 1 |
| 6 | 7 | 1 |
| 8 | 7 | 1 |
| 7 | 13 | 1 |
| 7 | 26 | 1 |
| 8 | 9 | 1 |
| 8 | 27 | 1 |
| 9 | 28 | 1 |
| 9 | 29 | 1 |
| 10 | 30 | 1 |
| 10 | 31 | 1 |
| 10 | 32 | 1 |
| 11 | 33 | 1 |
| 11 | 34 | 1 |
| 11 | 35 | 1 |
| 12 | 36 | 1 |
| 12 | 37 | 1 |
| 12 | 38 | 1 |
| 13 | 14 | 4 |

13 18  4
14 15  4
14 39  1
15 16  4
15 40  1
16 17  4
16 19  1
17 18  4
17 20  1
18 41  1
19 42  1
20 21  1
21 43  1
21 44  1
21 45  1
M  END
$$$$

**11c**

| | | | | | |
|---|---|---|---|---|---|
| 37 38 0 | 0 | | 999 V2000 | | |
| 1.9763 | -0.7183 | -0.0540 | C | 0 | 0 |
| 1.8579 | -2.1792 | 0.2966 | C | 0 | 0 |
| 0.4252 | -2.5511 | 0.6991 | C | 0 | 0 |
| -0.5828 | -2.0870 | -0.3627 | C | 0 | 0 |
| -0.4804 | -0.5714 | -0.5662 | C | 0 | 0 |
| 0.9060 | -0.0029 | -0.4326 | C | 0 | 0 |
| -2.0603 | -2.2244 | 0.0315 | C | 0 | 0 |
| -2.3772 | -1.0851 | 0.7732 | O | 0 | 0 |
| -1.4545 | -0.0547 | 0.4898 | C | 0 | 0 |
| 3.3621 | -0.1250 | -0.0045 | C | 0 | 0 |
| -2.3711 | -3.4636 | 0.8779 | C | 0 | 0 |
| -2.9641 | -2.2642 | -1.2126 | C | 0 | 0 |
| -0.8474 | -0.3243 | -1.5918 | H | 0 | 0 |
| -2.1552 | 1.2058 | 0.0413 | C | 0 | 0 |
| -1.8744 | 2.4267 | 0.5214 | C | 0 | 0 |
| -2.5617 | 3.6857 | 0.0659 | C | 0 | 0 |
| 2.5411 | -2.4147 | 1.1468 | H | 0 | 0 |
| 2.1912 | -2.7785 | -0.5827 | H | 0 | 0 |
| 0.1894 | -2.0755 | 1.6803 | H | 0 | 0 |
| 0.3662 | -3.6563 | 0.8401 | H | 0 | 0 |
| -0.3595 | -2.6393 | -1.3068 | H | 0 | 0 |
| 1.0246 | 1.0631 | -0.6878 | H | 0 | 0 |
| -0.8966 | 0.1376 | 1.4378 | H | 0 | 0 |
| 4.0406 | -0.6843 | -0.6877 | H | 0 | 0 |
| 3.3814 | 0.9451 | -0.3090 | H | 0 | 0 |
| 3.7705 | -0.1883 | 1.0294 | H | 0 | 0 |
| -3.4597 | -3.5383 | 1.1005 | H | 0 | 0 |
| -2.0692 | -4.3945 | 0.3464 | H | 0 | 0 |
| -1.8536 | -3.4438 | 1.8627 | H | 0 | 0 |
| -2.7464 | -3.1611 | -1.8358 | H | 0 | 0 |
| -4.0406 | -2.3071 | -0.9303 | H | 0 | 0 |
| -2.8429 | -1.3703 | -1.8627 | H | 0 | 0 |
| -2.9267 | 1.0944 | -0.7382 | H | 0 | 0 |
| -1.0949 | 2.5390 | 1.2934 | H | 0 | 0 |
| -3.3322 | 3.4908 | -0.7131 | H | 0 | 0 |
| -3.0640 | 4.1798 | 0.9285 | H | 0 | 0 |
| -1.8156 | 4.3945 | -0.3601 | H | 0 | 0 |

2  1  1
1  6  2

1 10 1
3 2 1
2 17 1
2 18 1
3 4 1
3 19 1
3 20 1
5 4 1
7 4 1
4 21 1
6 5 1
5 9 1
5 13 1
6 22 1
8 7 1
7 11 1
7 12 1
9 8 1
9 14 1
9 23 1
10 24 1
10 25 1
10 26 1
11 27 1
11 28 1
11 29 1
12 30 1
12 31 1
12 32 1
14 15 2
14 33 1
15 16 1
15 34 1
16 35 1
16 36 1
16 37 1
M END
$$$$

**11d**

```
37 39  0     0              999 V2000
    2.0428   -1.1448   -0.2765 C   0  0
    1.8163   -2.5472    0.2273 C   0  0
    0.3674   -2.7598    0.6849 C   0  0
   -0.6227   -2.3263   -0.4066 C   0  0
   -0.4031   -0.8546   -0.7763 C   0  0
    1.0259   -0.3889   -0.7182 C   0  0
   -2.1014   -2.3060    0.0104 C   0  0
   -2.3460   -1.0527    0.5769 O   0  0
   -1.3062   -0.1587    0.2394 C   0  0
    3.4719   -0.6634   -0.3051 C   0  0
   -2.4843   -3.3862    1.0279 C   0  0
   -3.0163   -2.4694   -1.2153 C   0  0
   -1.8299    1.1962   -0.1899 C   0  0
   -1.6002    2.3332    0.4841 C   0  0
   -2.1924    3.3533   -0.1436 C   0  0
   -2.8094    2.8771   -1.2308 C   0  0
   -2.6473    1.4296   -1.3934 S   0  0
   -0.7676   -0.6975   -1.8192 H   0  0
```

```
    2.4967   -2.7479    1.0886 H   0  0
    2.0829   -3.2590   -0.5885 H   0  0
    0.1929   -2.1719    1.6164 H   0  0
    0.2238   -3.8365    0.9393 H   0  0
   -0.4586   -2.9914   -1.2883 H   0  0
    1.2229    0.6314   -1.0864 H   0  0
   -0.7151   -0.0168    1.1780 H   0  0
    4.0905   -1.3442   -0.9327 H   0  0
    3.5699    0.3638   -0.7215 H   0  0
    3.8939   -0.6509    0.7252 H   0  0
   -3.5728   -3.3529    1.2612 H   0  0
   -2.2537   -4.4029    0.6364 H   0  0
   -1.9539   -3.2595    1.9977 H   0  0
   -2.8765   -3.4676   -1.6888 H   0  0
   -4.0905   -2.3799   -0.9349 H   0  0
   -2.8252   -1.7028   -1.9977 H   0  0
   -1.0170    2.4207    1.4109 H   0  0
   -2.1758    4.4029    0.1792 H   0  0
   -3.3799    3.4957   -1.9373 H   0  0
  2  1  1
  1  6  2
  1 10  1
  3  2  1
  2 19  1
  2 20  1
  3  4  1
  3 21  1
  3 22  1
  5  4  1
  7  4  1
  4 23  1
  6  5  1
  5  9  1
  5 18  1
  6 24  1
  8  7  1
  7 11  1
  7 12  1
  9  8  1
  9 13  1
  9 25  1
 10 26  1
 10 27  1
 10 28  1
 11 29  1
 11 30  1
 11 31  1
 12 32  1
 12 33  1
 12 34  1
 13 14  4
 13 17  4
 14 15  4
 14 35  1
 15 16  4
 15 36  1
 16 17  4
 16 37  1
M  END
```

$$$$

**12d**

```
37 39  0     0              999 V2000
    1.1215    0.7999   -0.9218 C   0  0
    2.2039    0.0462   -0.6676 C   0  0
    2.1987   -1.4588   -0.7147 C   0  0
    0.7796   -2.0563   -0.7949 C   0  0
   -0.0034   -2.1147    0.5358 C   0  0
   -0.3552   -0.8365    0.9777 O   0  0
   -1.0557   -0.0396    0.0469 C   0  0
   -0.2162    0.1547   -1.2224 C   0  0
    0.0187   -1.2310   -1.8438 C   0  0
   -1.5331    1.2718    0.6485 C   0  0
   -1.2516   -3.0102    0.4127 C   0  0
    0.8416   -2.7270    1.6686 C   0  0
    1.1974    2.3075   -0.9297 C   0  0
   -2.3038    2.1486   -0.0138 C   0  0
   -2.5692    3.1994    0.7675 C   0  0
   -1.9748    3.0044    1.9496 C   0  0
   -1.2443    1.7357    2.0180 S   0  0
    3.1728    0.5319   -0.4648 H   0  0
    2.7703   -1.7440   -1.6311 H   0  0
    2.7794   -1.8719    0.1375 H   0  0
    0.8857   -3.1085   -1.1616 H   0  0
   -2.0052   -0.5629   -0.2176 H   0  0
   -0.7607    0.7723   -1.9762 H   0  0
   -0.9462   -1.7092   -2.1257 H   0  0
    0.6035   -1.1484   -2.7899 H   0  0
   -1.9229   -2.7424   -0.4295 H   0  0
   -0.9625   -4.0728    0.2464 H   0  0
   -1.8697   -2.9762    1.3388 H   0  0
    0.2273   -2.9127    2.5790 H   0  0
    1.2903   -3.6995    1.3634 H   0  0
    1.6624   -2.0527    1.9991 H   0  0
    2.2311    2.6828   -0.7611 H   0  0
    0.8589    2.7011   -1.9146 H   0  0
    0.5580    2.7521   -0.1384 H   0  0
   -2.6645    2.0292   -1.0443 H   0  0
   -3.1728    4.0728    0.4866 H   0  0
   -2.0238    3.7105    2.7899 H   0  0
  2  1  2
  1  8  1
  1 13  1
  3  2  1
  2 18  1
  3  4  1
  3 19  1
  3 20  1
  4  5  1
  9  4  1
  4 21  1
  5  6  1
  5 11  1
  5 12  1
  6  7  1
  8  7  1
  7 10  1
  7 22  1
```

```
 8  9  1
 8 23  1
 9 24  1
 9 25  1
10 14  4
10 17  4
11 26  1
11 27  1
11 28  1
12 29  1
12 30  1
12 31  1
13 32  1
13 33  1
13 34  1
14 15  4
14 35  1
15 16  4
15 36  1
16 17  4
16 37  1
M  END
$$$$
```

**11e**

```
40 42  0     0              999 V2000
    2.0235   -1.1093   -0.4378 C   0  0
    1.8078   -2.5228    0.0389 C   0  0
    0.3558   -2.7611    0.4731 C   0  0
   -0.6256   -2.3214   -0.6238 C   0  0
   -0.4186   -0.8418   -0.9671 C   0  0
    1.0036   -0.3598   -0.8832 C   0  0
   -2.1102   -2.3257   -0.2268 C   0  0
   -2.3845   -1.0799    0.3435 O   0  0
   -1.3413   -0.1739    0.0492 C   0  0
    3.4460   -0.6080   -0.4358 C   0  0
   -2.4913   -3.4181    0.7783 C   0  0
   -3.0040   -2.4955   -1.4671 C   0  0
   -1.8434    1.1919   -0.3786 C   0  0
   -1.4691    2.3570    0.1774 C   0  0
   -2.0910    3.3608   -0.4518 C   0  0
   -2.8639    2.8511   -1.4161 C   0  0
   -2.7904    1.3909   -1.4902 S   0  0
   -0.7758   -0.6668   -2.0100 H   0  0
   -0.5050    2.5348    1.3162 C   0  0
    2.4792   -2.7301    0.9057 H   0  0
    2.0943   -3.2171   -0.7853 H   0  0
    0.1625   -2.1918    1.4123 H   0  0
    0.2223   -3.8437    0.7073 H   0  0
   -0.4413   -2.9694   -1.5142 H   0  0
    1.1930    0.6687   -1.2321 H   0  0
   -0.7714   -0.0638    1.0031 H   0  0
    4.0835   -1.2682   -1.0664 H   0  0
    3.5356    0.4283   -0.8309 H   0  0
    3.8521   -0.6091    0.6010 H   0  0
   -3.5833   -3.4019    0.9968 H   0  0
   -2.2408   -4.4288    0.3837 H   0  0
   -1.9761   -3.2900    1.7561 H   0  0
   -2.8440   -3.4895   -1.9429 H   0  0
```

-4.0835 -2.4214 -1.2029 H 0 0
-2.8100 -1.7225 -2.2426 H 0 0
-1.9856 4.4288 -0.2189 H 0 0
-3.4878 3.4522 -2.0917 H 0 0
0.4793 2.0744 1.0733 H 0 0
-0.3246 3.6111 1.5348 H 0 0
-0.9065 2.0654 2.2426 H 0 0
2 1 1
1 6 2
1 10 1
3 2 1
2 20 1
2 21 1
3 4 1
3 22 1
3 23 1
5 4 1
7 4 1
4 24 1
6 5 1
5 9 1
5 18 1
6 25 1
8 7 1
7 11 1
7 12 1
9 8 1
9 13 1
9 26 1
10 27 1
10 28 1
10 29 1
11 30 1
11 31 1
11 32 1
12 33 1
12 34 1
12 35 1
13 14 4
13 17 4
14 15 4
14 19 1
15 16 4
15 36 1
16 17 4
16 37 1
19 38 1
19 39 1
19 40 1
M END
$$$$

**12e**
40 42 0 0 999 V2000
1.5978 0.9233 -1.0994 C 0 0
2.7266 0.2198 -0.9128 C 0 0
2.7776 -1.2854 -0.9135 C 0 0
1.3835 -1.9438 -0.8779 C 0 0
0.7069 -2.0106 0.5086 C 0 0

| | | | | | |
|---|---|---|---|---|---|
| 0.3505 | -0.7372 | 0.9588 | O | 0 | 0 |
| -0.4520 | 0.0236 | 0.0797 | C | 0 | 0 |
| 0.2679 | 0.2175 | -1.2620 | C | 0 | 0 |
| 0.5079 | -1.1706 | -1.8757 | C | 0 | 0 |
| -0.9151 | 1.3298 | 0.7113 | C | 0 | 0 |
| -0.5163 | -2.9479 | 0.4949 | C | 0 | 0 |
| 1.6566 | -2.5787 | 1.5802 | C | 0 | 0 |
| 1.6111 | 2.4302 | -1.1883 | C | 0 | 0 |
| -1.8737 | 2.1305 | 0.2108 | C | 0 | 0 |
| -2.0368 | 3.1824 | 1.0218 | C | 0 | 0 |
| -1.1886 | 3.0735 | 2.0485 | C | 0 | 0 |
| -0.3701 | 1.8629 | 1.9746 | S | 0 | 0 |
| -2.6821 | 1.9157 | -1.0383 | C | 0 | 0 |
| 3.6896 | 0.7483 | -0.8164 | H | 0 | 0 |
| 3.2941 | -1.5769 | -1.8602 | H | 0 | 0 |
| 3.4346 | -1.6471 | -0.0941 | H | 0 | 0 |
| 1.5056 | -2.9974 | -1.2355 | H | 0 | 0 |
| -1.3973 | -0.5434 | -0.0884 | H | 0 | 0 |
| -0.3597 | 0.7947 | -1.9806 | H | 0 | 0 |
| -0.4568 | -1.6905 | -2.0704 | H | 0 | 0 |
| 1.0113 | -1.0857 | -2.8674 | H | 0 | 0 |
| -1.2612 | -2.7159 | -0.2943 | H | 0 | 0 |
| -0.2050 | -4.0027 | 0.3190 | H | 0 | 0 |
| -1.0605 | -2.9207 | 1.4664 | H | 0 | 0 |
| 1.1200 | -2.7737 | 2.5366 | H | 0 | 0 |
| 2.1150 | -3.5389 | 1.2515 | H | 0 | 0 |
| 2.4757 | -1.8728 | 1.8413 | H | 0 | 0 |
| 2.6316 | 2.8556 | -1.0636 | H | 0 | 0 |
| 1.2358 | 2.7524 | -2.1858 | H | 0 | 0 |
| 0.9665 | 2.8949 | -0.4132 | H | 0 | 0 |
| -2.7513 | 4.0027 | 0.8713 | H | 0 | 0 |
| -1.1085 | 3.8014 | 2.8674 | H | 0 | 0 |
| -2.1705 | 2.3859 | -1.9082 | H | 0 | 0 |
| -2.8437 | 0.8375 | -1.2567 | H | 0 | 0 |
| -3.6896 | 2.3800 | -0.9435 | H | 0 | 0 |

| | | |
|---|---|---|
| 2 | 1 | 2 |
| 1 | 8 | 1 |
| 1 | 13 | 1 |
| 3 | 2 | 1 |
| 2 | 19 | 1 |
| 3 | 4 | 1 |
| 3 | 20 | 1 |
| 3 | 21 | 1 |
| 4 | 5 | 1 |
| 9 | 4 | 1 |
| 4 | 22 | 1 |
| 5 | 6 | 1 |
| 5 | 11 | 1 |
| 5 | 12 | 1 |
| 6 | 7 | 1 |
| 8 | 7 | 1 |
| 7 | 10 | 1 |
| 7 | 23 | 1 |
| 8 | 9 | 1 |
| 8 | 24 | 1 |
| 9 | 25 | 1 |
| 9 | 26 | 1 |
| 10 | 14 | 4 |
| 10 | 17 | 4 |

```
 11 27  1
 11 28  1
 11 29  1
 12 30  1
 12 31  1
 12 32  1
 13 33  1
 13 34  1
 13 35  1
 14 15  4
 14 18  1
 15 16  4
 15 36  1
 16 17  4
 16 37  1
 18 38  1
 18 39  1
 18 40  1
M  END
$$$$
```

**11f**

```
40 42  0     0              999 V2000
    2.3673   -1.3175    0.3201 C   0  0
    2.1477   -2.7249    0.8128 C   0  0
    0.7018   -2.9455    1.2754 C   0  0
   -0.2947   -2.5072    0.1917 C   0  0
   -0.0817   -1.0320   -0.1677 C   0  0
    1.3459   -0.5617   -0.1114 C   0  0
   -1.7710   -2.4950    0.6166 C   0  0
   -2.0141   -1.2488    1.1989 O   0  0
   -0.9835   -0.3461    0.8560 C   0  0
    3.7945   -0.8305    0.2910 C   0  0
   -2.1461   -3.5871    1.6243 C   0  0
   -2.6925   -2.6472   -0.6056 C   0  0
   -1.5177    1.0054    0.4294 C   0  0
   -1.2612    2.1499    1.0783 C   0  0
   -1.8734    3.1608    0.4559 C   0  0
   -2.5350    2.6845   -0.6071 C   0  0
   -2.3742    1.2292   -0.7468 S   0  0
   -0.4506   -0.8673   -1.2079 H   0  0
   -3.3477    3.5254   -1.5550 C   0  0
    2.8327   -2.9309    1.6691 H   0  0
    2.4127   -3.4290   -0.0102 H   0  0
    0.5293   -2.3647    2.2117 H   0  0
    0.5628   -4.0246    1.5223 H   0  0
   -0.1330   -3.1650   -0.6959 H   0  0
    1.5378    0.4626   -0.4713 H   0  0
   -0.3878   -0.2009    1.7913 H   0  0
    4.4137   -1.5032   -0.3447 H   0  0
    3.8873    0.2008   -0.1162 H   0  0
    4.2200   -0.8259    1.3199 H   0  0
   -3.2334   -3.5588    1.8636 H   0  0
   -1.9151   -4.5989    1.2206 H   0  0
   -1.6110   -3.4698    2.5927 H   0  0
   -2.5548   -3.6410   -1.0889 H   0  0
   -3.7652   -2.5608   -0.3185 H   0  0
   -2.5059   -1.8740   -1.3824 H   0  0
   -0.6455    2.2492    1.9826 H   0  0
```

```
  -1.8376    4.2124    0.7706 H   0  0
  -3.3316    4.5989   -1.2599 H   0  0
  -2.9471    3.4613   -2.5927 H   0  0
  -4.4137    3.2013   -1.5657 H   0  0
 2  1  1
 1  6  2
 1 10  1
 3  2  1
 2 20  1
 2 21  1
 3  4  1
 3 22  1
 3 23  1
 5  4  1
 7  4  1
 4 24  1
 6  5  1
 5  9  1
 5 18  1
 6 25  1
 8  7  1
 7 11  1
 7 12  1
 9  8  1
 9 13  1
 9 26  1
10 27  1
10 28  1
10 29  1
11 30  1
11 31  1
11 32  1
12 33  1
12 34  1
12 35  1
13 14  4
13 17  4
14 15  4
14 36  1
15 16  4
15 37  1
16 17  4
16 19  1
19 38  1
19 39  1
19 40  1
M  END
$$$$
```

**12g**

```
37 39  0     0              999 V2000
   1.3189    0.5152   -0.9479 C   0  0
   2.4097   -0.2296   -0.7030 C   0  0
   2.4180   -1.7344   -0.7558 C   0  0
   1.0043   -2.3454   -0.8309 C   0  0
   0.2294   -2.4176    0.5037 C   0  0
  -0.1304   -1.1448    0.9541 O   0  0
  -0.8433   -0.3487    0.0320 C   0  0
  -0.0143   -0.1419   -1.2424 C   0  0
```

0.2293 -1.5225 -1.8713 C 0 0
-1.3220 0.9569 0.6443 C 0 0
-1.0121 -3.3227 0.3833 C 0 0
1.0860 -3.0285 1.6285 C 0 0
1.3810 2.0235 -0.9509 C 0 0
-2.0897 1.8420 -0.0113 C 0 0
-2.3478 2.8833 0.7847 C 0 0
-1.7584 2.6850 1.9700 C 0 0
-1.0360 1.4106 2.0180 S 0 0
-3.3753 4.3819 0.3072 Br 0 0
3.3753 0.2642 -0.5044 H 0 0
2.9874 -2.0109 -1.6762 H 0 0
3.0070 -2.1451 0.0919 H 0 0
1.1183 -3.3948 -1.2033 H 0 0
-1.7915 -0.8766 -0.2278 H 0 0
-0.5693 0.4735 -1.9903 H 0 0
-0.7330 -2.0082 -2.1493 H 0 0
0.8074 -1.4305 -2.8207 H 0 0
-1.6909 -3.0557 -0.4531 H 0 0
-0.7150 -4.3819 0.2091 H 0 0
-1.6245 -3.2992 1.3134 H 0 0
0.4784 -3.2250 2.5411 H 0 0
1.5420 -3.9950 1.3153 H 0 0
1.9023 -2.3485 1.9585 H 0 0
2.4132 2.4074 -0.7927 H 0 0
1.0278 2.4178 -1.9303 H 0 0
0.7469 2.4596 -0.1505 H 0 0
-2.4463 1.7279 -1.0438 H 0 0
-1.8007 3.3787 2.8207 H 0 0
2 1 2
1 8 1
1 13 1
3 2 1
2 19 1
3 4 1
3 20 1
3 21 1
4 5 1
9 4 1
4 22 1
5 6 1
5 11 1
5 12 1
6 7 1
8 7 1
7 10 1
7 23 1
8 9 1
8 24 1
9 25 1
9 26 1
10 14 4
10 17 4
11 27 1
11 28 1
11 29 1
12 30 1
12 31 1
12 32 1

13 33  1
13 34  1
13 35  1
14 15  4
14 36  1
15 16  4
15 18  1
16 17  4
16 37  1
M  END
$$$$

**11h**
37 39  0     0              999 V2000
2.0442   -1.1447   -0.0593 C   0  0
1.8169   -2.5484    0.4403 C   0  0
0.3685   -2.7605    0.8994 C   0  0
-0.6226   -2.3237   -0.1897 C   0  0
-0.4018   -0.8513   -0.5562 C   0  0
1.0275   -0.3867   -0.4979 C   0  0
-2.1006   -2.3027    0.2296 C   0  0
-2.3414   -1.0515    0.8018 O   0  0
-1.3036   -0.1564    0.4613 C   0  0
3.4737   -0.6644   -0.0874 C   0  0
-2.4837   -3.3862    1.2435 C   0  0
-3.0180   -2.4595   -0.9951 C   0  0
-1.8321    1.1961    0.0312 C   0  0
-1.6044    2.3363    0.7004 C   0  0
-2.2031    3.3533    0.0731 C   0  0
-2.8219    2.8685   -1.0090 C   0  0
-2.6551    1.4255   -1.1685 S   0  0
-0.7664   -0.6915   -1.5986 H   0  0
-3.8033    3.9196   -2.2130 Br  0  0
2.4983   -2.7526    1.2999 H   0  0
2.0814   -3.2579   -0.3782 H   0  0
0.1957   -2.1740    1.8322 H   0  0
0.2241   -3.8376    1.1518 H   0  0
-0.4606   -2.9870   -1.0732 H   0  0
1.2249    0.6347   -0.8630 H   0  0
-0.7109   -0.0120    1.3985 H   0  0
4.0914   -1.3436   -0.7176 H   0  0
3.5723    0.3641   -0.5005 H   0  0
3.8965   -0.6555    0.9426 H   0  0
-3.5717   -3.3519    1.4786 H   0  0
-2.2553   -4.4018    0.8478 H   0  0
-1.9516   -3.2642    2.2130 H   0  0
-2.8818   -3.4565   -1.4720 H   0  0
-4.0914   -2.3678   -0.7125 H   0  0
-2.8260   -1.6911   -1.7754 H   0  0
-1.0177    2.4284    1.6245 H   0  0
-2.1839    4.4018    0.4001 H   0  0
2  1  1
1  6  2
1 10  1
3  2  1
2 20  1
2 21  1
3  4  1
3 22  1

3 23 1
5 4 1
7 4 1
4 24 1
6 5 1
5 9 1
5 18 1
6 25 1
8 7 1
7 11 1
7 12 1
9 8 1
9 13 1
9 26 1
10 27 1
10 28 1
10 29 1
11 30 1
11 31 1
11 32 1
12 33 1
12 34 1
12 35 1
13 14 4
13 17 4
14 15 4
14 36 1
15 16 4
15 37 1
16 17 4
16 19 1
M END
$$$$

**12h**

```
37 39  0     0              999 V2000
    1.1070    0.6911   -1.2171 C   0  0
    2.1885   -0.0627   -0.9600 C   0  0
    2.1793   -1.5681   -0.9904 C   0  0
    0.7587   -2.1634   -1.0588 C   0  0
   -0.0187   -2.2074    0.2756 C   0  0
   -0.3650   -0.9242    0.7068 O   0  0
   -1.0670   -0.1338   -0.2282 C   0  0
   -0.2338    0.0458   -1.5038 C   0  0
   -0.0053   -1.3466   -2.1123 C   0  0
   -1.5326    1.1855    0.3641 C   0  0
   -1.2699   -3.1004    0.1664 C   0  0
    0.8295   -2.8111    1.4107 C   0  0
    1.1876    2.1983   -1.2439 C   0  0
   -2.3087    2.0582   -0.2972 C   0  0
   -2.5569    3.1213    0.4735 C   0  0
   -1.9439    2.9354    1.6474 C   0  0
   -1.2233    1.6663    1.7225 S   0  0
   -1.9973    4.1653    3.0621 Br  0  0
    3.1599    0.4225   -0.7680 H   0  0
    2.7466   -1.8649   -1.9058 H   0  0
    2.7623   -1.9732   -0.1359 H   0  0
    0.8608   -3.2193   -1.4159 H   0  0
   -2.0203   -0.6554   -0.4823 H   0  0
```

```
 -0.7812   0.6575  -2.2603 H   0  0
 -0.9727  -1.8251  -2.3848 H   0  0
  0.5751  -1.2749  -3.0621 H   0  0
 -1.9442  -2.8386  -0.6753 H   0  0
 -0.9843  -4.1653   0.0087 H   0  0
 -1.8838  -3.0561   1.0948 H   0  0
  0.2184  -2.9867   2.3252 H   0  0
  1.2746  -3.7875   1.1128 H   0  0
  1.6532  -2.1357   1.7315 H   0  0
  2.2232   2.5723  -1.0839 H   0  0
  0.8469   2.5805  -2.2325 H   0  0
  0.5527   2.6555  -0.4562 H   0  0
 -2.6840   1.9278  -1.3212 H   0  0
 -3.1599   3.9916   0.1814 H   0  0
  2  1  2
  1  8  1
  1 13  1
  3  2  1
  2 19  1
  3  4  1
  3 20  1
  3 21  1
  4  5  1
  9  4  1
  4 22  1
  5  6  1
  5 11  1
  5 12  1
  6  7  1
  8  7  1
  7 10  1
  7 23  1
  8  9  1
  8 24  1
  9 25  1
  9 26  1
 10 14  4
 10 17  4
 11 27  1
 11 28  1
 11 29  1
 12 30  1
 12 31  1
 12 32  1
 13 33  1
 13 34  1
 13 35  1
 14 15  4
 14 36  1
 15 16  4
 15 37  1
 16 17  4
 16 18  1
M  END
$$$$
```

**12i**

```
39 41  0     0              999 V2000
```

3.7261 6.1979 -3.9751 C 0 0
3.4097 4.8395 -4.0071 C 0 0
2.1612 4.5354 -3.4538 C 0 0
1.4219 5.9918 -2.9023 S 0 0
2.7354 6.9858 -3.3850 C 0 0
2.8265 8.4824 -3.2551 C 0 0
1.5356 9.1361 -3.1363 O 0 0
3.9980 8.9979 -2.3153 C 0 0
4.1220 8.2715 -0.9568 C 0 0
3.4863 8.7241 0.1515 C 0 0
2.6028 9.9733 0.2598 C 0 0
1.0618 9.8368 -1.9509 C 0 0
2.1844 10.6104 -1.1076 C 0 0
3.4754 10.3771 -1.8960 C 0 0
0.2638 8.8227 -1.1149 C 0 0
0.0736 10.8819 -2.5050 C 0 0
1.6312 3.1783 -3.3973 N 0 3
0.5459 2.9108 -2.9237 O 0 0
2.2755 2.2478 -3.8388 O 0 5
4.6518 6.6027 -4.3671 H 0 0
4.0772 4.0970 -4.4285 H 0 0
3.1220 8.8102 -4.2798 H 0 0
4.9922 9.0492 -2.8253 H 0 0
4.9899 7.0394 -0.9127 C 0 0
3.6269 8.1746 1.0972 H 0 0
1.7070 9.7657 0.8870 H 0 0
3.2071 10.7203 0.8272 H 0 0
1.9406 11.6932 -0.9645 H 0 0
2.8414 9.5580 -1.7517 H 0 0
3.3651 10.9656 -2.8391 H 0 0
0.9215 8.0276 -0.6979 H 0 0
-0.2648 9.3192 -0.2698 H 0 0
-0.5135 8.3160 -1.7315 H 0 0
-0.4225 11.4496 -1.6845 H 0 0
0.5974 11.6144 -3.1615 H 0 0
-0.7277 10.4042 -3.1149 H 0 0
4.5544 6.2235 -1.5285 H 0 0
6.0078 7.2737 -1.2978 H 0 0
5.1063 6.6439 0.1213 H 0 0
1 2 4
2 3 4
3 4 4
4 5 4
1 5 4
6 5 1
6 7 1
6 8 1
8 9 1
9 10 2
10 11 1
7 12 1
11 13 1
12 13 1
13 14 1
14 8 1
12 15 1
12 16 1
17 18 2
17 19 1

```
  1 20  1
  2 21  1
  6 22  1
  8 23  1
  9 24  1
 10 25  1
 11 26  1
 11 27  1
 13 28  1
 14 29  1
 14 30  1
 15 31  1
 15 32  1
 15 33  1
 16 34  1
 16 35  1
 16 36  1
 17  3  1
 24 37  1
 24 38  1
 24 39  1
M  END
$$$$
```

**12j**

```
39 41  0     0              999 V2000
    4.1590    2.1113   -0.6165 C   0  0
    4.6612    1.0258    0.1028 C   0  0
    3.6881    0.6768    1.0451 C   0  0
    2.6125    1.5176    0.9218 O   0  0
    2.9011    2.3998   -0.0811 C   0  0
    1.9857    3.4787   -0.5851 C   0  0
    0.6338    3.2665   -0.2380 O   0  0
    2.4535    4.9344   -0.4276 C   0  0
    3.1028    5.3015    0.8936 C   0  0
    2.4265    5.9421    1.8618 C   0  0
    0.9733    6.3093    1.7452 C   0  0
    0.1003    4.0352    0.8237 C   0  0
    0.3192    5.5535    0.5779 C   0  0
    1.2053    5.7961   -0.6526 C   0  0
    0.6154    3.4535    2.1536 C   0  0
   -1.4152    3.7394    0.7723 C   0  0
    3.8228   -0.4169    1.9911 N   0  3
    2.9469   -0.6800    2.7890 O   0  0
    4.8247   -1.1027    2.0080 O   0  5
    4.6498    2.6304   -1.4303 H   0  0
    5.6206    0.5466   -0.0500 H   0  0
    1.9486    3.2805   -1.6825 H   0  0
    3.1951    5.1733   -1.2274 H   0  0
    4.5602    4.9600    1.0860 C   0  0
    2.9342    6.2255    2.7989 H   0  0
    0.9168    7.4118    1.5848 H   0  0
    0.4339    6.1055    2.6978 H   0  0
   -0.6637    6.0488    0.3865 H   0  0
    1.4750    6.8743   -0.7552 H   0  0
    0.6786    5.5084   -1.5940 H   0  0
    1.7103    3.5841    2.2905 H   0  0
    0.1045    3.9057    3.0332 H   0  0
    0.4207    2.3583    2.2118 H   0  0
```

```
  -1.9617    4.2822    1.5777 H   0  0
  -1.8561    4.0416   -0.2057 H   0  0
  -1.6272    2.6526    0.8983 H   0  0
   5.1677    5.3390    0.2338 H   0  0
   4.7014    3.8616    1.1714 H   0  0
   4.9780    5.4096    2.0146 H   0  0
  1  2  4
  2  3  4
  3  4  4
  4  5  4
  1  5  4
  6  5  1
  6  7  1
  6  8  1
  8  9  1
  9 10  2
 10 11  1
  7 12  1
 11 13  1
 12 13  1
 13 14  1
 14  8  1
 12 15  1
 12 16  1
 17 18  2
 17 19  1
  1 20  1
  2 21  1
  6 22  1
  8 23  1
  9 24  1
 10 25  1
 11 26  1
 11 27  1
 13 28  1
 14 29  1
 14 30  1
 15 31  1
 15 32  1
 15 33  1
 16 34  1
 16 35  1
 16 36  1
 17  3  1
 24 37  1
 24 38  1
 24 39  1
M  CHG  1  17   1
M  CHG  1  19  -1
M  END
$$$$
```

**11k**

```
37 39  0     0              999 V2000
   2.0221   -1.0910   -0.1883 C   0  0
   1.8108   -2.5133    0.2636 C   0  0
   0.3615   -2.7607    0.7021 C   0  0
  -0.6254   -2.3004   -0.3813 C   0  0
  -0.4241   -0.8135   -0.6927 C   0  0
```

| | | | | | |
|---|---|---|---|---|---|
| 0.9982 | -0.3324 | -0.6092 | C | 0 | 0 |
| -2.1102 | -2.3207 | 0.0155 | C | 0 | 0 |
| -2.4127 | -1.0680 | 0.5576 | O | 0 | 0 |
| -1.3417 | -0.1766 | 0.3436 | C | 0 | 0 |
| 3.4450 | -0.5907 | -0.1903 | C | 0 | 0 |
| -2.4802 | -3.4002 | 1.0382 | C | 0 | 0 |
| -2.9941 | -2.5256 | -1.2270 | C | 0 | 0 |
| -1.8231 | 1.1869 | -0.0770 | C | 0 | 0 |
| -1.3207 | 2.3541 | 0.3417 | C | 0 | 0 |
| -1.9917 | 3.3485 | -0.2522 | C | 0 | 0 |
| -3.0427 | 2.8767 | -1.1602 | S | 0 | 0 |
| -2.8148 | 1.4445 | -0.9408 | C | 0 | 0 |
| -0.8018 | -0.6055 | -1.7230 | H | 0 | 0 |
| 2.4876 | -2.7358 | 1.1223 | H | 0 | 0 |
| 2.0927 | -3.1921 | -0.5750 | H | 0 | 0 |
| 0.1741 | -2.2086 | 1.6527 | H | 0 | 0 |
| 0.2293 | -3.8475 | 0.9169 | H | 0 | 0 |
| -0.4374 | -2.9263 | -1.2867 | H | 0 | 0 |
| 1.1854 | 0.7027 | -0.9392 | H | 0 | 0 |
| -0.8037 | -0.0847 | 1.3176 | H | 0 | 0 |
| 4.0762 | -1.2402 | -0.8381 | H | 0 | 0 |
| 3.5322 | 0.4522 | -0.5685 | H | 0 | 0 |
| 3.8606 | -0.6101 | 0.8425 | H | 0 | 0 |
| -3.5727 | -3.3927 | 1.2544 | H | 0 | 0 |
| -2.2185 | -4.4148 | 0.6613 | H | 0 | 0 |
| -1.9678 | -3.2490 | 2.0142 | H | 0 | 0 |
| -2.8195 | -3.5264 | -1.6831 | H | 0 | 0 |
| -4.0762 | -2.4590 | -0.9717 | H | 0 | 0 |
| -2.8009 | -1.7638 | -2.0142 | H | 0 | 0 |
| -0.4927 | 2.4769 | 1.0524 | H | 0 | 0 |
| -1.7826 | 4.4148 | -0.0887 | H | 0 | 0 |
| -3.4043 | 0.6717 | -1.4525 | H | 0 | 0 |

| | | |
|---|---|---|
| 2 | 1 | 1 |
| 1 | 6 | 2 |
| 1 | 10 | 1 |
| 3 | 2 | 1 |
| 2 | 19 | 1 |
| 2 | 20 | 1 |
| 3 | 4 | 1 |
| 3 | 21 | 1 |
| 3 | 22 | 1 |
| 5 | 4 | 1 |
| 7 | 4 | 1 |
| 4 | 23 | 1 |
| 6 | 5 | 1 |
| 5 | 9 | 1 |
| 5 | 18 | 1 |
| 6 | 24 | 1 |
| 8 | 7 | 1 |
| 7 | 11 | 1 |
| 7 | 12 | 1 |
| 9 | 8 | 1 |
| 9 | 13 | 1 |
| 9 | 25 | 1 |
| 10 | 26 | 1 |
| 10 | 27 | 1 |
| 10 | 28 | 1 |
| 11 | 29 | 1 |
| 11 | 30 | 1 |

11 31  1
12 32  1
12 33  1
12 34  1
14 13  4
13 17  4
15 14  4
14 35  1
15 16  4
15 36  1
17 16  4
17 37  1
M  END
$$$$

**12k**

37 39  0     0              999 V2000
    1.1326    0.8580   -0.8170 C   0  0
    2.2234    0.1221   -0.5467 C   0  0
    2.2432   -1.3826   -0.5934 C   0  0
    0.8342   -2.0019   -0.6861 C   0  0
    0.0383   -2.0661    0.6373 C   0  0
   -0.3422   -0.7918    1.0660 O   0  0
   -1.0406   -0.0176    0.1194 C   0  0
   -0.1901    0.1911   -1.1373 C   0  0
    0.0737   -1.1933   -1.7492 C   0  0
   -1.5040    1.2815    0.7322 C   0  0
   -1.1928   -2.9834    0.5051 C   0  0
    0.8809   -2.6553    1.7837 C   0  0
    1.1831    2.3666   -0.8203 C   0  0
   -2.3472    2.1492    0.1600 C   0  0
   -2.5333    3.1828    0.9896 C   0  0
   -1.7817    3.0600    2.2430 S   0  0
   -1.1584    1.7687    1.9327 C   0  0
    3.1809    0.6235   -0.3289 H   0  0
    2.8284   -1.6595   -1.5037 H   0  0
    2.8216   -1.7862    0.2649 H   0  0
    0.9607   -3.0541   -1.0463 H   0  0
   -1.9859   -0.5460   -0.1458 H   0  0
   -0.7393    0.8025   -1.8931 H   0  0
   -0.8803   -1.6877   -2.0400 H   0  0
    0.6686   -1.1055   -2.6885 H   0  0
   -1.8578   -2.7315   -0.3471 H   0  0
   -0.8837   -4.0419    0.3489 H   0  0
   -1.8225   -2.9542    1.4234 H   0  0
    0.2597   -2.8450    2.6885 H   0  0
    1.3497   -3.6220    1.4905 H   0  0
    1.6861   -1.9643    2.1179 H   0  0
    2.2092    2.7587   -0.6434 H   0  0
    0.8447    2.7581   -1.8061 H   0  0
    0.5312    2.7966   -0.0310 H   0  0
   -2.8106    2.0329   -0.8287 H   0  0
   -3.1809    4.0419    0.7666 H   0  0
   -0.4724    1.2716    2.6320 H   0  0
  2  1  2
  1  8  1
  1 13  1
  3  2  1
  2 18  1

```
  3  4  1
  3 19  1
  3 20  1
  4  5  1
  9  4  1
  4 21  1
  5  6  1
  5 11  1
  5 12  1
  6  7  1
  8  7  1
  7 10  1
  7 22  1
  8  9  1
  8 23  1
  9 24  1
  9 25  1
 14 10  4
 10 17  4
 11 26  1
 11 27  1
 11 28  1
 12 29  1
 12 30  1
 12 31  1
 13 32  1
 13 33  1
 13 34  1
 15 14  4
 14 35  1
 15 16  4
 15 36  1
 17 16  4
 17 37  1
M  END
$$$$
```

References
1. Sidorenko, A.Yu.; Aho, A.; Ganbaatar, J.; Batsuren, D.; Utenkova, D.B.; Sen'kov, G.M.; Wärnå, J.; Murzin, D.Yu.; Agabekov, V.E. Catalytic isomerization of $\alpha$-pinene and 3-carene in the presence of modified layered aluminosilicates. *Mol. Catal.* **2017**, *443*, 193-202.
2. Lountos, G.T.; Zhao, X.Z.; Kiselev, E.; Tropea, J.E.; Needle, D.; Pommier, Y.; Burke, T.R.; Waugh, D.S. Identification of a ligand binding hot spot and structural motifs replicating aspects of tyrosyl-DNA phosphodiesterase I (TDP1) phosphoryl recognition by crystallographic fragment cocktail screening. *Nucleic Acids Res.* **2019**, *47*, 10134–10150.
3. Berman, H.M.; Westbrook, J.; Feng, Z.; Gilliland, G.; Bhat, T.N.; Weissig, H.; Shindyalov, I.N.; Bourne, P.E. The Protein Data Bank. *Nucleic* Acids Res. **2000**, *28*, 235-242.
4. Berman, H.; Henrick, K.; Nakamura, H. Announcing the Worldwide Protein Data Bank. *Nat.Struct.Biol.* **2003**, *10*, 980.
5. Scigress: Version FJ 2.6 (EU 3.1.7) Fujitsu Limited 2008-2016.
6. Allinger, N.L. Conformational Analysis. 130. MM2. A hydrocarbon force field utilizing V1 and V2 torsional terms. *J. Am. Chem .Soc.* **1977**, *99*, 8127-8134.
7. Jones, G.; Willet, P.; Glen, R.C.; Leach, A.R.; Taylor, R. Development and validation of a genetic algorithm for flexible docking. *J. Mol. Biol.* **1997**, *267*, 727-748.
8. Eldridge, M.D.; Murray, C.; Auton, T.R.; Paolini, G.V.; Mee, P.M. Empirical scoring functions: i. the development of a fast empirical scoring function to estimate the binding affinity of ligands in receptor complexes. *J. Comp. Aid. Mol. Design* **1997**, *11*, 425-445.
9. Verdonk, M.L.; Cole, J.C.; Hartshorn, M.J.; Murray, C.W.; Taylor, R.D. Improved protein-ligand docking using GOLD. *Proteins* **2003**, *52*, 609-623.
10. Korb, O.; Stützle, T.; Exner, T.E. Empirical scoring functions for advanced protein−ligand docking with PLANTS. *J.Chem.Inf.Model.* **2009**, *49*, 84-96.
11. Mooij, W.T.M.; Verdonk, M.L. General and targeted statistical potentials for protein–ligand interactions. *Proteins* **2005**, *61*, 272-287.
